# Mechanistic Studies on a Sulfoxide Transfer Reaction Mediated by Diphenyl Sulfoxide/Triflic Anhydride

**DOI:** 10.1002/chem.201102861

**Published:** 2012-01-31

**Authors:** Martin A Fascione, Sophie J Adshead, Pintu K Mandal, Colin A Kilner, Andrew G Leach, W Bruce Turnbull

**Affiliations:** [a] School of Chemistry, University of LeedsLeeds, LS2 9 JT (UK), Fax: (+44) 1133436565 E-mail: w.b.turnbull@leeds.ac.uk; [b] AstraZenecaAlderley Park, Macclesfield, Cheshire, SK10 4TF (UK)

**Keywords:** cations, oxidation, reaction mechanisms, sulfoxides, trifluoromethanesulfonic anhydride

## Abstract

Sulfoxides are frequently used in organic synthesis as chiral auxiliaries and reagents to mediate a wide variety of chemical transformations. For example, diphenyl sulfoxide and triflic anhydride can be used to activate a wide range of glycosyl donors including hemiacetals, glycals and thioglycosides. In this way, an alcohol, enol or sulfide is converted into a good leaving group for subsequent reaction with an acceptor alcohol. However, reaction of diphenyl sulfoxide and triflic anhydride with oxathiane-based thioglycosides, and other oxathianes, leads to a different process in which the thioglycoside is oxidised to a sulfoxide. This unexpected oxidation reaction is very stereoselective and proceeds under anhydrous conditions in which the diphenyl sulfoxide acts both as oxidant and as the source of the oxygen atom. Isotopic labelling experiments support a reaction mechanism that involves the formation of oxodisulfonium (S-O-S) dication intermediates. These intermediates undergo oxygen-exchange reactions with other sulfoxides and also allow interconversion of axial and equatorial sulfoxides in oxathiane rings. The reversibility of the oxygen-exchange reaction suggests that the stereochemical outcome of the oxidation reaction may be under thermodynamic control, which potentially presents a novel strategy for the stereoselective synthesis of sulfoxides.

In memory of Professor David Y. Gin

## Introduction

Chemical transformations involving sulfoxides often involve reaction with a strong electrophile such as triflic anhydride (Tf_2_O) to activate the sulfoxide for reaction with nucleophiles.^1^ For example, dimethylsulfide ditriflate will react with alkenes, alkynes and arenes to provide vinyl and aryl sulfides and sulfonium ions.^2^ Alternatively, the same activated sulfoxide reagent can oxidise simple alcohols to ketones.^3^ Activated sulfoxides have also had a significant impact on the synthesis of oligosaccharides,^4^ ever since the introduction of sulfoxide glycosyl donors by Kahne in 1989.^5^ More recently, there has been a growing interest in the use of diphenyl sulfoxide and triflic anhydride as a system for activating glycosyl donors.^6^ For example, glycosyl hemiacetals can be activated directly using this combination of reagents;^6^, ^7^ glycals can be oxidised to glycosyl epoxides for reaction with alcohols;^8^ and this method is also often used to activate thioglycosides.4f, 7d, 9

**Scheme 1:**
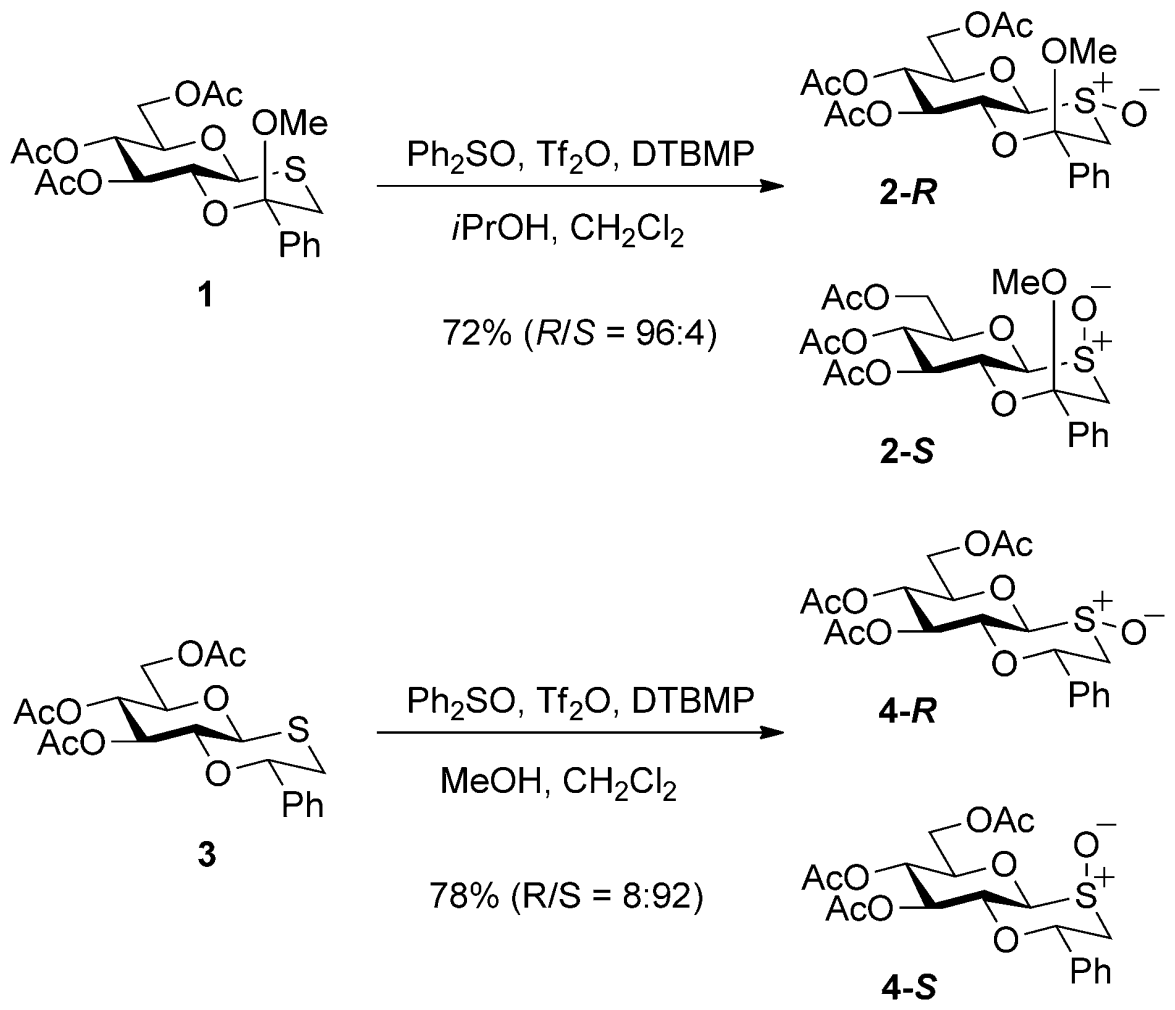
Activation of oxathiane glycosyl donors with diphenyl sulfoxide and triflic anhydride leading to oxidation of the thioglycoside. DTBMP=2,6-di-*tert*-butyl-4-methylpyridine.

We sought to apply the diphenyl sulfoxide/Tf_2_O method for activating oxathiane glycosyl donors **1** and **3** (Scheme 1) that we have recently developed for the preparation of 1,2-*cis*-α-glycosides.^10^ However, no glycoside products were formed when either donor was subjected to standard reaction conditions (diphenyl sulfoxide (2.8 equiv) and Tf_2_O (1.4 equiv))9a, c in the presence of an acceptor alcohol. Instead, both oxathianes were oxidised to the corresponding sulfoxides **2** and **4** in high yield and excellent stereoselectivity.^11^, ^12^ Subsequent experiments on a non-carbohydrate oxathiane showed that the oxidation reaction was not exclusive to glycosides **1** and **3** (see below). Density functional theory calculations (B3LYP/6-31G* and M06/6-31G*)^13^ indicated that in each case the major isomer was also the thermodynamically preferred product (see the Supporting Information). Very similar results were obtained when the oxidation reactions were performed in the absence of an acceptor alcohol, thus demonstrating that the alcohols were superfluous to the reactions. The lack of glycoside products in these reactions is consistent with the unusually high stability of sulfonium ions derived from glycosyl oxathiane compounds.10a, c Nevertheless, the mechanism by which the oxathiane compounds became oxidised was still intriguing.

Oxygen-transfer reactions between sulfoxides and sulfides^14^ (or between selenoxides and sulfides^15^) are well documented in the literature. While spontaneous oxygen transfer can occasionally occur at high temperature,14c in general, the sulfoxide is usually activated by a strong acid under aqueous conditions.14a,b,f A chlorosulfonium ion intermediate has been implicated when HCl is used as the catalyst.14d,e,h However, in the case of less nucleophilic anions, for example, perchlorate or sulfate, it has been postulated that the reaction may involve direct attack by the sulfide on a protonated sulfoxide intermediate.14f Similar mechanisms have also been proposed for oxygen transfer promoted by trifluoroacetic anhydride.14g

It is usually assumed that hydrolysis of a sulfoxonium intermediate would install the oxygen atom in the sulfoxide product.14d–h However, a low temperature ^1^H NMR study of the reaction involving oxathiane **1** revealed that the sulfoxide products **2** ***R*****/2*S*** were formed under anhydrous conditions, and within minutes of the addition of Tf_2_O. Therefore, in the diphenyl sulfoxide/Tf_2_O reaction reported here, it would appear that the oxygen atom must originate from one of these two activating agents. We reasoned that an isotope labelling study could cast further light on the mechanism of oxygen transfer, and possibly even provide insight to the early steps of the glycosylation reactions promoted by diphenyl sulfoxide/Tf_2_O. Herein we will address three important questions: 1) From where does the oxygen atom in the sulfoxide product originate? 2) Which species acts as the oxidant in these reactions? 3) What is the basis for the high stereoselectivity of these reactions? But first, it will be instructive to consider the first step of the oxidation process in some detail: that is, the reaction between diphenyl sulfoxide and Tf_2_O.

## Results and Discussion

**Scheme 2:**
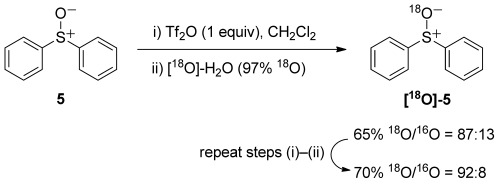
Preparation of [^18^O]-diphenyl sulfoxide.

**Reaction of diphenyl sulfoxide with triflic anhydride**: Several methods for the preparation of ^18^O-enriched diphenyl sulfoxide have been reported in the literature.^16^ We chose to activate the sulfoxide **5** with Tf_2_O (1 equiv), and then quench the reaction with ^18^O-enriched water (97 % ^18^Oincorporation; Scheme 2). The diphenyl sulfoxide isolated from this reaction was found to have an ^18^O/^16^O ratio of 87:13 (see the Supporting Information). After re-subjecting this product mixture to the same reaction conditions, the ^18^O incorporation increased to 92 %. The efficiency of these labelling reactions was found to be reproducible (±2 %).

Incomplete transfer of the ^18^O-label indicated that either a portion of diphenyl sulfoxide failed to react with Tf_2_O on the timescale of the reaction, or the reaction was not completely anhydrous, or an activated sulfoxide intermediate was hydrolysed on work-up with retention of the ^16^O-atom.^17^ IR spectroscopy was used to monitor the progress of the reaction in situ. Analysis of the spectra by using ConcIRT™ (Mettler Toledo) revealed that diphenyl sulfoxide was consumed fully within a minute of adding one equiv Tf_2_O at −32 °C, and that the reaction mixture comprised at least three species at equilibrium (see the Supporting Information).

**Scheme 3:**
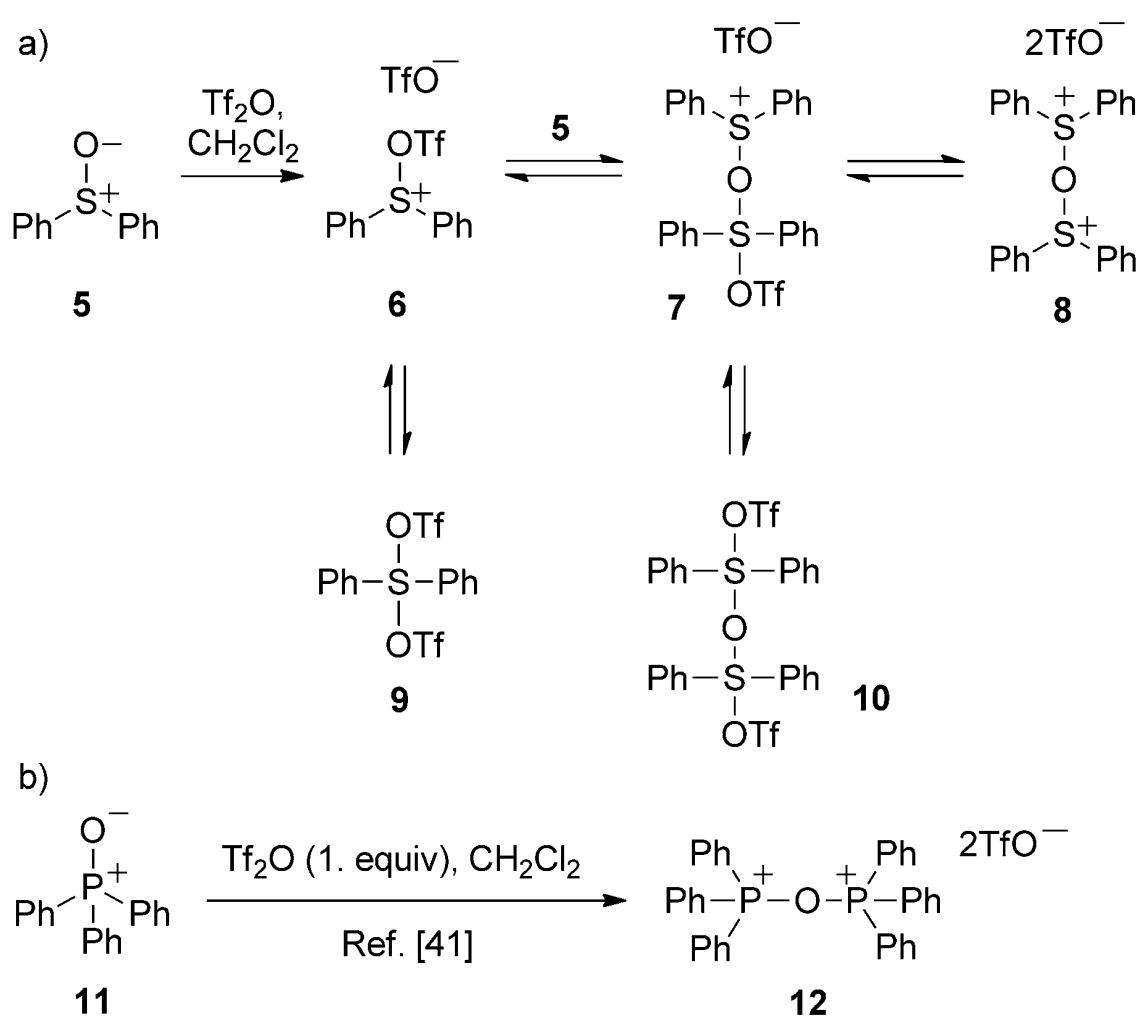
a) Possible intermediates arising from the reaction of diphenyl sulfoxide and triflic anhydride. b) Preparation of Hendrickson’s reagent.^18^

We reasoned that identification of these intermediates would be helpful if we were to understand the oxidation reaction in Scheme 1. The triflyloxy sulfonium ion **6** (Scheme 3 a), which is presumably formed first in the reaction mixture, could combine with its triflate anion to give sulfurane **9**, or it could react with another molecule of diphenyl sulfoxide **5** to yield dication **8**, or the analogous mono- or bis-sulfurane compounds **7** and **10**. Oxo-bridged disulfonium salt **8** has been proposed previously as a reaction intermediate when an excess of diphenyl sulfoxide is mixed with Tf_2_O.8c However, this intermediate could also form when equimolar amounts of the sulfoxide and Tf_2_O are mixed, if the Tf_2_O were added dropwise, and/or intermediate **6** were more reactive as an electrophile than Tf_2_O; for example, the phosphorus analogue, bis(triphenyl)-oxodiphosphonium triflate **12** (Hendrickson’s reagent, Scheme 3 b),^18^ can be prepared by mixing triphenylphosphine oxide **11** and Tf_2_O in equimolar amounts.18a Kelly and co-workers used X-ray crystallography studies to prove that Hendrickson’s reagent is indeed a P-O-P dication.18a

**Figure 1:**
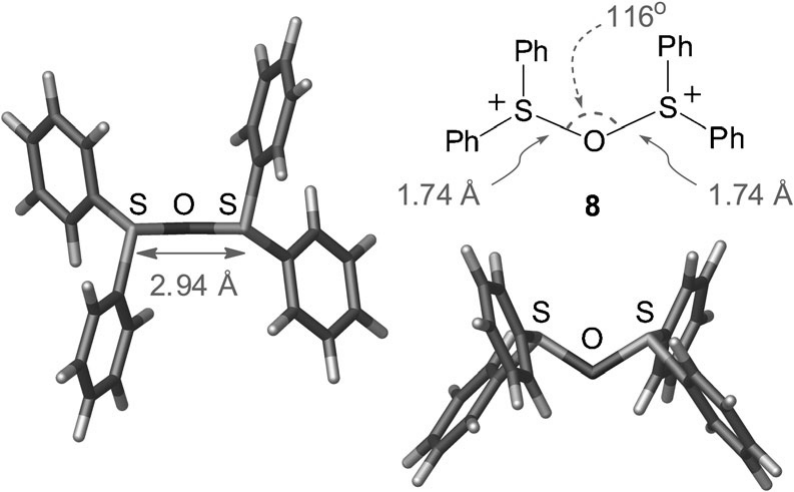
Significant bond lengths and angles derived from density functional theory calculations (B3LYP/6-31G*) on putative reaction intermediates. Plan and elevation views of oxodisulfonium ion **8**.

Density functional theory calculations (B3LYP/6-31G* and M06/6-31G*)^13^ support the hypothesis that all of the intermediates proposed in Scheme 3 are feasible structures with the exception of bis-sulfurane **10** which exhibited no barrier to dissociation in the gas phase. Monocation **6** and dication **8** displayed very similar S–O bond lengths and S–O–S bond angles which place the sulfur atoms in dication **8** only 2.94 Å apart (Figure 1). Dications with the structure [R_2_S-SR_2_]^2+^ are well documented in the literature^19^ (see below) and it would appear that there may be a similar bonding interaction between the two positively charged sulfur atoms in dication **8**.^20^

**Figure 2:**
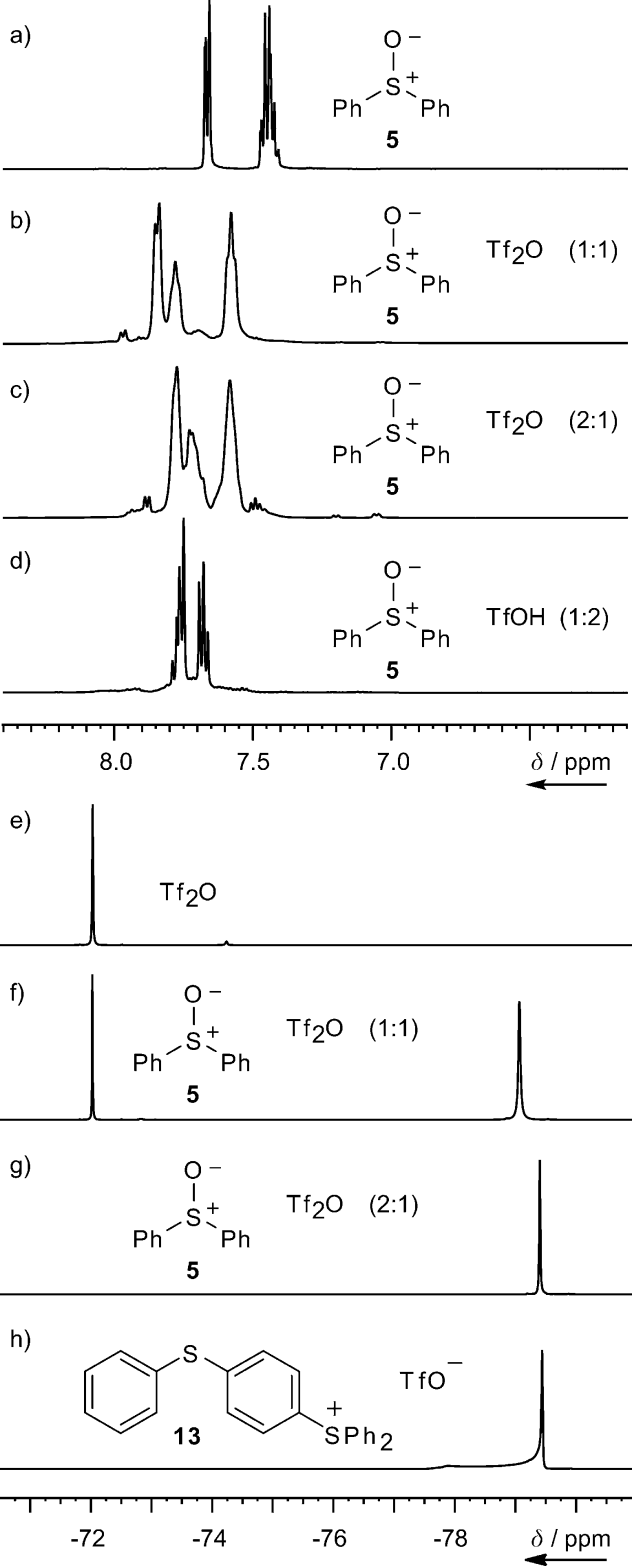
All ^1^H NMR spectra [(a)–(d) 500 MHz; CD_2_Cl_2_], and ^19^F NMR spectra [(e)–(h) 470 MHz; CD_2_Cl_2_] were recorded at −60 °C, except spectrum (d) which was acquired at −30 °C. In each case, several spectra were acquired to check that the system had come to equilibrium. The shoulder at −78 ppm in (h) is a shimming artefact.

^1^H NMR spectroscopy was used to further characterise 1:1 and 2:1 mixtures of diphenyl sulfoxide **5** and freshly distilled Tf_2_O in CD_2_Cl_2_ at −60 °C (Figure 2). In each case, the ^1^H NMR spectrum revealed that all of the diphenyl sulfoxide had been consumed, to produce one or more products displaying broadened NMR signals. While the 1:1 (Figure 2 b) and 2:1 (Figure 2 c) mixtures were dominated by different species (presumably **6**/**9** and **7**/**8**, respectively), the 1:1 mixture also displayed some minor signals (e.g., at 7.7 ppm) that could be consistent with the partial formation of oxodisulfonium ion **8**. Addition of water to the NMR samples converted each mixture to a single species (Figure 2 d) that differed from the original spectrum of diphenyl sulfoxide (Figure 2 a). We attribute these signals to protonation of the sulfoxide by TfOH.^17^

The reaction mixtures were also analysed by ^19^F NMR spectroscopy. The 2:1 mixture showed only a single peak in its ^19^F NMR spectrum at −79.4 ppm (Figure 2 g), with a frequency typical for a triflate anion, for example, sulfonium triflate **13** (Figure 2 h).^21^ In contrast, the 1:1 mixture displayed two signals at −72.0 ppm and −79.1 ppm (Figure 2 f). The signal at −72.0 ppm corresponds to unreacted Tf_2_O (Figure 2 e), and is consistent with partial formation of the dimeric sulfonium ion **8**. While one might expect to see several other fluorine signals corresponding to triflyl/triflate groups in species **6**–**9**, the sole broadened signal at −79.1 ppm would suggest that all triflyl groups are in rapid-intermediate exchange on the NMR timescale.^22^ In summary, the ^19^F NMR spectra indicate that the triflyloxy group in **6** is in fast to intermediate exchange with the triflate counter ion, presumably through an equilibrium with sulfurane **9**. However, any equilibrium between oxygen-bridged species **7**/**8** lies in favour of dication **8** and free triflate anions.

**Scheme 4:**
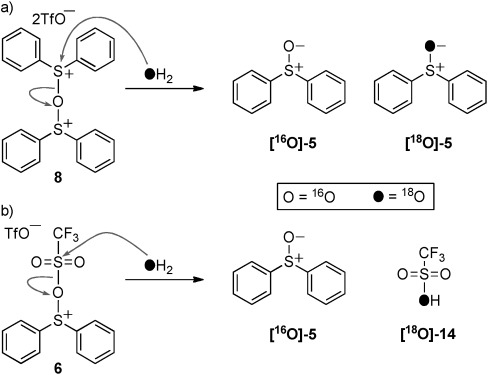
Possible mechanisms for hydrolysis of activated sulfoxide intermediates with retention of ^16^O. Reactions are depicted as S_N_2 processes for convenience but may involve addition–elimination mechanisms via sulfurane intermediates.

As the NMR spectra (Figure 2) demonstrate that all of the diphenyl sulfoxide was consumed under the conditions of the labelling experiment (Scheme 2), the sub-optimal incorporation of the ^18^O-label in **[^18^O]-5** must arise from either less than perfect anhydrous conditions or partial retention of a ^16^O-atom present in one of the activated intermediates (Scheme 4). In the case of dication **8**, attack by [^18^O]-water at either sulfonium centre would result in retention of a ^16^O-atom, while for sulfonium ion **6**, the labelled water would have to attack the S(VI) centre to avoid introducing the label into the diphenyl sulfoxide product; [^18^O]-TfOH **14** would also be produced as a by-product of this reaction. Gin has demonstrated that glycosyl hemiacetals react with sulfonium ions such as **6** at the S(IV) centre only;7a, 23 therefore, the reaction in Scheme 4 b is unlikely to occur.

**Scheme 5:**
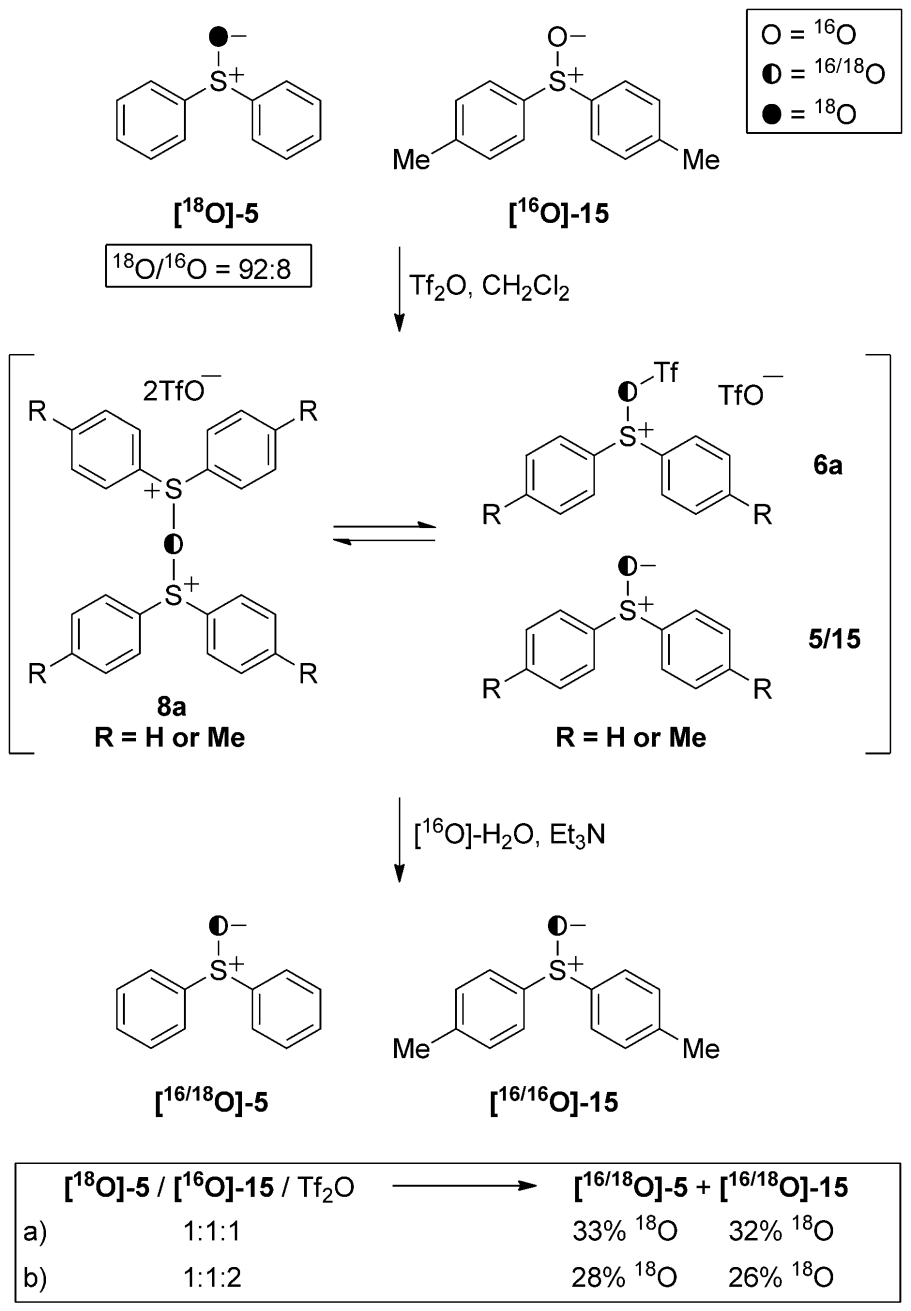
Cross-over experiments in which 92 %-enriched diphenyl sulfoxide **[^18^O]-5**, unlabelled **15** and Tf_2_O are mixed according to the given ratios.

A cross-over experiment was performed to gain further evidence for the formation of oxodisulfonium ion **8**. A mixture of labelled diphenyl sulfoxide **[^18^O]-5** (92 % ^18^O), unlabelled ditolyl sulfoxide **[^16^O]-15** and Tf_2_O (1:1:1 or 1:1:2) was allowed to react at −60 °C for 10 min before addition of unlabelled water and neutralisation with triethylamine (Scheme 5). In theory, when sulfoxides **[^18^O]-5**, **[^16^O]-15** and Tf_2_O are mixed in a 1:1:1 ratio, there is an equal probability that the Tf_2_O will react with diphenyl sulfoxide or ditolyl sulfoxide to give intermediates **6 a**. The NMR experiments in Figure 2 indicate that **6 a** would then react with the remaining 1:1 mixture of sulfoxides **5** and **15** to produce oxodisulfonium ions **8 a**. Half of the bridging oxygen atoms in **8 a** should thus be derived from **[^18^O]-5**, and half from **[^16^O]-15**. Therefore, the ^18^O-enrichment in oxodisulfonium ions **8 a** should be half that of the original [^18^O]-diphenyl sulfoxide (i.e., 92 %÷2=46 %). Upon aqueous work-up, intermediates **8 a** will be hydrolysed to reform sulfoxides **5** and **15**. Half of the molecules should retain the bridging oxygen atom from **8 a**, while the other half would receive an oxygen atom from H_2_O. Therefore, for a 1:1:1 mixture of **[^18^O]-5**, **[^16^O]-15** and Tf_2_O undergoing oxodisulfonium-mediated ^18^O-exchange, the theoretical ^18^O-enrichment in the product sulfoxides **5** and **15** should be 23 % (i.e., 46 %÷2).

In the case of the 1:1:2 mixture of [^16^O]-**5**, [^18^O]-**15** and Tf_2_O, one might expect species **6 a** to dominate the reaction mixture. Transfer of ^18^O label between diphenylsulfoxide and ditolyl sulfoxide could still occur through exchange of labelled triflate ions. However, the label would only be retained in the sulfoxide products **5** and **15** after aqueous work-up if the triflyl groups could be removed as shown in Scheme 4 b. This process has already been discounted as unlikely to occur,7a however, if it did happen, the ^18^O-incorporation in the product sulfoxides **5** and **15** would be very low. Half of the Tf_2_O would react initially with **[^18^O]-5**, and the other half with **[^16^O]-15** to produce triflyloxysulfonium ions **6 a** that are partially labelled (i.e., 92 %÷2=46 % ^18^O) and unlabelled triflate anions. Triflate exchange would scramble all 12 oxygen atoms in the triflyloxy and triflate groups further reducing the labelling in ions **6 a**.^24^ Therefore, for a 1:1:2 mixture of **[^18^O]-5**, **[^16^O]-15** and Tf_2_O undergoing triflate-mediated ^18^O-exchange, the theoretical ^18^O-enrichment in product sulfoxides **5** and **15** should be less than 8 % (i.e., 92 % ^18^O÷12 oxygen atoms=7.7 %).

Both experiments led to an even distribution of the ^18^O label between diphenyl sulfoxide and ditolyl sulfoxide. Furthermore, the incorporation of ^18^O label in the reaction products was found to be higher than expected (32–33 % ^18^O for 1:1:1 mixture; 26–28 % for 1:1:2 mixture). In other words, cross-over of the ^18^O-label occurred without any apparent scrambling of oxygen atoms that would be associated with a triflate-exchange mechanism. These results are only consistent with a reversible ^18^O-exchange process involving diaryl sulfoxides **5**/**15** and oxygen-bridged species **8 a**. The formation of an oxodisulfonium ion intermediate **8 a** upon mixing diaryl sulfoxide and Tf_2_O in equimolar amounts would be in accord with formation of Hendrickson’s reagent under similar conditions (Scheme 3 b).18a Furthermore, the enhanced label incorporation in the 1:1:1 reaction mixture could be possible if intermediate **8 a** were to be quenched by a side reaction prior to aqueous work-up (see below), thus avoiding further dilution of the ^18^O label with **[^16^O]-**H_2_O.

Several conclusions can be drawn from these preliminary studies on diphenyl sulfoxide and Tf_2_O: (1) triflyloxy sulfonium ion **6** can react rapidly at its sulfur(IV) centre with diphenyl sulfoxide to generate oxodisulfonium ion **8**; (2) the oxodisulfonium ion **8** dominates the reaction mixture when diphenyl sulfoxide and Tf_2_O are mixed in a ratio of 2:1; (3) oxodisulfonium ions can undergo an exchange reaction with other sulfoxides.

**Figure 3:**
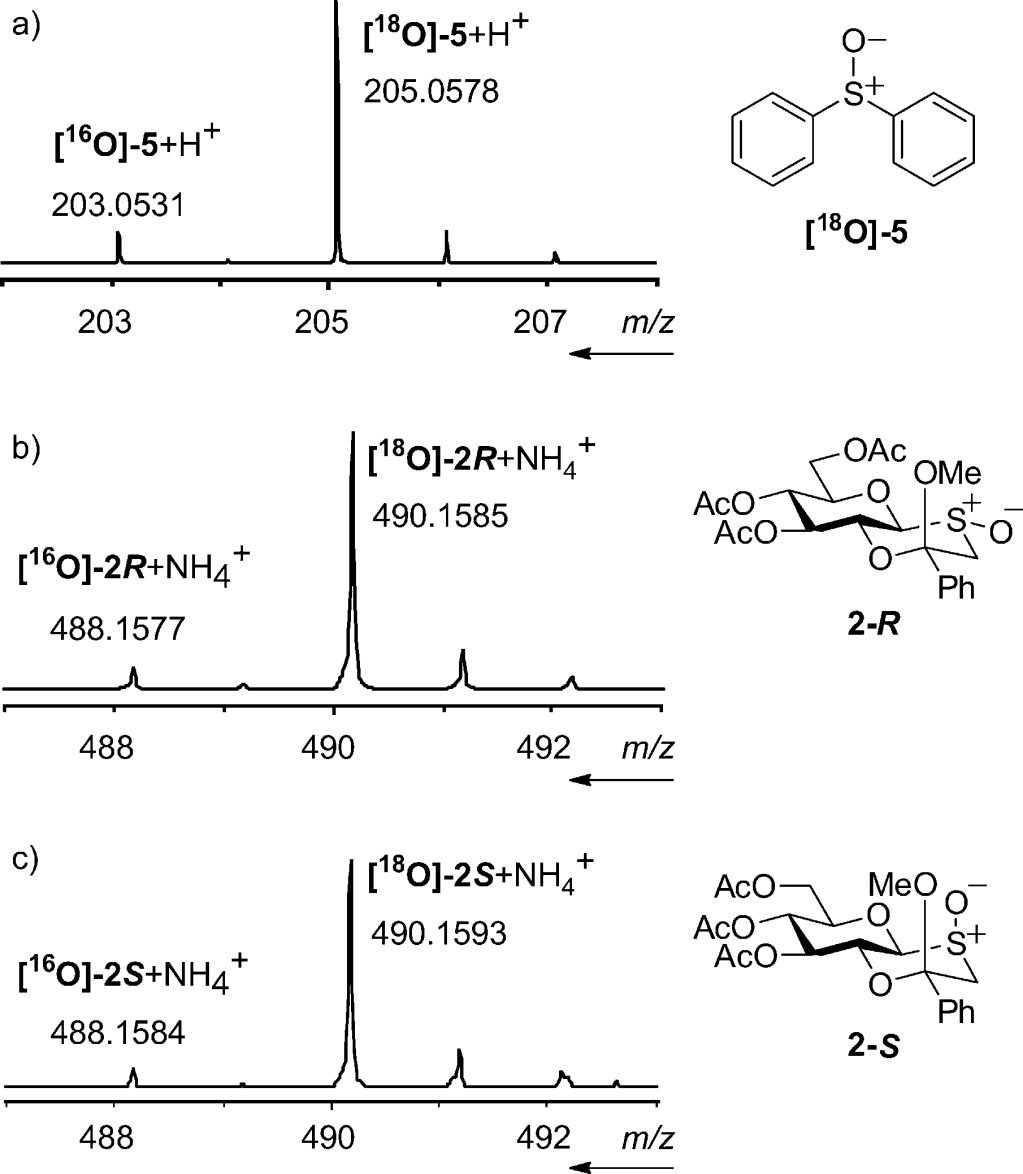
Mass spectra for a) [^18^O]-diphenyl sulfoxide reactant, b) oxathaine **2-*R*** and c) oxathiane **2-*S*** products of the oxidation reaction.

**Origin of the oxygen atom in the oxathiane-*S*-oxide product**: With [^18^O]-diphenyl sulfoxide in hand, we sought to test the hypothesis that oxidation of the oxathiane involved transfer of oxygen from diphenyl sulfoxide. [^18^O]-Diphenyl sulfoxide **5** (87 % ^18^O) and Tf_2_O were used to oxidise oxathiane ketal **1** as described above. The resulting axial and equatorial sulfoxides were separated by silica gel chromatography for mass spectrometric analysis. Both isomers of the oxathiane-*S*-oxides (**2-*R*** and **2-*S***) were found to have 87 % ^18^O-enrichment (Figure 3). The excess diphenyl sulfoxide **5** that was recovered from the reaction mixture also had the same ^18^O-content as the starting material.

Two things can be concluded from this experiment: 1) The oxygen atom arises solely from the diphenyl sulfoxide, and not from either the Tf_2_O or water on aqueous work-up. 2) The reaction reaches completion before aqueous work-up, that is, there can be no activated sulfoxide species remaining at the time of work-up, or the percentage of ^18^O-label incorporation would be reduced, at very least in the case of the recovered diphenyl sulfoxide.

An identical labelling experiment using oxathiane ether **3** also afforded sulfoxide product **4-*S*** with a very high level of ^18^O-incorporation (83 %). On this occasion, it was not possible to isolate the equatorial sulfoxide isomer **4-*R*** as it was formed in very low concentration. It is not clear if the small reduction in the isotopic enrichment (87 % to 83 %) is significant in this case. Therefore, we presume that both oxidation reactions in Scheme 1 occur by the same mechanism.

**Scheme 6:**
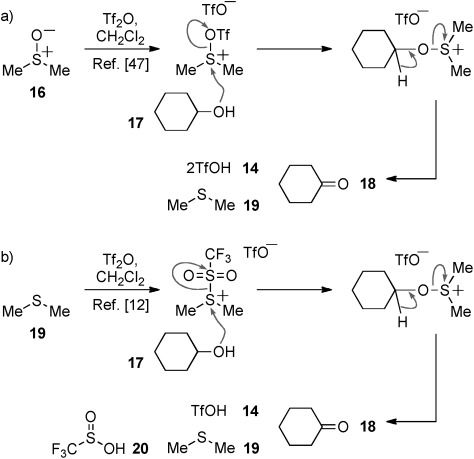
Mechanisms for oxidation reactions promoted by Tf_2_O as a) a sulfoxide activating agent and b) an oxidant. In reaction a)two equiv TfOH **14** are produced, while reaction b) produces 1 equiv TfOH **14** and 1 equiv of sulfinic acid **20**.

**Identification of the oxidizing agent**: Having determined the origin of the oxygen atom in the product, the next question to address was the nature of the oxidising agent; that is, which species becomes reduced during the reaction. Mixtures of Tf_2_O and either dimethyl sulfoxide **16** or diphenyl sulfoxide **5**, have been reported to oxidise alcohols (Scheme 6 a),^25^ and glycals,8c respectively. However, precedent for oxidative transformations using Tf_2_O alone also exists; for example, oxidation of aliphatic sulfides by Tf_2_O can provide access to sulfoxides, unless an alcohol is added to the mixture, in which case aldehydes or ketones will result (Scheme 6 b).^3^

**Figure 4:**
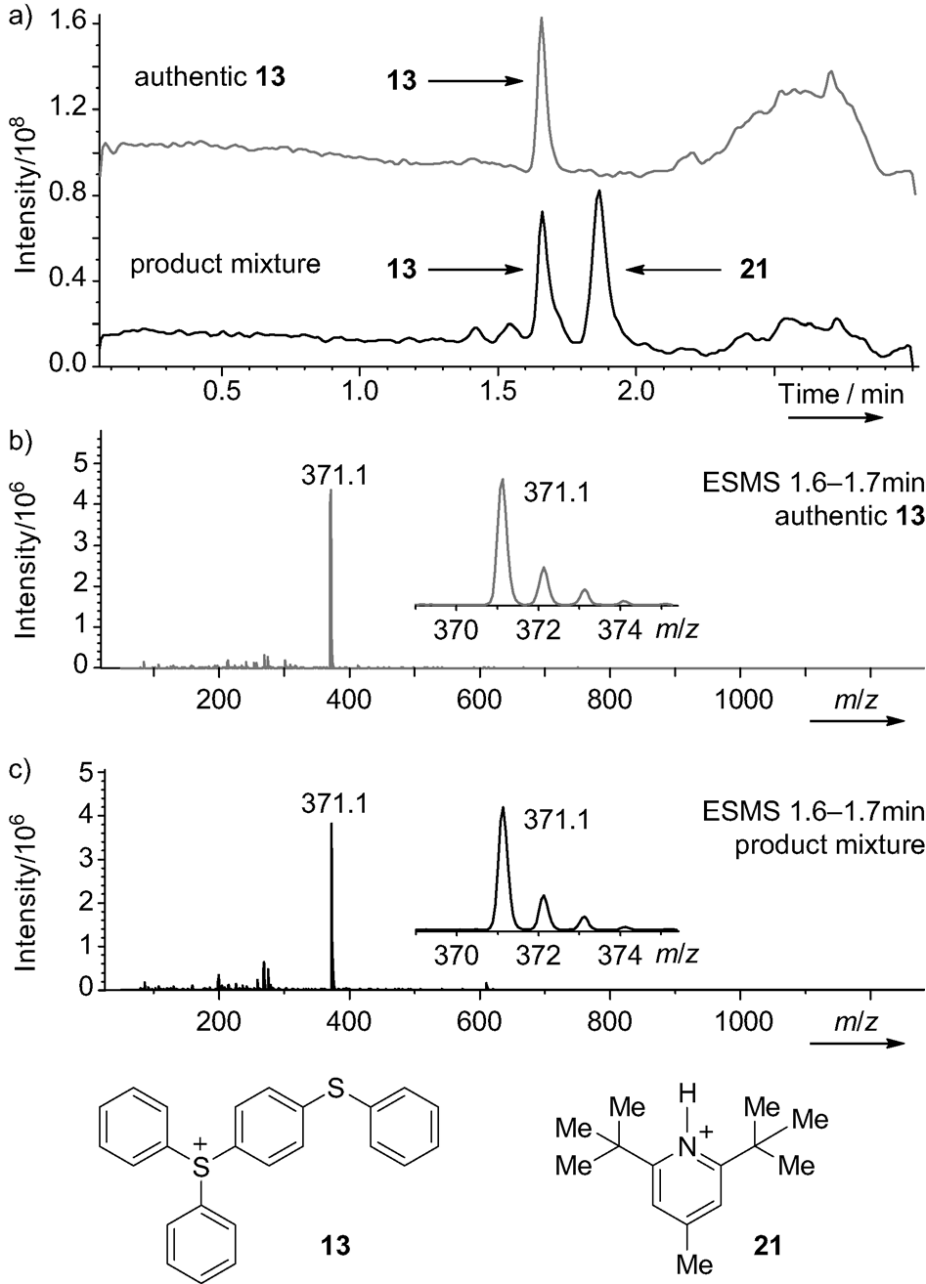
a) LC-MS ion chromatograms for sulfonium triflate **13** and the crude product mixture from a typical oxidation reaction in which DTBMP is used as base forming pyridinium ion **21** as a by-product. Mass spectra for b) ion **13** and c) the component of the reaction mixture eluting between 1.6 and 1.7 min. In each case expansions of the major ions are inset in b) and c).

In cases where Tf_2_O acts as an oxidant, one sulfonate group will become reduced to the sulfinate **20** (Scheme 6 b). On the other hand, if the role of Tf_2_O is solely to activate a sulfoxide, for example, **16** (Scheme 6 a), then the corresponding sulfide **19** should be formed as the reduction product. Although we were never able to observe or isolate diphenyl sulfide from the sulfoxidation reaction, triaryl sulfonium salt **13** was consistently observed in the mass spectra of the crude reaction mixtures (Figure 4). The quantitative formation of aryl sulfonium salt **13** was confirmed by HPLC-mass spectrometric comparison of the crude product mixture with authentic samples of sulfonium salt **13** of known concentration (see the Supporting Information).

Sulfonium salt **13** has been synthesised previously from diphenyl sulfide and diphenyl sulfoxide activated with Tf_2_O and other electrophiles.^26^ Therefore, it is reasonable to conclude that an activated sulfoxide species is responsible for oxidising the oxathiane compounds, while concomitantly producing diphenyl sulfide as a by-product. Any excess activated sulfoxide is then sequestered by the diphenyl sulfide to yield sulfonium ion **13**.^27^ This side-reaction has two consequences: 1) The oxidation reaction is effectively quenched by diphenyl sulfide prior to aqueous work-up (in agreement with the results of ^18^O-labelling experiments). 2) The oxidation reaction cannot become catalytic in Tf_2_O.

**Scheme 7:**
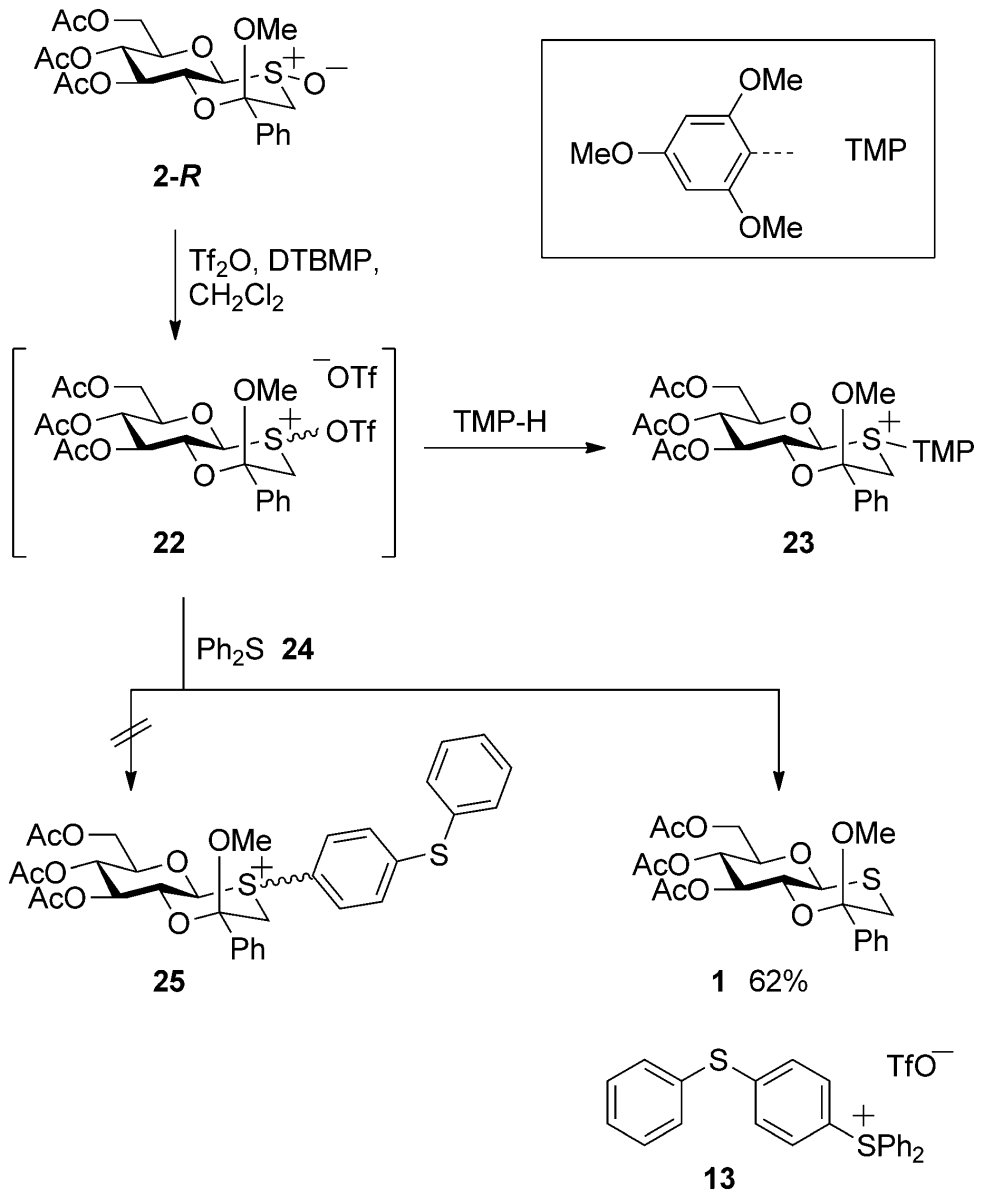
Reaction of oxathiane-*S*-oxide **2-*R*** with Tf_2_O and electron-rich aromatic compounds.

**Reversibility of the oxidation reaction**: We have reported previously that oxathiane-*S*-oxide glycosyl donors can be converted to aryl sulfonium ions (e.g., compound **23**, Scheme 7) by treatment with Tf_2_O in the presence of trimethoxybenzene.10a However, the analogous aryl sulfonium ion **25**, derived from diphenyl sulfide **24**, was not observed as a by-product of the oxidation reaction. One possible explanation would be that the triflyloxy sulfonium ion **22** is not formed during the oxidation mechanism. To test this hypothesis, sulfoxide **2-*R*** was treated first with Tf_2_O (1.1 equiv), and then diphenyl sulfide (2 equiv). The major product isolated from the reaction mixture was oxathiane **1** (62 %). No evidence could be found for the formation of sulfonium ion **25**; however, triaryl sulfonium ion **13** was observed in the mass spectrum of the crude product mixture. This experiment demonstrated that even if triflyloxy sulfonium ion **22** is formed during the oxidation reaction, it would react preferentially with the sulfur atom of diphenyl sulfide, rather than by electrophilic aromatic substitution. Furthermore, reduction of the oxathiane-*S*-oxide **2-*R*** to give oxathiane **1** suggests that the oxidation process is potentially reversible under the conditions of the reaction.

**Scheme 8:**
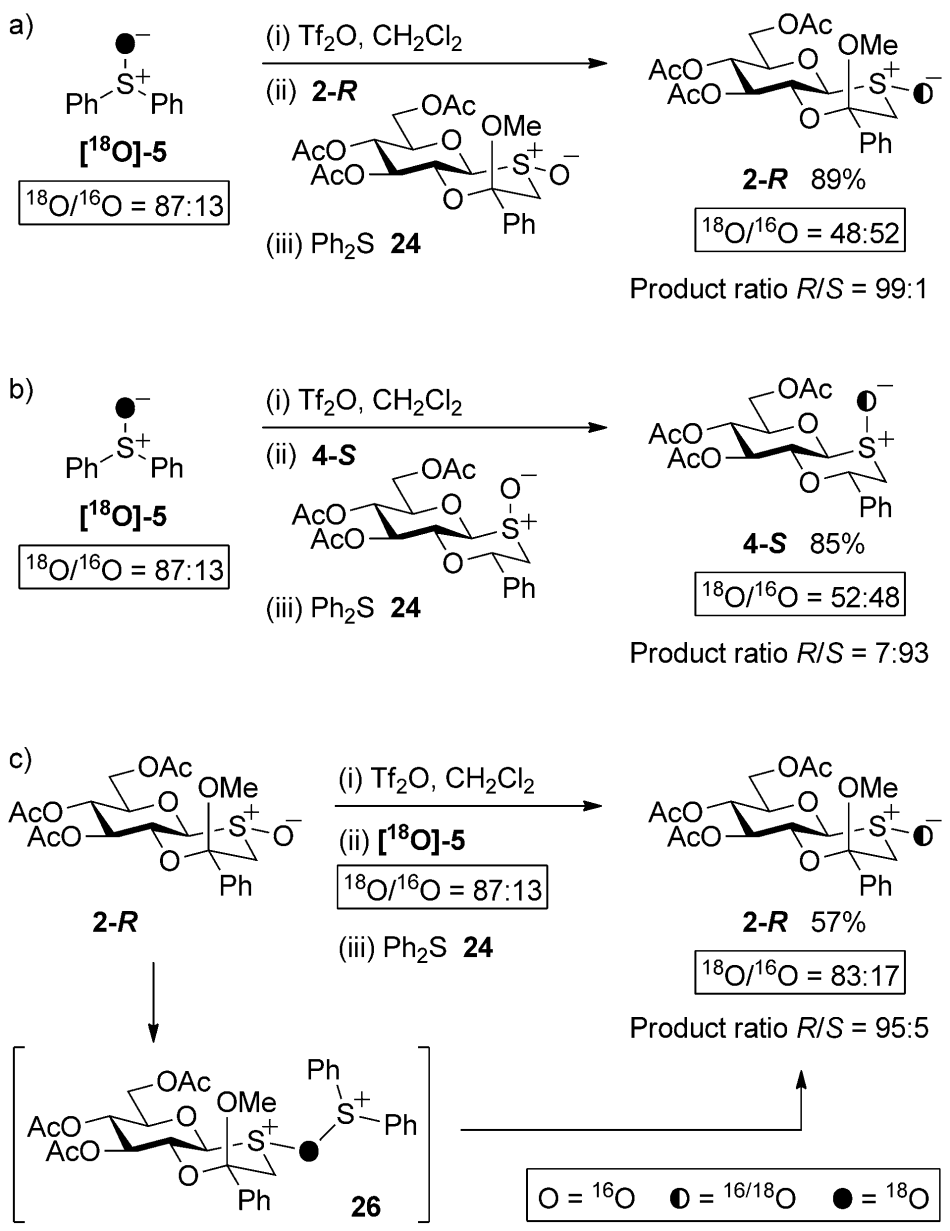
Transfer of ^18^O from **[^18^O]**-**5** to oxathiane-*S*-oxides **2** and **4**: a) and b) pre-activation of diphenyl sulfoxide (2.8 equiv) with Tf_2_O (1.4 equiv). c) Pre-activation of oxathiane-*S*-oxide **2** with Tf_2_O (1.1 equiv) before addition of [^18^O]-diphenyl sulfoxide (2.2 equiv).

We were thus curious to know if the oxygen-transfer process could also be reversible. Therefore equatorial sulfoxide **2-*R*** was re-exposed to the conditions of the oxidation reaction (Scheme 8 a). [^18^O]-Diphenyl sulfoxide (2.8 equiv) was pre-activated with Tf_2_O (1.4 equiv) before adding sulfoxide **2-*R***. After 40 min at −60 °C, the reaction was quenched by addition of diphenyl sulfide. The equatorial sulfoxide isomer **2-*R*** dominated the product mixture and was recovered in 89 % yield. Electrospray mass spectrometry confirmed that the ^18^O-label from the diphenyl sulfoxide had been transferred to oxathiane S-oxide **2-*R*** (^18^O/^16^O=48:52). Excess diphenyl sulfoxide recovered from the reaction mixture (1.34 equiv) had a similar ^18^O-enrichment as **2-*R*** (^18^O/^16^O=44:56).^28^ A very similar result was obtained for oxathiane-*S*-oxide **4-*S*** (Scheme 8 b), except this time the axial sulfoxide was the dominant product. Therefore both of these examples demonstrate that oxygen transfer is a reversible process that can occur with retention of configuration.

As pre-activation of labelled diphenyl sulfoxide results in scrambling of the label with the oxathiane-*S*-oxide oxygen atom, the reaction was also performed by pre-activation of oxathiane-*S*-oxide **2-*R*** with Tf_2_O (1.1 equiv; Scheme 8 c). Once again, NMR spectroscopy of the crude product mixture showed that the equatorial sulfoxide was the dominant product. Furthermore, by reversing the order of addition of sulfoxides **2-*R*** and **[^18^O]-5**, ^18^O-enrichment close to the theoretical maximum was achieved for the equatorial isomer. Incorporation of ^18^O with such high efficiency can only arise through the formation of oxygen-bridged disulfonium ion **26** (Scheme 8 c) as any other mechanism (e.g., ^18^O transfer via triflate exchange) would result in significant dilution of the label.^24^

**Scheme 9:**
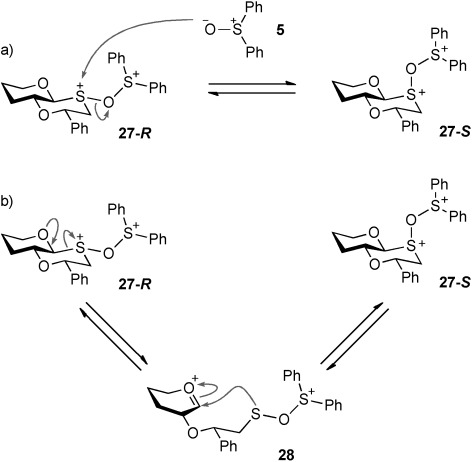
a) Intermolecular and b) intramolecular mechanisms for the inter-conversion of axial and equatorial isomers of oxodisulfonium ion **27-*R*/*S***. The intermolecular mechanism is depicted as an S_N_2 process for simplicity, although it is possible it could involve addition–elimination steps via a sulfurane intermediate.

Retention of configuration at the sulfoxide centre during the ^18^O-crossover experiment requires a mechanism that can allow inter-conversion of axial and equatorial intermediates (Scheme 9). One can imagine two possible mechanisms for such a process: an intermolecular mechanism, in which diphenyl sulfoxide **5** acts as a nucleophile to attack an activated oxodisulfonium ion **27**; and an intramolecular mechanism, in which the anomeric bond is broken to generate an oxacarbenium ion intermediate **28** which would allow reorientation of the substituent on the anomeric sulfur atom. While latter mechanism cannot be excluded for glycosyl sulfoxides, we sought further evidence in support of the intermolecular mechanism.

**Scheme 10:**
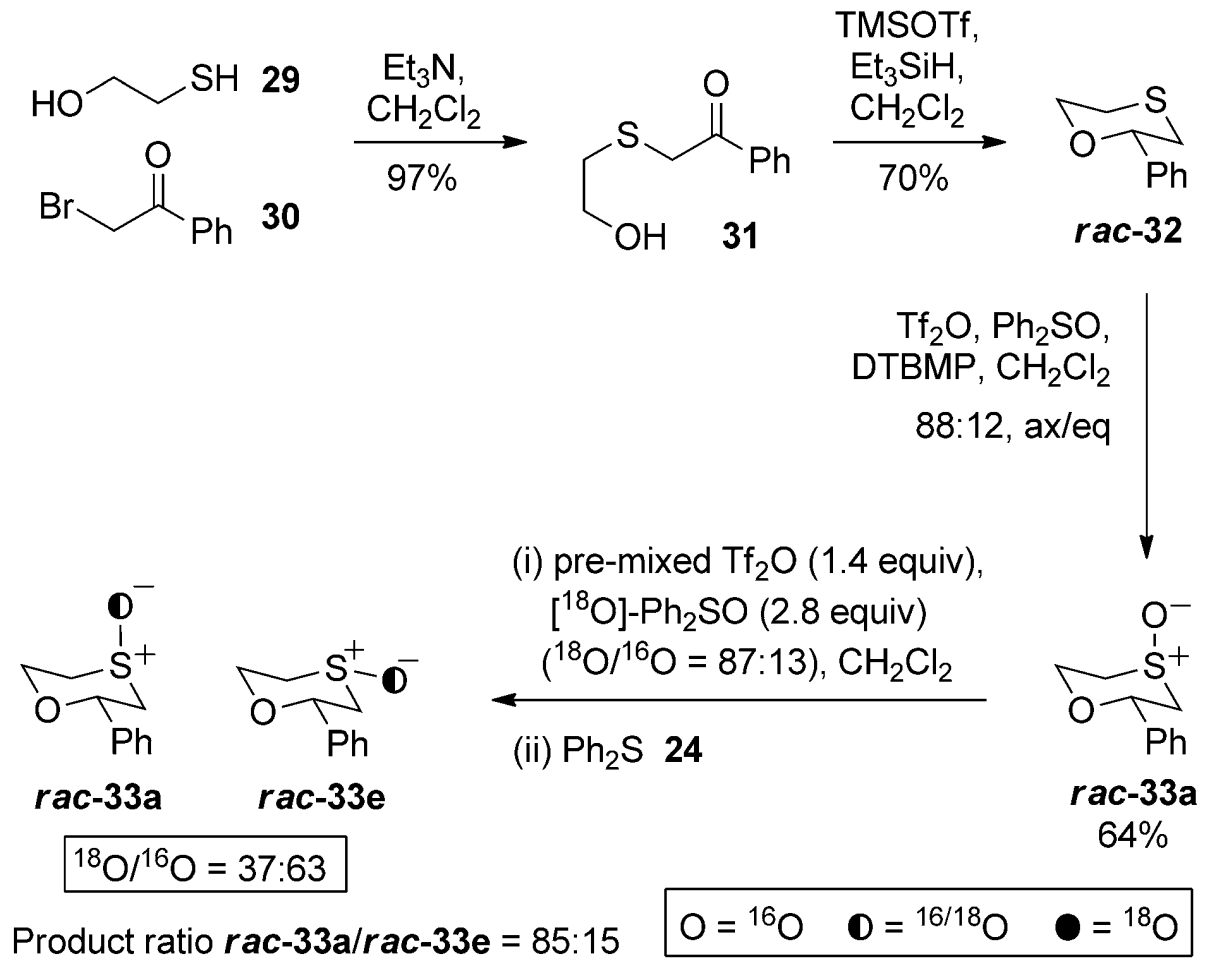
Synthesis of racemic oxathiane-*S*-oxide ***rac*****-33 a** and transfer of ^18^O from **[^18^O]-5** with retention and inversion of configuration.

A simple racemic oxathiane compound ***rac*****-32** lacking the sugar ring was synthesised (Scheme 10). Alkylation of β-mercaptoethanol **29** with bromoacetophenone **30** gave ketone **31**. Reductive cyclisation in the presence of TMSOTf and Et_3_SiH led directly to oxathaine ***rac*****-32** in good yield. Stereoselective oxidation with diphenyl sulfoxide and Tf_2_O proceeded as observed previously for glycosyl oxathiane **3** with the axial isomer ***rac*****-33 a** dominating the crude reaction mixture (88:12, ax/eq).^12^ Careful column chromatography allowed the pure axial isomer ***rac*****-33 a** to be isolated in 64 % yield.

The sulfoxide was added to a pre-formed mixture of labelled diphenyl sulfoxide **[^18^O]-5** (2.8 equiv) and Tf_2_O (1.4 equiv) at −60 °C. The cross-over experiment was quenched after 1.5 h by addition of diphenyl sulfide and NaHCO_3_. NMR spectroscopy demonstrated that the crude product mixture was an 85:15 mixture of the axial and equatorial sulfoxides, while mass spectrometry gave an overall ^18^O-incorporation of 37 %. As the level of ^18^O-incorporation significantly exceeds the quantity of the minor isomer, it must be possible to have oxygen exchange with retention of configuration in this non-carbohydrate system. Therefore the intermolecular mechanism for inversion of configuration is possible. Furthermore, these experiments demonstrate that the oxidation reaction is also applicable to sulfides that lack the sugar ring.

As noted above, the oxidation reaction is highly stereoselective and for each example in Scheme 1 and Scheme 10, the thermodynamically favoured isomer is the dominant product (see the Supporting Information for DFT calculations on each structure). Considering that 1) axial and equatorial sulfoxides can be inter-converted under the conditions of the oxidation reaction, and 2) the major ^18^O-labelled product of the cross-over experiments is the thermodynamically favoured isomer, it is possible that the high stereoselectivity of the oxidation process could result from the reaction occurring under thermodynamic control. Indeed, in each case, re-equilibration of the major isomer results in a product mixture that is very similar to that for the corresponding oxidation reaction. On the other hand, the oxidation reaction is effectively self-quenching; the diphenyl sulfide produced during the reaction sequesters activated sulfoxide intermediates to form sulfonium ion **13**. Therefore, equilibration of sulfoxides and/or reaction intermediates, for example, oxodisulfonium ion **27**, would have to compete with reaction quenching by diphenylsulfide under kinetic control.

**Possible mechanistic pathways**: The aforementioned experiments provide evidence for a number of steps that must occur during the oxidation reaction: the first committed step in the mechanism must be the reaction of the oxathiane sulfur atom with an activated diphenyl sulfoxide species;^29^ a diphenyl sulfoxide oxygen atom must become covalently bound to the oxathiane sulfur atom; diphenyl sulfide must be produced during the reaction, and then react with some activated diphenyl sulfoxide species to produce the triaryl sulfonium salt by-product.

**Scheme 11:**
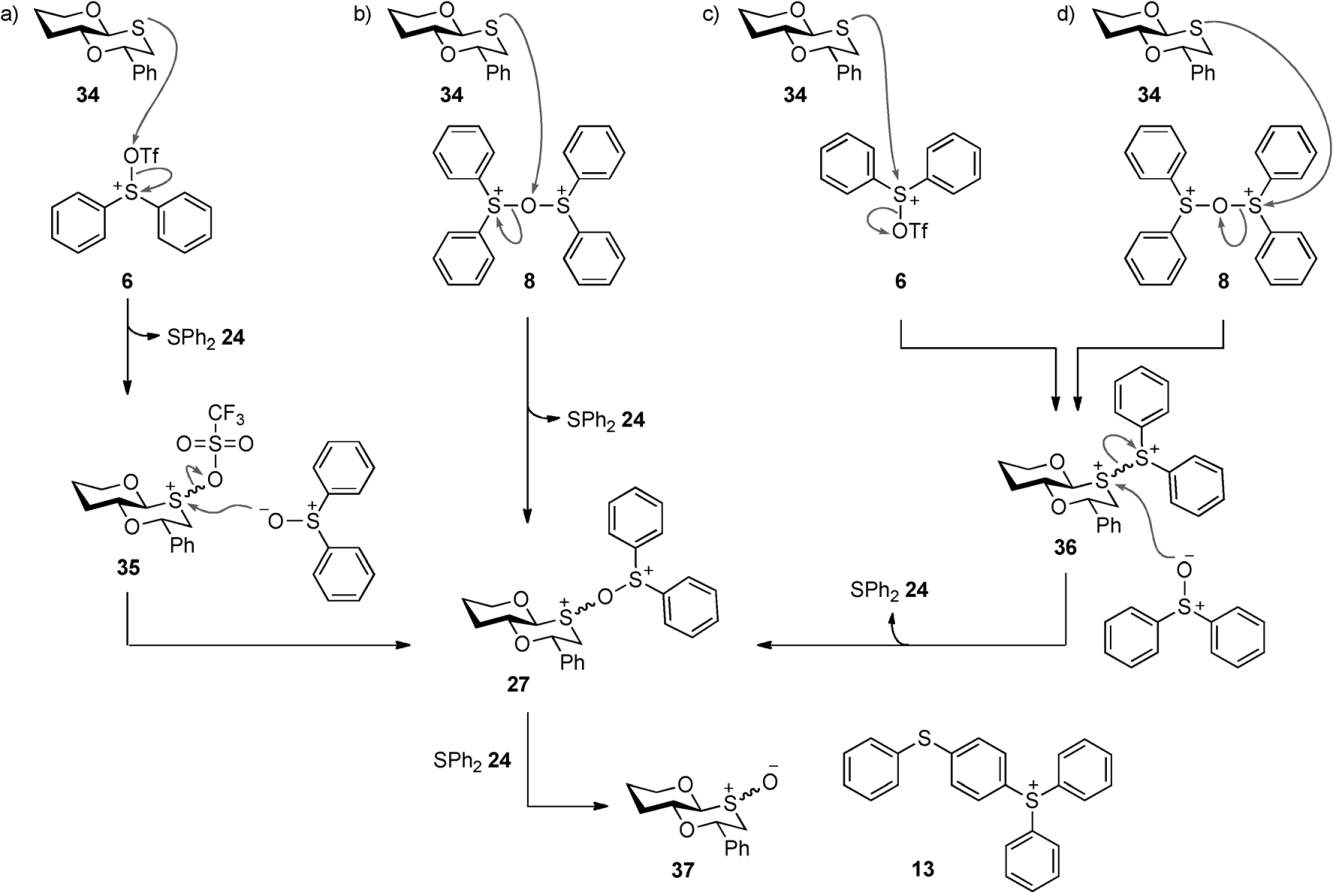
Possible reaction pathways for the oxidation of generic oxathiane **34**. Mechanisms are depicted as S_N_2 processes for simplicity, although it is likely they may involve addition-elimination mechanisms via sulfurane intermediates.

Several mechanistic pathways could be consistent with these observations (Scheme 11). In pathway (a) oxathiane **34** initially attacks an electrophilic oxygen atom in triflyloxy sulfonium ion **6** to produce activated oxathiane **35** and diphenyl sulfide **24**. Activated oxathiane **35** would then react with the excess diphenyl sulfoxide to provide oxodisulfonium ion **27**.^30^ Intermediate **27** could also be formed via route (b) which similarly involves reaction at an electrophilic oxygen atom, but this time in dication **8**. Although there is evidence in the literature for activated sulfoxide species reacting through electrophilic oxygen atoms,^31^ we consider routes (a) and (b) to be less likely than attack at the softer electrophilic sulfur atoms in intermediates **6** and **8** (Scheme 11 c-d).^32^

If oxathiane **34** were to react at the sulfonium centres of cation **6** (route c) or dication **8** (route d), a dithiadication intermediate **36** would be produced. Dithiadications have been the subject of several reviews,^19^ and this type of functional group is invariably synthesised by reaction between a sulfide and an activated sulfoxide.^33^ Dithiadications have also been implicated in the mechanism of “remote Pummerer reactions” and other processes involving transfer of oxygen from sulfoxides to sulfides.14f,g,i, 34

If a dithiadication were to form during the mechanism depicted in Scheme 11, it would still be necessary to introduce the sulfoxide oxygen atom and to release diphenyl sulfide as a by-product. Both of these steps could be achieved by diphenyl sulfoxide attack at the oxathiane sulfur atom in intermediate **36** to provide oxodisulfonium ion **27**. Therefore, regardless of the initial reaction step between thioglycoside **34** and the activated sulfoxide species, all four mechanistic routes could converge on the oxygen-linked dication intermediate **27**.

The final step of the reaction would involve intermediate **27** reacting with diphenyl sulfide **24** to release the oxathiane-*S*-oxide product **37** and form the triaryl sulfonium ion **13**.^35^ In this regard, it is worth reiterating that at no point have we observed the formation of an aryl sulfonium salt combining diphenyl sulfide and the oxathiane (e.g., **25**, Scheme 7). Therefore, if oxygen-linked dication **27** reacts directly with diphenyl sulfide, the reaction must occur very selectively at the diphenyl sulfonium centre in intermediate **27**. This may well be possible as alkyl sulfonium ions are more stable than aryl sulfonium ions.^36^

It is also interesting to note that dithiadications can react with electron-rich aromatic compounds to form aryl sulfonium ions.^37^ It is possible that an analogous reaction could be responsible for the reduction reaction shown in Scheme 7; reaction of diphenyl sulfide with activated sulfoxide **22** could give a dithiadication which might then react with a second molecule of diphenyl sulfide to yield oxathiane **1** and sulfonium salt **13**.

## Conclusion

Oxodisulfonium ions have been proposed previously as reaction intermediates,8c, 31b but to the best of our knowledge, no experimental evidence for their existence has been reported. NMR spectroscopy data and ^18^O-cross-over experiments support the formation of oxodisulfonium ions when sulfoxides and Tf_2_O are mixed at low temperature. Such species undergo exchange reactions with other sulfoxides, thus facilitating oxygen exchange and inter-conversion of axial and equatorial sulfoxides in oxathiane rings.

The commonly used diphenyl sulfoxide/Tf_2_O reagent is able to oxidise sulfides to sulfoxides. In this reaction diphenyl sulfoxide acts as the oxidant (following activation by Tf_2_O) and also transfers the oxygen atom to the sulfide through an oxodisulfonium ion intermediate **27** (Scheme 11). The first step of the oxidation process most probably involves reaction of the sulfide with an activated diphenyl sulfoxide species resulting in formation of a dithiadication intermediate **36**. Either oxodisulfonium ion **8** or triflyloxy sulfonium ion **6** could act as the activating agent. Although oxodisulfonium ion **8** is the dominant species at equilibrium for a 2:1 mixture of diphenyl sulfoxide and Tf_2_O, triflyloxy sulfonium ion **6** is formed first and could thus react directly with the sulfide. Either way, dithiadication **36** would then react with diphenylsulfoxide to introduce the new sulfoxide oxygen atom via oxodisulfonium intermediate **27**. Diphenylsulfide produced in this step as an initial by-product, goes on to sequester activated sulfoxide species, effectively quenching the reaction prior to aqueous work-up.

Sulfoxide exchange with the oxodisulfonium intermediate **27** presents an intriguing possibility that the stereochemical outcome of the oxidation process could occur under thermodynamic control. Of course, this mechanism would require rapid equilibration of the oxodisulfonium intermediates prior to deactivation by diphenylsulfoxide. Without a detailed knowledge of the kinetics of the reaction it is not possible to conclude whether or not the original oxidation reaction is under kinetic or thermodynamic control. Nevertheless, it may be possible that the system could be engineered to slow or remove the diphenyl sulfide termination step, for example by using a substituted diaryl sulfoxide. If so, this oxygen-transfer reaction would present a novel strategy for the stereoselective synthesis of sulfoxides.

## Experimental Section

Full experimental details and analytical data for compounds prepared and coordinates for compounds modelled using DFT calculations are included in the Supporting Information. CCDC-733122 http://www.ccdc.cam.ac.uk/cgi-bin/catreq.cgi(**2-*R***)10a and CCDC-733123 http://www.ccdc.cam.ac.uk/cgi-bin/catreq.cgi(**4-*S***)10c contain the supplementary crystallographic data for this paper. These data can be obtained free of charge from The Cambridge Crystallographic Data Centre via http://www.ccdc.cam.ac.uk/data_request/cif.

**General experimental methods**: All solvents were dried prior to use, according to standard methods.^38^ Trifluoromethanesulfonic anhydride (Tf_2_O), was freshly distilled from phosphorus pentoxide under a N_2_(g) atmosphere and all other commercially available reagents were used as received. All reactions were performed in oven dried glassware under a N_2_(g) atmosphere.

**General procedure for oxidation reaction**: Tf_2_O (1.4 equiv) was added to a solution of sulfide (1 equiv), diphenyl sulfoxide (2.8 equiv) and 2,6-di-*tert*-butyl-4-methylpyridine (DTBMP) (3 equiv) in CH_2_Cl_2_ (sulfide concentration: 250–500 mm) at −60 °C. After 40–90 min at that temperature the reaction was quenched by addition of sat. NaHCO_3_ solution. The mixture was extracted into CH_2_Cl_2_, dried (MgSO_4_) and concentrated in vacuo to give the crude product which was purified by silica gel chromatography.

## Supplementary material

Detailed facts of importance to specialist readers are published as ”Supporting Information”. Such documents are peer-reviewed, but not copy-edited or typeset. They are made available as submitted by the authors.
