# Supplementary material for: Mechanistic Studies on a Sulfoxide Transfer Reaction Mediated by Diphenyl Sulfoxide/Triflic Anhydride
Source: Chemistry. 2012 Jan 31;18(10):2987–97. doi: 10.1002/chem.201102861 (PMC3743163; doi:10.1002/chem.201102861)

CHEMISTRY
A EUROPEAN JOURNAL

Supporting Information

© Copyright Wiley-VCH Verlag GmbH & Co. KGaA, 69451 Weinheim, 2012

Mechanistic Studies on a Sulfoxide Transfer Reaction Mediated by Diphenyl Sulfoxide/Triflic Anhydride

Martin A. Fascione,[a] Sophie J. Adshead,[a] Pintu K. Mandal,[a] Colin A. Kilner,[a] Andrew G. Leach,[b] and W. Bruce Turnbull*[a]

chem_201102861_sm_miscellaneous_information.pdf

Supporting Information:

Studies on the mechanism of a sulfoxide transfer reaction mediated by diphenyl sulfoxide-triflic anhydride

Martin A. Fascione,[a] Sophie J. Adshead,[a] Pintu K. Mandal,[a] Colin A. Kilner,[a] Andrew G. Leach[b] and W. Bruce Turnbull[a]*

[a] School of Chemistry, University of Leeds, Leeds, LS2 9JT, UK; [b] AstraZeneca, Alderley Park, Macclesfield, Cheshire SK10 4TF, UK.

	**Page**
**General Experimental Methods**	**S2**
**ReactIR monitoring of the diphenyl sulfoxide – triflic anhydride reaction**	**S3**
**Identification and quantification of sulfonium ion 13 as a byproduct of the oxidation reaction**	**S6**
**Synthetic procedures**	**S8**
**Density Functional Theory Calculations**	**S22**
**References**	**S42**
**NMR Spectra**	**S43**

General Experimental Methods

All solvents were dried prior to use, according to standard methods.[1] Trifluoromethanesulfonic anhydride (Tf_2O) was distilled from phosphorus pentoxide under a N_2(g) atmosphere. Trimethylsilyl trifluoromethanesulfonate (TMSOTf) was freshly distilled under a N_2(g) atmosphere. Where appropriate anhydrous quality material was purchased. All solvents used for flash chromatography were GPR grade, except hexane and EtOAc, when HPLC grade was used. All concentrations were performed *in vacuo,* unless otherwise stated. All reactions were performed in oven dried glassware under a N_2(g) atmosphere, unless otherwise stated. ^{1}H NMR spectra were recorded at 500 MHz on a Bruker Avance 500 instrument or at 300 MHz on a Bruker Avance 300 instrument. ^{13}C NMR spectra were recorded at 75 MHz on a Bruker Avance 300 instrument. ^{19}F NMR spectra were recorded at 470 MHz on a Bruker Avance 500 instrument. Chemical shifts are given in parts per million downfield from tetramethylsilane. The following abbreviations are used in ^{1}H NMR analysis: s = singlet, d = doublet, t = triplet, q = quartet, m = multiplet, dd = double doublet, dt = double triplet, td = triple doublet, ddd = double double doublet. Electrospray (ES+) ionisation mass spectra were obtained on a Bruker HCT Ultra Ion Trap mass spectrometer connected to an Agilent 1200 series HPLC system equipped with a Phenomonex Luna C18(2) (5 micron, 50 mm x 2.0 mm) column. High resolution EI mass spectra were performed on a Waters GCT Premiere mass spectrometer and high resolution ES+ were performed on a Bruker Daltonics MicroTOF mass spectrometer. Isotopic ratios were calculated by comparison of the peak area of the most abundant ion adduct using Bruker Compass DataAnalysis 4.0 software. Infra-red spectra were recorded on a Perkin-Elmer Spectrum One FT-IR spectrometer. Analytical TLC was performed on silica gel 60-F^{254} (Merck) with detection by fluorescence and/or charring following immersion in Phosphomolybdic acid (PMA), or 5% H_2SO_4/MeOH solution for carbohydrates. Sulfoxides were detected on TLC plates using trifluoroacetic anhydride/sodium iodide according to the method of Drabowicz et al.[2]

ReactIR monitoring of the diphenyl sulfoxide – triflic anhydride reaction

A solution of diphenyl sulfoxide (244 mg, 1.21 mmol) in CH_2Cl_2 (2 mL) was cooled to −32 °C. An IR spectrum was recorded every 15 seconds using a ReactIR 45m (Mettler Toledo) equipped with a 1.5 m AgX fiber and a 9.5 mm DiComp (Diamond) probe. After acquiring baseline spectra for 10.5 mins, trifluoromethane sulfonic anhydride (0.20 mL, 1.21 mmol) was added in two aliquots within 45 seconds. The absorbance spectra (and corresponding second derivative spectra) were analyzed using the ConcIRT™ software package (Mettler Toledo). The authors thank Caroline Edwards, Andrew Smith and Jon Goode (Mettler Toledo AutoChem) for acquisition and analysis of the ReactIR data.

Figure S1 (following two pages). (a) 3-D plot of IR spectra as a function of time, highlighting the point at which triflic anhydride was added to the reaction mixture; (b) Overlay of IR spectra highlighting the spectral changes associated with the consumption of diphenyl sulfoxide and formation of reaction intermediates; (c) overlay of IR spectra following subtraction of the spectrum acquired at 9 min 25 sec (i.e., before addition of triflic anhydride); (d) second derivative of spectra displayed in part (c); (e) concentration profile of different species identified using the ConcIRT software; (f) comparison of spectra determined using ConcIRT software for component 1 (part e) overlaid with experimental reference spectra acquired for diphenyl sulfoxide and CH_2Cl_2; (g) concentration profile of different species identified from the second derivative data using the ConcIRT software (in this case diphenyl sulfoxide is component 2). Baseline changes that appear in the original spectra upon addition of triflic anhydride are removed in the second derivative mathematical treatment of the data; this difference may account for why fewer components are observed in the derivatised ConcIRTdata.

(a)

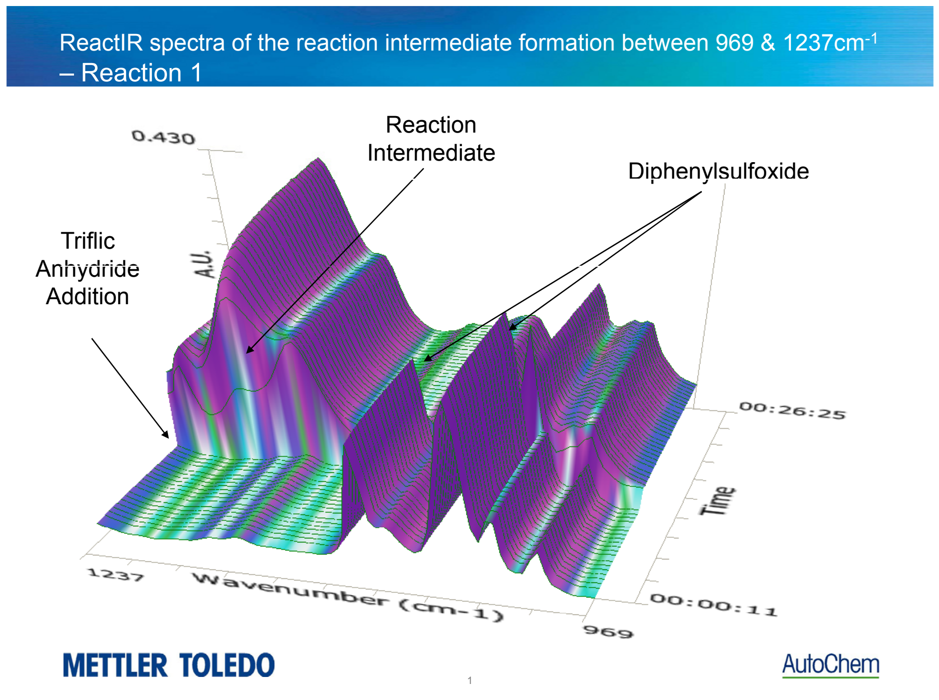
ReactIR spectra of the reaction intermediate formation between 969 & 1237cm-1 – Reaction 1
Reaction Intermediate
Diphenylsulfoxide
Triflic Anhydride Addition
0.430
A.U.
00:26:25
Time
00:00:11
1237
Wavenumber (cm-1)
969
METTLER TOLEDO
AutoChem
1

(b)

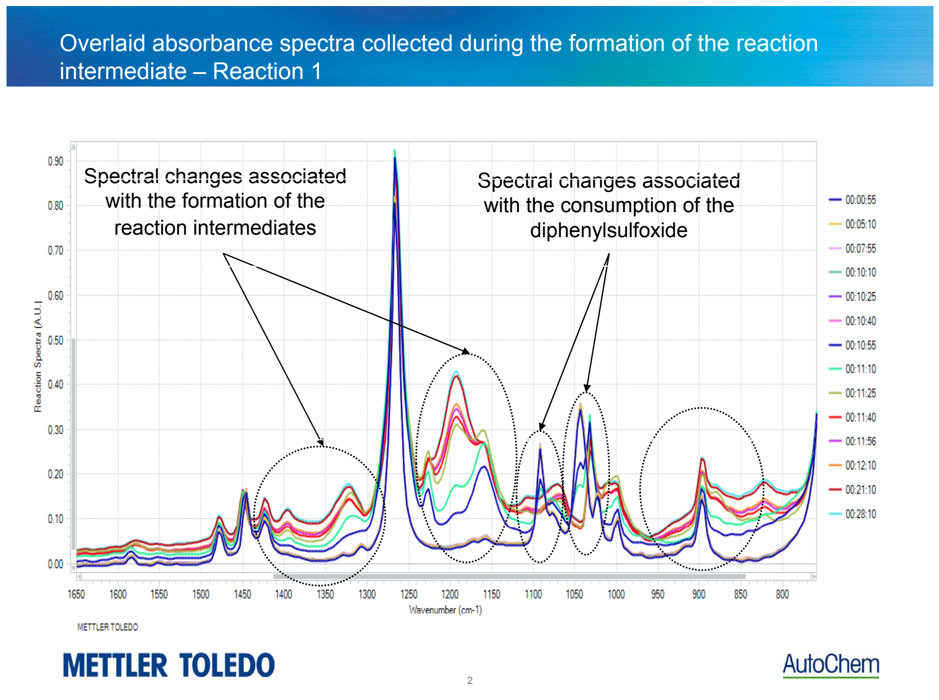
Overlaid absorbance spectra collected during the formation of the reaction intermediate – Reaction 1
Spectral changes associated with the formation of the reaction intermediates
Spectral changes associated with the consumption of the diphenylsulfoxide
Reaction Spectra (A.U.)
Wavenumber (cm-1)
00:00:55
00:05:10
00:07:55
00:10:10
00:10:25
00:10:40
00:10:55
00:11:10
00:11:25
00:11:40
00:11:56
00:12:10
00:21:10
00:28:10
METTLER TOLEDO
AutoChem
2

Figure S1 parts (a)-(d)

(c)

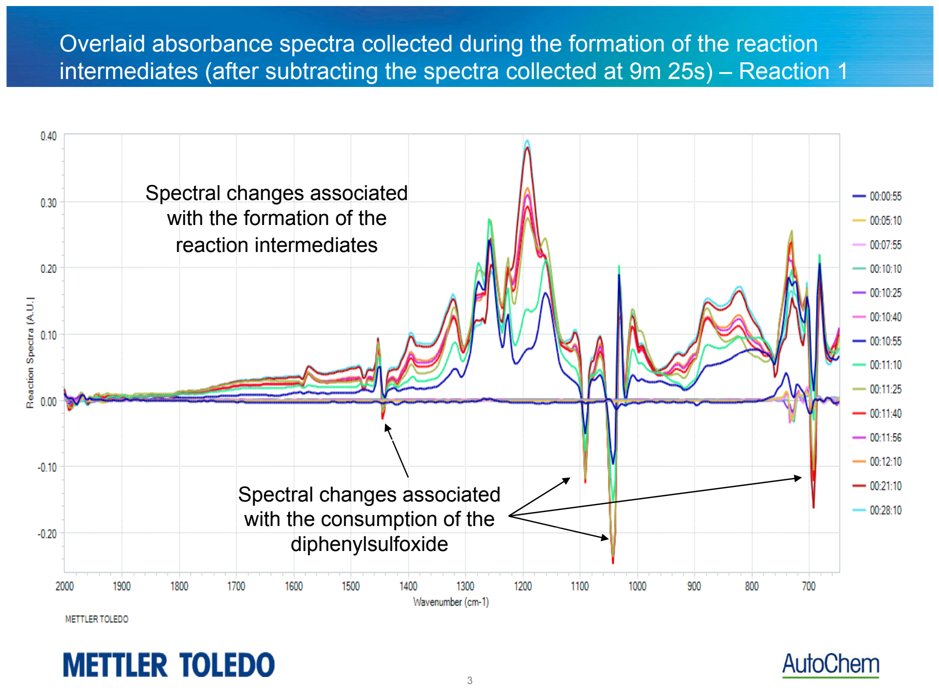
Overlaid absorbance spectra collected during the formation of the reaction intermediates (after subtracting the spectra collected at 9m 25s) – Reaction 1
Spectral changes associated with the formation of the reaction intermediates
Spectral changes associated with the consumption of the diphenylsulfoxide
Reaction Spectra (A.U.)
Wavenumber (cm-1)
00:00:55
00:05:10
00:07:55
00:10:10
00:10:25
00:10:40
00:10:55
00:11:10
00:11:25
00:11:40
00:11:56
00:12:10
00:21:10
00:28:10
METTLER TOLEDO
AutoChem
3

(d)

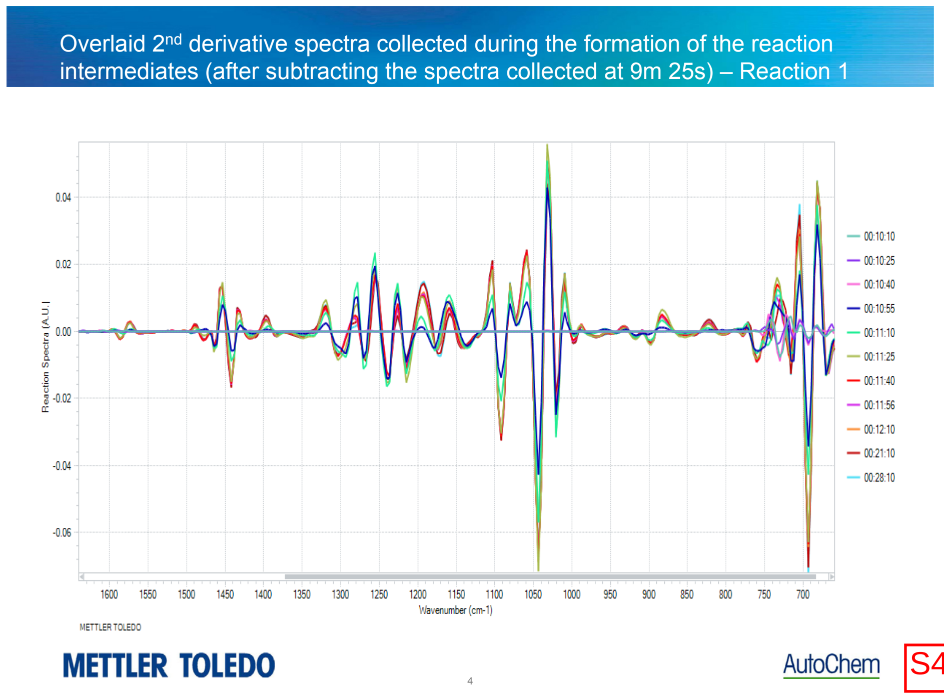
Overlaid 2nd derivative spectra collected during the formation of the reaction intermediates (after subtracting the spectra collected at 9m 25s) – Reaction 1
Reaction Spectra (A.U.)
Wavenumber (cm-1)
00:10:10
00:10:25
00:10:40
00:10:55
00:11:10
00:11:25
00:11:40
00:11:56
00:12:10
00:21:10
00:28:10
METTLER TOLEDO
AutoChem
4

(e)

ConcIRT analysis trends from absorbance spectra – Reaction 1

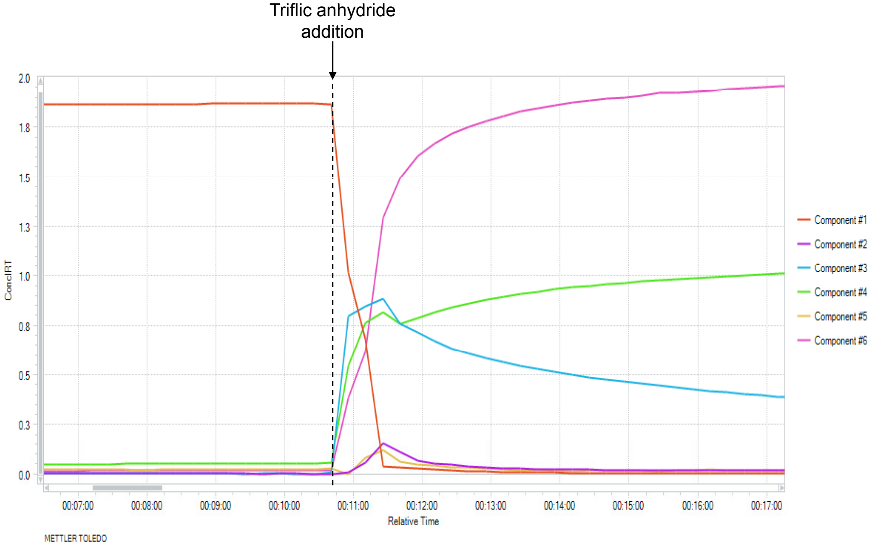

METTLER TOLEDO

5

AutoChem

(f)

ConcIRT Component 1 spectra vs reference spectra - Reaction 1

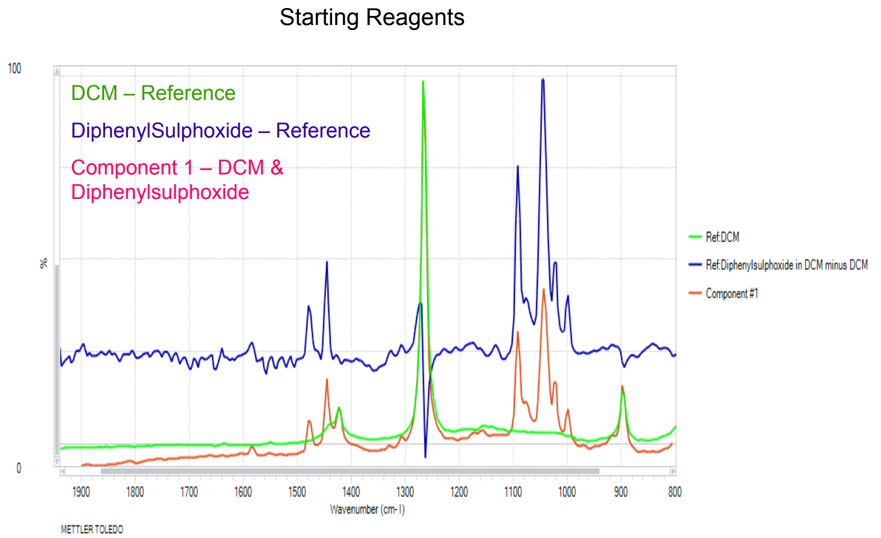

METTLER TOLEDO

6

AutoChem

(g)

ConcIRT analysis trends from 2nd derivative spectra – Reaction 1

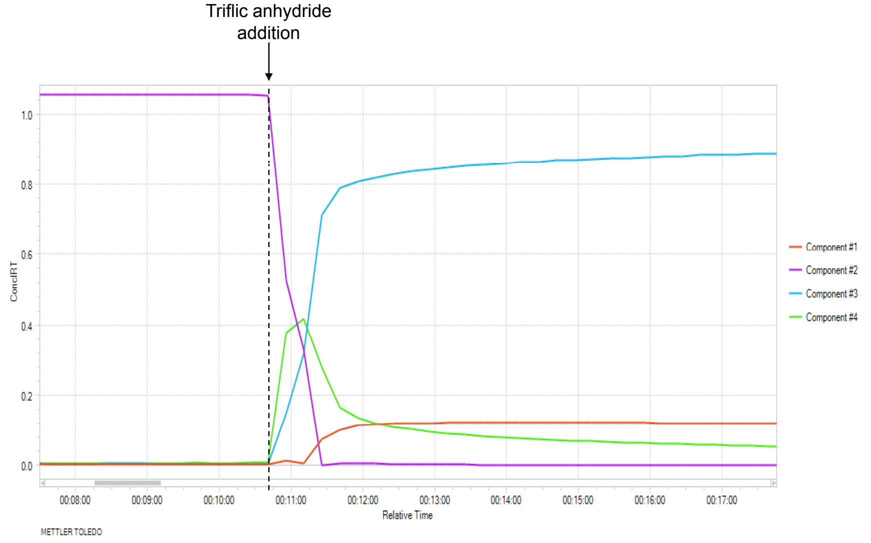

METTLER TOLEDO

7

AutoChem

Figure S1 parts (e)-(f)

Identification and quantification of sulfonium ion 13 as a byproduct of the oxidation reaction

The formation of (4-phenylthiophenyl)diphenylsulfonium triflate **13** was confirmed using LC-MS by comparison with an authentic sample (Sigma). A solution of 2-methoxy-2-(*S*)-phenyl-(3,4,6-tri-*O*-acetyl-1,2-dideoxy-β-D-glucopyranoso)[1,2-*e*]-1,4-oxathiane **1** (30 mg, 0.066 mmol), diphenyl sulfoxide (37 mg, 0.184 mmol) and DTBMP (041 mg, 0.198 mmol) in CH_2Cl_2 (0.3 mL) was cooled to −60 °C before addition of Tf_2O (16 μL, 0.092 mmol). After stirring for 1.5 h at this temperature, the mixture was diluted with CH_2Cl_2 (0.2 mL) and an aliquot (10 μL) was removed for analysis. The sample was diluted with MeOH (990 μL) and 5 x 5 μL aliquots of this solution were each diluted further with MeOH (995 μL). LC-MS analysis (Bruker HCT Ultra Ion Trap mass spectrometer connected to an Agilent 1200 series HPLC system equipped with a Phenomonex Luna C18(2) (5 micron, 50 mm x 2.0 mm) column) showed two major peaks in the ion chromatogram (Fig S2a). The peak eluting at 1.66 min had a mass of 371.1 Da and was identical to an authentic sample of (4-phenylthiophenyl)diphenylsulfonium triflate **13** (Fig S2b). The second peak eluting at 1.86 min had mass of 206.2 Da which corresponds to the protonated form of DTBMP.

Standard solutions of sulfonium ion **13** in MeOH were prepared with concentrations ranging from 0.52-4.14 μg/mL. Measurement of the total ion count for each the sulfonium ion sample was used to plot a standard curve (Fig S3) for comparison with the five samples from the reaction mixture, which had an average ion count of $9.45 \times 10^7 \pm 1.74 \times 10^7$. This value corresponds to a sulfonium ion concentration of 1.46 ± 0.22 μg/mL for the mass spec sample, and 29 ± 4 mg of sulfonium salt **13** in the original reaction mixture. The theoretical yield of compound **13** from this reaction was 34.4 mg.

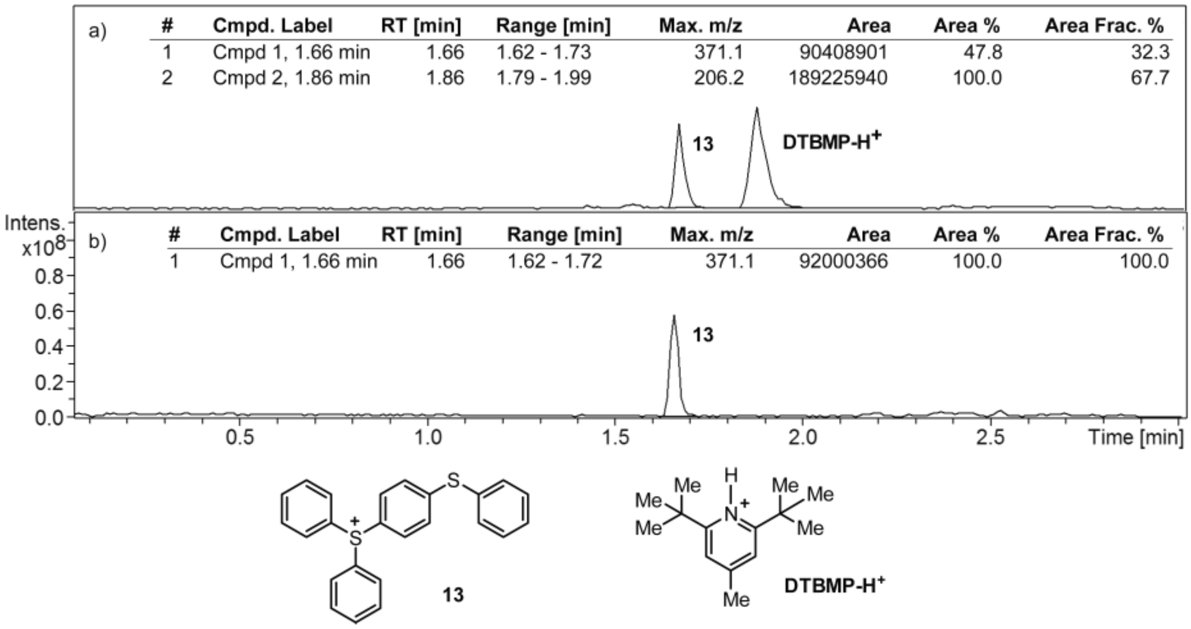

Fig. S2. Comparison of LC-MS ion count chromatograms for a) crude product mixture from oxidation of oxathiane **1** and b) an authentic sample of the triflate salt of sulfonium ion **13**. Neutral molecules e.g., the oxathiane-*S*-oxide product **2**, were also present, but have much lower intensities than the cationic byproducts **13** and protonated di-*t*-butylmethylpyridine **DTBMP-H**$^+$.

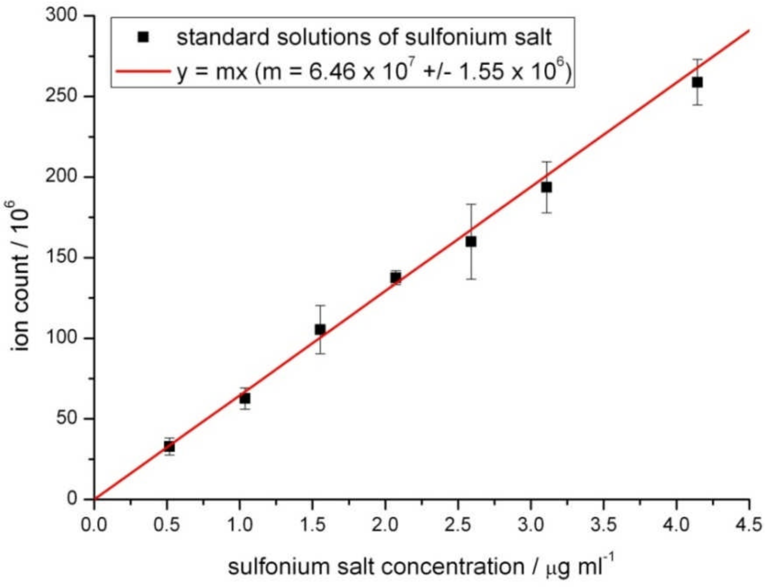

Fig. S3. Standard curve of ion count vs concentration of sulfonium triflate salt **13**. Each data point is the average of three measurements, with error bars corresponding to the standard deviation of these measurements.

2-Methoxy-2-(*S*)-phenyl-(3,4,6-tri-*O*-acetyl-1,2-dideoxy-β-D-glucopyranoso)[1,2-*e*]-1,4-oxathiane-*S*-oxide (2)

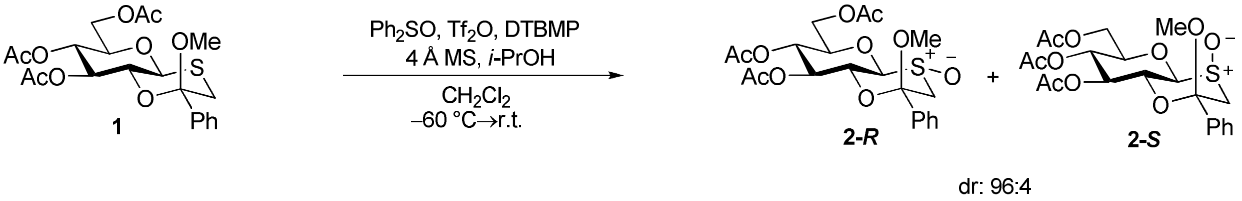

Tf_2O (52 µL, 0.308 mmol) was added to a solution of 2-methoxy-2-(*S*)-phenyl-(3,4,6-tri-*O*-acetyl-1,2-dideoxy-β-D-glucopyranoso)[1,2-*e*]-1,4-oxathiane **1** (100 mg, 0.220 mmol), diphenyl sulfoxide (124 mg, 0.617 mmol), DTBMP (135 mg, 0.661 mmol) and 4 Å mol. sieves (50 mg) in CH_2Cl_2 (1 mL) at −60 °C. The reaction mixture was stirred for 5 min at −60 °C, then isopropanol (25 µL, 0.33 mmol) was added, and the reaction mixture gradually raised to RT over 75 min. The mixture was then quenched with aq. $NaHCO_3$ (2 mL), diluted with CH_2Cl_2 (5 mL), washed with aq. NaCl (2 x 5 mL), dried ($MgSO_4$) and concentrated to leave a crude colourless solid. The crude solid was purified by flash column chromatography (silica; 2:1 (v/v) hexane-EtOAc→1:1 (v/v) hexane-EtOAc) to afford **2** (74 mg, 72%, dr: 96:4) as colourless needles, m.p. 181.3-185.4 ºC (equatorial diastereomer, from 1:1 (v/v) hexane-EtOAc); R_f 0.56 (2:1 (v/v) EtOAc-hexane); analytical data for compound **2** were identical to those reported previously.[3]

2-(*S*)-Phenyl-(3,4,6-tri-*O*-acetyl-1,2-dideoxy-β-D-glucopyranoso)[1,2-*e*]-1,4-oxathiane-*S*-oxide (4)

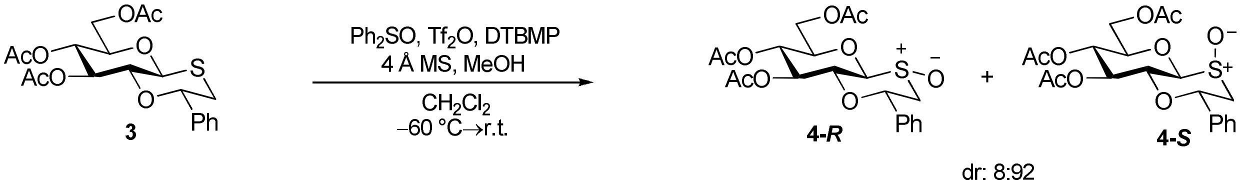

Tf_2O (28 µL, 0.165 mmol) was added to a solution of 2-(*S*)-phenyl-(3,4,6-tri-*O*-acetyl-1,2-dideoxy-β-D-glucopyranoso)[1,2-*e*]-1,4-oxathiane **3** (50 mg, 0.118 mmol), diphenyl sulfoxide (67 mg, 0.330 mmol), DTBMP (72 mg, 0.354 mmol) and 4 Å mol. sieves (25 mg) in CH_2Cl_2 (500 µL) at −60 °C. The reaction mixture was stirred for 5 min at −60 °C, then MeOH (7 µL, 0.18 mmol) was added, and the reaction mixture was gradually raised to RT over 90 min. The mixture was then quenched with aq. $NaHCO_3$ (1 mL), diluted with CH_2Cl_2 (5 mL), washed with aq. NaCl (2 x 10 mL) and concentrated to leave a crude colourless solid. The crude solid was purified by flash column chromatography (silica; 1:1 (v/v) hexane-EtOAc) to afford **4** (40 mg, 78%, dr: 8:92) as a colourless glassy solid; R_f 0.13 (1:1 (v/v) hexane-EtOAc); analytical data for compound **4** were identical to those reported previously.[4]

^{18}O-Labelled diphenyl sulfoxide [^{18}O]-5

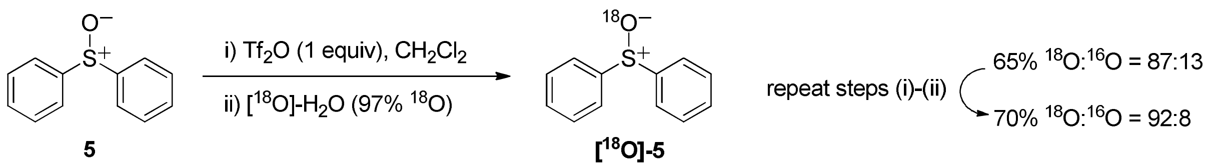

Tf_2O (209 µL, 1.24 mmol) was added to a solution of diphenyl sulfoxide **5** (250 mg, 1.24 mmol) in CH_2Cl_2 (4.8 mL) at −60 ºC in a cardice bath. The reaction mixture was held at −60 ºC for 10 min, and then H_2O (97% ^{18}O, 27 µL, 1.49 mmol) was added. The reaction mixture was then instantly removed from the cardice bath and then stirred for 30 min, before quenching with triethylamine (378 µL, 2.72 mmol), diluted with CH_2Cl_2 (10 mL), and washed with 1M HCl (3 x 10 mL), aq. $NaHCO_3$ (3 x 10 mL), aq. NaCl (3 x 10 mL), dried ($MgSO_4$) and concentrated to afford a crude solid. The crude solid was purified by flash column chromatography (silica; 2:1 (v/v) hexane-EtOAc) to afford **[^{18}O]-5** (161 mg, 64%, 87% ^{18}O incorporation) as a crystalline colourless solid. The product (50 mg, 0.248 mmol) was redissolved in CH_2Cl_2 (960 µL) at −60 ºC in a cardice bath, and Tf_2O (42 µL, 0.248 mmol) was added to the solution. The reaction mixture was held at −60 ºC for 10 min, and then H_2O (97% ^{18}O, 5.35 µL, 0.297 mmol) was added. The reaction mixture was then instantly removed from the cardice bath and then stirred for 30 min, before quenching with triethylamine (76 µL, 0.544 mmol), diluted with CH_2Cl_2 (5 mL), and washed with 1M HCl (3 x 5 mL), aq. $NaHCO_3$ (3 x 5 mL), aq. NaCl (3 x 5 mL), dried ($MgSO_4$) and concentrated to afford a crude solid. The crude solid was purified by flash column chromatography (silica; 2:1 (v/v) hexane-EtOAc) to afford **[^{18}O]-5** (35 mg, 70%, 92% ^{18}O incorporation) as a crystalline colourless solid. Analytical data were identical to those reported previously.[5]

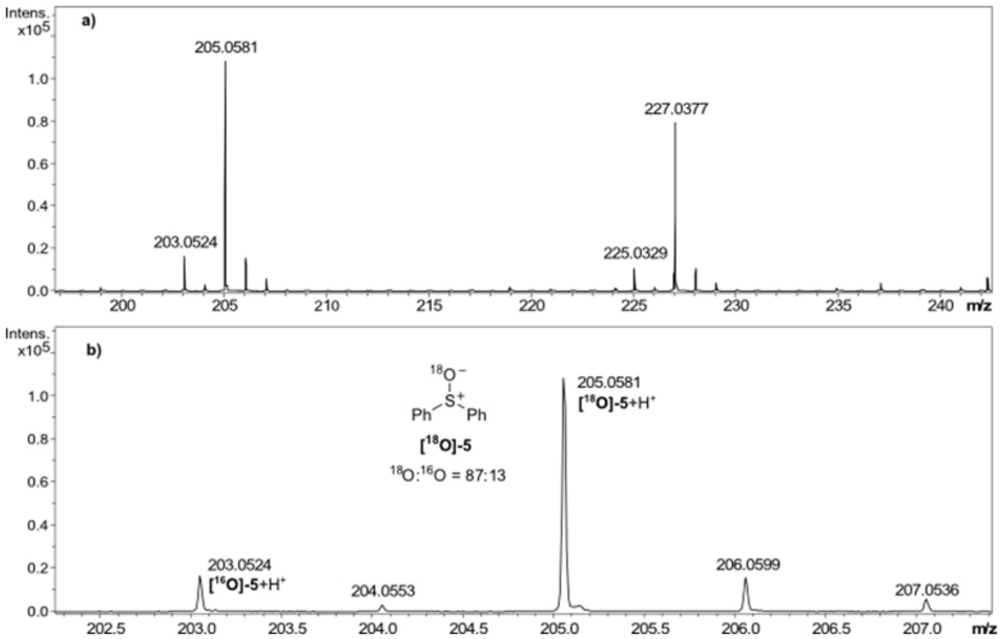

Fig. S4. a) Mass spectrum and b) expansion of **[^{18}O]-5** (87% ^{18}O incorporation).

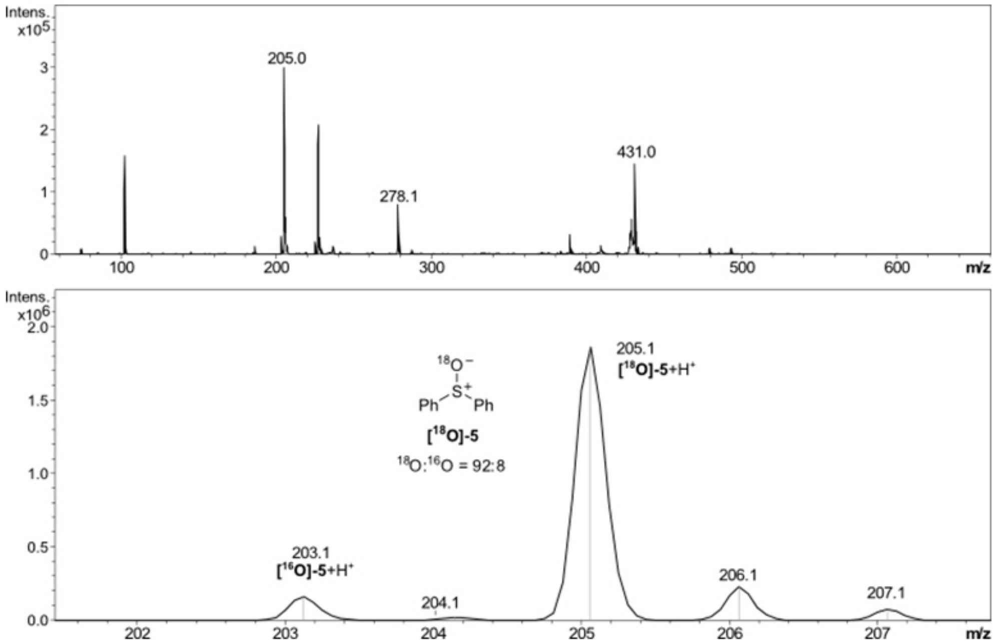

Fig. S5. a) Overall mass spectrum and b) expansion of **[18O]-5** (92% ^{18}O incorporation).

Reaction of [18O]-5 (92% ^{18}O), [16O]-15 and Tf_2O (1:1:1 mixture)

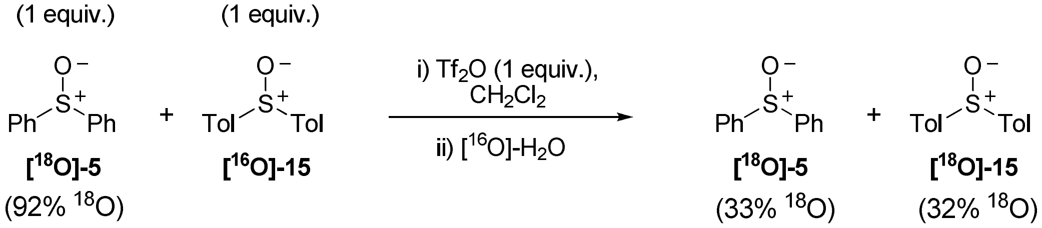

Tf_2O (20.7 µL, 0.123 mmol) was added to a solution of diphenyl sulfoxide **[18O]-5 (92% ^{18}O)** (25 mg, 0.123 mmol) and ditolyl sulfoxide **[16O]-15** (28 mg, 0.123 mmol) in CH_2Cl_2 (980 µL) at −60 ºC in a cardice bath. The reaction mixture was held at −60 ºC for 10 min, and then H_2O (2.7 µL, 0.147 mmol) was added, followed by triethylamine (37.4 µL, 0.270 mmol). The reaction mixture was then instantly removed from the cardice bath and diluted with CH_2Cl_2 (10 mL), and washed with 1M HCl (10 mL), aq. $NaHCO_3$ (10 mL), aq. NaCl (10 mL), dried ($MgSO_4$) and concentrated to afford a crude mixture, which was analysed by HR-ESIMS.

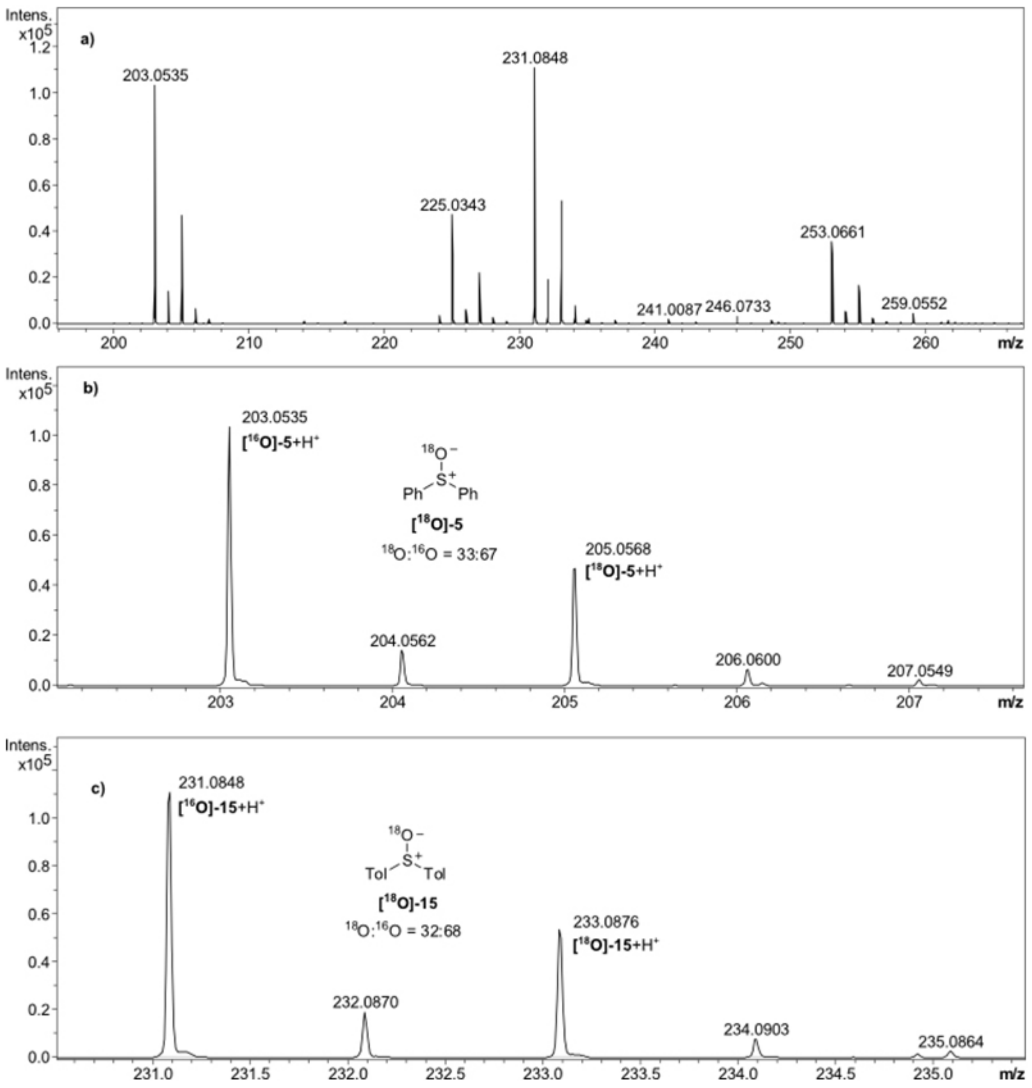

Fig. S6. a) Overall mass spectrum and expansions highlighting b) **[^{18}O]-5** (33% ^{18}O incorporation) and c) **[^{18}O]-15** (32% ^{18}O incorporation).

Reaction of [^{18}O]-5 (92% ^{18}O), [^{16}O]-15 and Tf_2O (1:1:2 mixture)

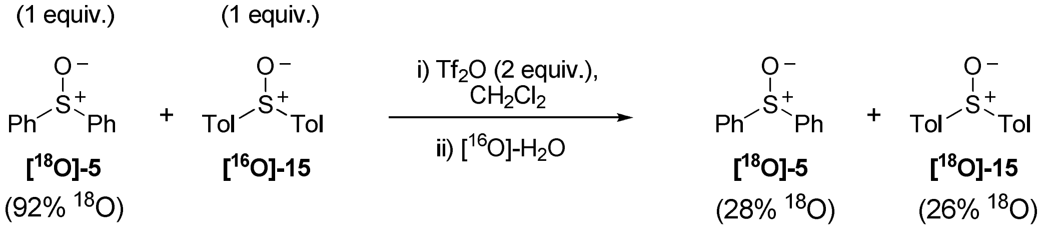

Tf_2O (33 µL, 0.196 mmol) was added to a solution of diphenyl sulfoxide **[^{18}O]-5 (92% ^{18}O)** (20 mg, 0.098 mmol) and ditolyl sulfoxide **[^{16}O]-15** (22.6 mg, 0.098 mmol) in CH_2Cl_2 (795 µL) at −60 ºC in a cardice bath. The reaction mixture was held at −60 ºC for 10 min, and then H_2O (4.3 µL, 0.235 mmol) was added, followed by triethylamine (60 µL, 0.431 mmol). The reaction mixture was then instantly removed from the cardice bath and diluted with CH_2Cl_2 (10 mL), and washed with 1M HCl (10 mL), aq. $NaHCO_3$ (10 mL), aq. NaCl (10 mL), dried ($MgSO_4$) and concentrated to afford a crude mixture, which was analysed by HR-ESIMS.

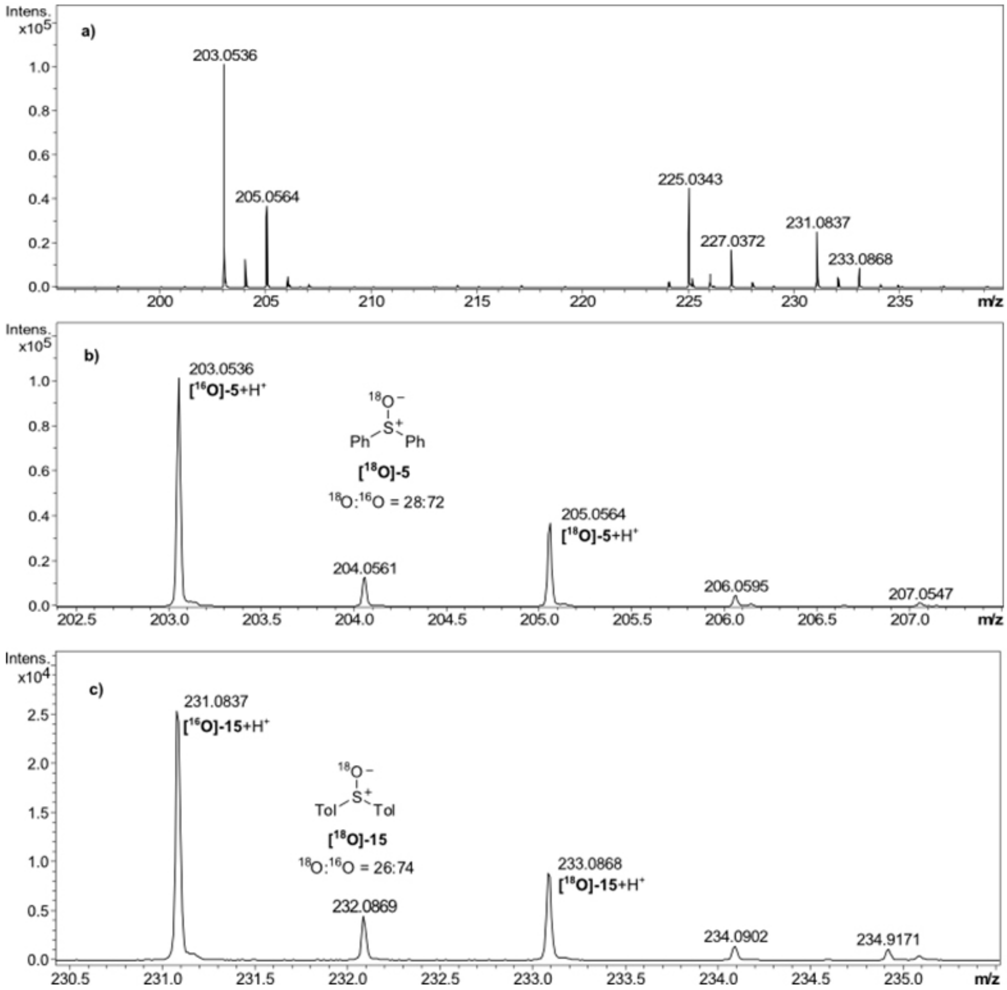

Fig. S7. a) Overall mass spectrum and expansions of b) **[^{18}O]-5** (28% ^{18}O incorporation) and c) **[^{18}O]-15** (26% ^{18}O incorporation).

2-Methoxy-2-(*S*)-phenyl-(3,4,6-tri-*O*-acetyl-1,2-dideoxy-β-D-glucopyranoso)[1,2-*e*]-1,4-oxathiane *S*-[^{18}O]-oxide (2)

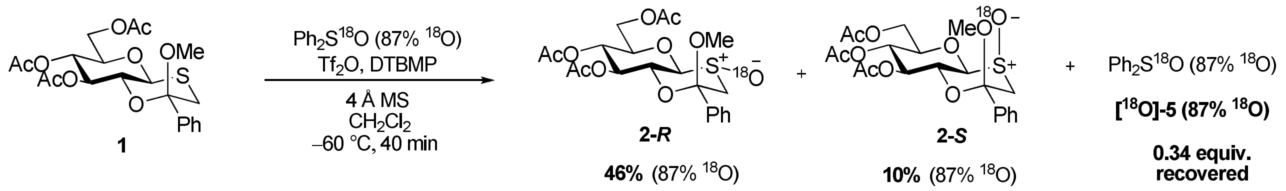

Tf_2O (26 μL, 0.154 mmol) was added to a solution of 2-methoxy-2-(*S*)-phenyl-(3,4,6-tri-*O*-acetyl-1,2-dideoxy-β-D-glucopyranoso)[1,2-*e*]-1,4-oxathiane **1** (50 mg, 0.110 mmol), diphenyl sulfoxide **[^{18}O]-5 (87% ^{18}O)** (63 mg, 0.308 mmol), DTBMP (68 mg, 0.330 mmol) and 4 Å mol. sieves (25 mg) in CH_2Cl_2 (500 μL) at −60 °C. The reaction mixture was stirred for 40 min at −60 °C then gradually raised to −30 °C over 30 min. The mixture was then quenched with aq. $NaHCO_3$ (1 mL), diluted with CH_2Cl_2 (5 mL), washed with aq. NaCl (2 x 5 mL), dried ($MgSO_4$) and concentrated to leave a crude colourless solid. The crude solid was purified by flash column chromatography (silica; 4:1 (v/v) hexane-EtOAc→3:2 (v/v) hexane-EtOAc) to afford **2-*R*** (24 mg, 46%, 87% ^{18}O incorporation) as the major product as colourless needles, m.p. 181.3-185.4 ºC (from 1:1 (v/v) hexane-EtOAc); R_f 0.56 (2:1 (v/v) EtOAc-hexane); Found $[M+NH_4]^+$ 490.1585, $C_{21}H_{30}NO_9{}^{18}OS$ requires 490.1627; other analytical data for compound **2-*R*** were identical to those reported previously.[3]

2-*S* (5 mg, 10%, 87% ^{18}O incorporation) was isolated as the minor product as colourless needles; m.p. 185.7-188.2 ºC (from 1:1 (v/v) hexane-EtOAc). R_f 0.38 (2:1 (v/v) EtOAc-hexane); Found $[M+NH_4]^+$ 490.1593, $C_{21}H_{30}NO_9{}^{18}OS$ requires 490.1627; other analytical data for compound **2-*S*** were identical to those reported previously.[3]

2-(*S*)-Phenyl-(3,4,6-tri-*O*-acetyl-1,2-dideoxy-β-D-glucopyranoso)[1,2-*e*]-1,4-oxathiane (*S*)-*S*-[^{18}O]-oxide (4-*S*)

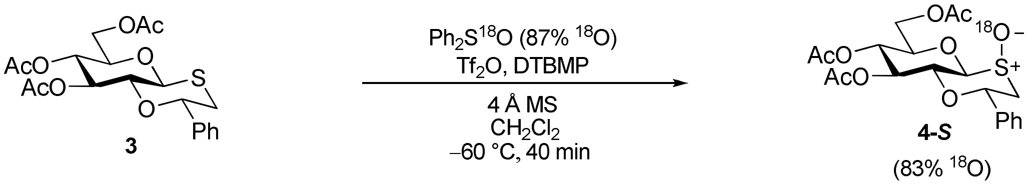

Tf_2O (14 µL, 83 µmol) was added to a solution of 2-(*S*)-phenyl-(3,4,6-tri-*O*-acetyl-1,2-dideoxy-β-D-glucopyranoso)[1,2-*e*]-1,4-oxathiane **3** (25 mg, 59 µmol), diphenyl sulfoxide **[^{18}O]-5 (87% ^{18}O)** (34 mg, 0.165 mmol), DTBMP (36 mg, 0.176 mmol) and 4 Å mol. sieves (12 mg) in CH_2Cl_2 (270 µL) at −60 °C. The reaction mixture was stirred for 40 min at −60 °C then gradually raised to −30 °C over 30 min. The mixture was then quenched with aq. $NaHCO_3$ (1 mL), diluted with CH_2Cl_2 (5 mL), washed with aq. NaCl (2 x 5 mL), dried ($MgSO_4$) and concentrated to leave a crude colourless solid. The crude solid was purified by flash column chromatography (silica; 1:1 (v/v) hexane-EtOAc) to afford **4-*S*** (18 mg, 69%, 83% ^{18}O incorporation) as a colourless glassy solid; R_f 0.13 (1:1 (v/v) EtOAc-hexane); Found $[M+NH_4]^+$ 460.1498, $C_{20}H_{28}NO_8{}^{18}OS$ requires 460.1522; other analytical data for compound **4-*S*** were identical to those reported previously.[4]

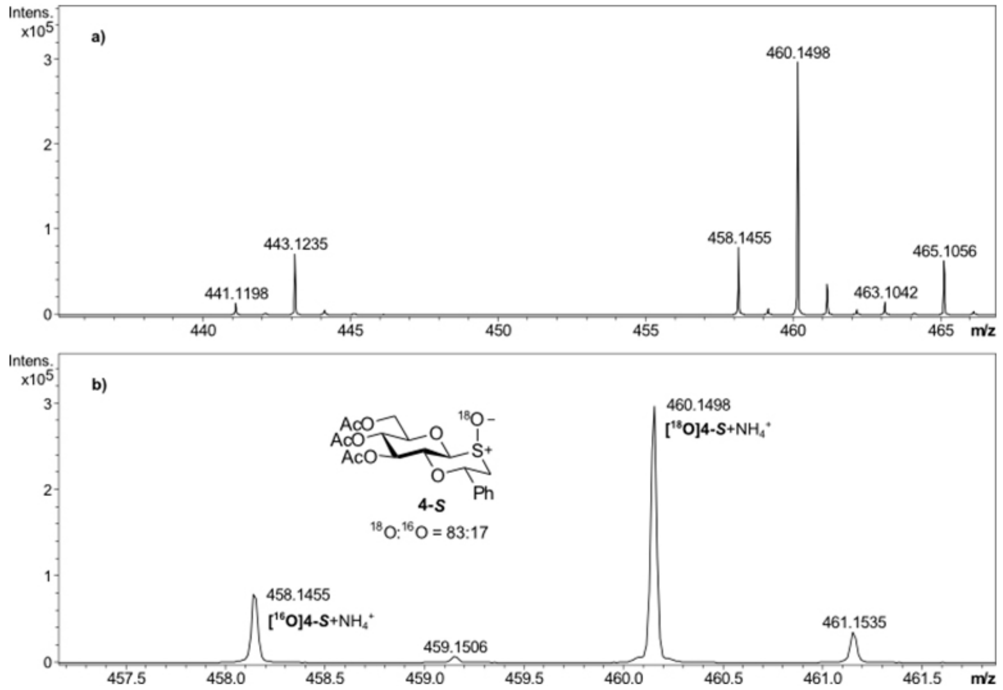

Fig. S8. a) Mass spectrum and expansion of b) **[^{18}O]-4-*S*** (83% ^{18}O incorporation).

Activation of sulfoxide 2-*R* with trifluoromethanesulfonic anhydride and quenching of the reaction mixture with diphenyl sulfide

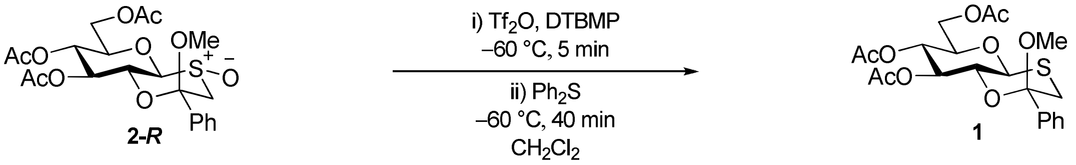

Tf_2O (20 μL, 0.117 mmol) was added to a solution of 2-methoxy-2-(*S*)-phenyl-(3,4,6-tri-*O*-acetyl-1,2-dideoxy-β-D-glucopyranoso)[1,2-*e*]-1,4-oxathiane4-*R*)-*S*-oxide **2-*R*** (50 mg, 0.106 mmol) and DTBMP (66 mg, 0.319 mmol) in CH_2Cl_2 (490 μL) at −60 °C. After 5 min diphenyl sulfide **26** (35 μL, 0.213 mmol) was added and the reaction mixture was stirred at −60 °C for 40 min and then warmed to RT over 15 min. The mixture was then quenched with aq. $NaHCO_3$ (1 mL), diluted with CH_2Cl_2 (5 mL), washed with aq. NaCl (2 x 5 mL), dried ($MgSO_4$) and concentrated to leave a crude colourless solid. The crude solid was purified by flash column chromatography (silica; 3:1 (v/v) hexane-EtOAc) to afford 2-methoxy-2-(*S*)-phenyl-(3,4,6-tri-*O*-acetyl-1,2-dideoxy-β-D-glucopyranoso)[1,2-*e*]-1,4-oxathiane **1** (30 mg, 62%) as colourless needles, m.p. 164.2-166.8 ºC (from MeOH); R_f 0.35 (3:1 (v/v) hexane-EtOAc); analytical data for compound **1** were identical to those reported previously.[3]

Reaction of ^{18}O-enriched diphenyl sulfoxide [^{18}O]-5 with 2-methoxy-2-(*S*)-phenyl-(3,4,6-tri-*O*-acetyl-1,2-dideoxy-β-D-glucopyranoso)[1,2-*e*]-1,4-oxathiane-4-(*R*)-*S*-oxide 2-*R* in the presence of trifluoromethanesulfonic anhydride.

(a) Pre-activation of diphenyl sulfoxide with trifluoromethanesulfonic anhydride.

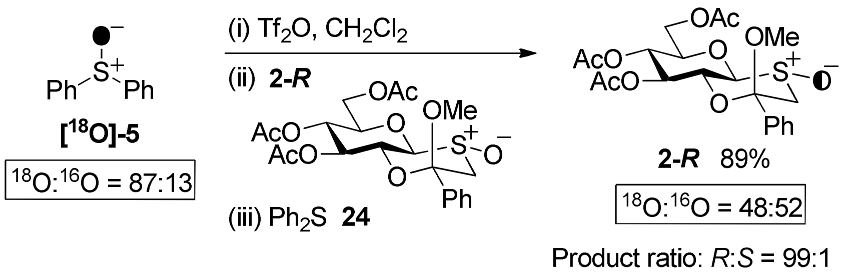

Tf_2O (23 μL, 134 μmol) was added to a solution of diphenyl sulfoxide (87% ^{18}O, 55 mg, 268 μmol) and DTBMP (59 mg, 287 μmol) in CH_2Cl_2 (390 μL) at −60 °C. After 10 min, 2-methoxy-2-(*S*)-phenyl-(3,4,6-tri-*O*-acetyl-1,2-dideoxy-β-D-glucopyranoso)[1,2-*e*]-1,4-oxathiane-4-(*R*)-*S*-oxide **2-*R*** (45 mg, 96 μmol) was added and the reaction mixture was stirred at −60 °C for 40 min. Diphenyl sulfide (22 μL, 134 μmol) was then added and the reaction mixture was warmed to RT over 10 min. The mixture was then quenched with aq. $NaHCO_3$ (1 mL), diluted with CH_2Cl_2 (5 mL), washed

with aq. NaCl (2 x 5 mL), dried ($MgSO_4$) and concentrated to leave a colourless oil. The crude solid was purified by flash column chromatography (silica; 3:2 (v/v) hexane-EtOAc) to afford **2-*R*** (40 mg, 89%, 48% ^{18}O incorporation) as the major product as colourless needles, m.p. 181.3-185.4 ºC (from 1:1 (v/v) hexane-EtOAc); R_f 0.56 (2:1 (v/v) EtOAc-hexane); analytical data for compound **2-*R*** were identical to those reported previously,[3] and recovered diphenyl sulfoxide **[^{18}O]-5** (26 mg, 1.34 equiv. recovered, 44% ^{18}O incorporation).

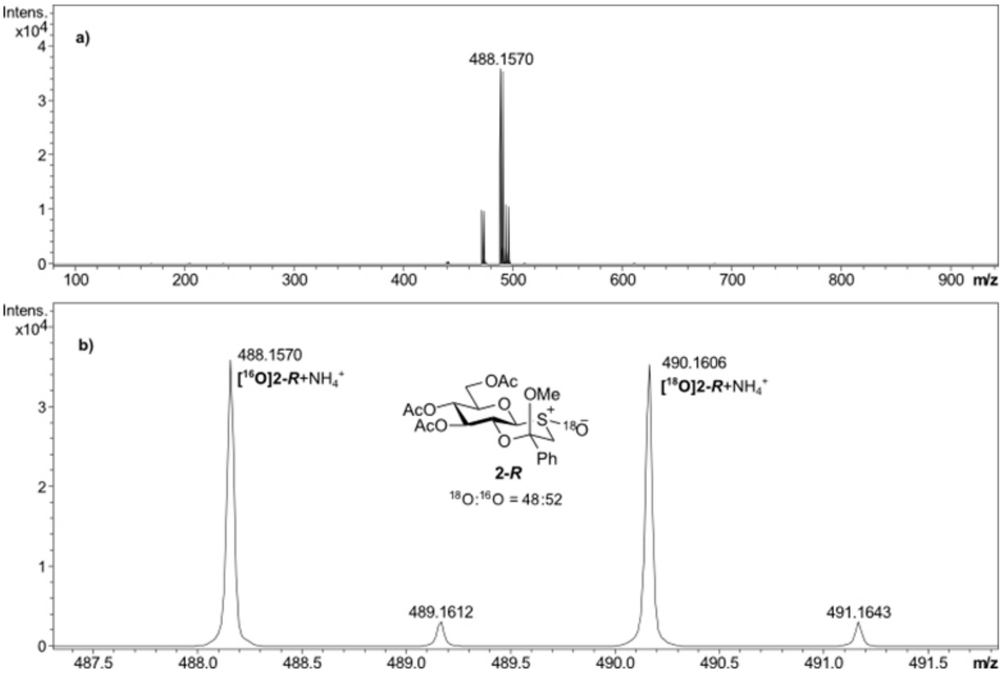

Fig. S9. a) Overall mass spectrum and expansion of b) **[^{18}O]-2-*R*** (48% ^{18}O incorporation).

(b) Pre-activation of oxathiane 2-*R* with trifluoromethanesulfonic anhydride.

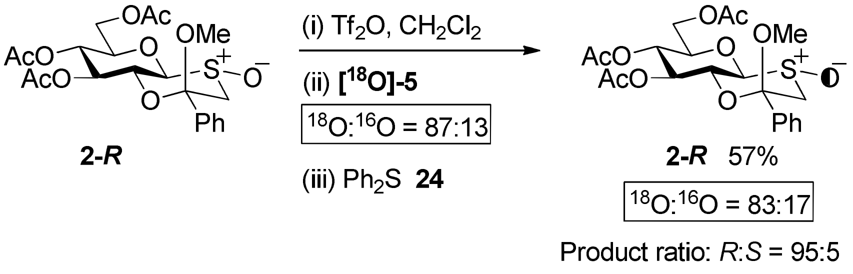

Tf_2O (14 µL, 82 µmol) was added to a solution of 2-methoxy-2-(*S*)-phenyl-(3,4,6-tri-*O*-acetyl-1,2-dideoxy-β-D-glucopyranoso)[1,2-*e*]-1,4-oxathiane-4-(*R*)-*S*-oxide **2-*R*** (35 mg, 74 µmol) and DTBMP (46 mg, 0.223 mmol) in CH_2Cl_2 (280 µL) at −60 °C. After 5 min, a solution of diphenyl sulfoxide (87% ^{18}O, 34 mg, 0.163 mmol) in CH_2Cl_2 (60 µL) was added and the reaction mixture was stirred at −60 °C for 40 min. Diphenyl sulfide (14 µL, 82 µmol) was then added and the reaction mixture was warmed to RT over 10 min. The mixture was then quenched with aq. $NaHCO_3$ (1 mL), diluted with CH_2Cl_2 (5 mL), washed with aq. NaCl (2 x 5 mL), dried ($MgSO_4$) and

concentrated to leave a crude colourless solid. The crude solid was purified by flash column chromatography (silica; 3:2 (v/v) hexane-EtOAc) to afford **2-*R*** (20 mg, 57%, 83% ^{18}O incorporation) as the major product as colourless needles, m.p. 181.3-185.4 ºC (from 1:1 (v/v) hexane-EtOAc); R_f 0.56 (2:1 (v/v) EtOAc-hexane); analytical data for compound **2-*R*** were identical to those reported previously.[3]

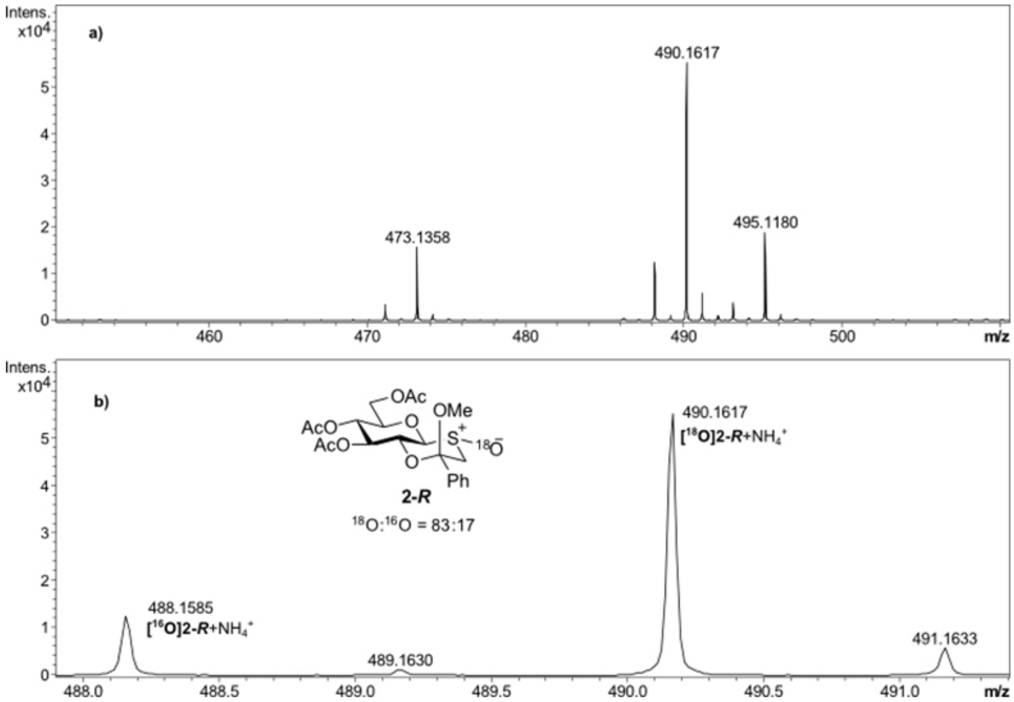

Fig. S10. a) Overall mass spectrum and expansion of b) **[^{18}O]-2-*R*** (83% ^{18}O incorporation).

Reaction of ^{18}O-enriched diphenyl sulfoxide [^{18}O]-5 with 2-(*S*)-Phenyl-(3,4,6-tri-*O*-acetyl-1,2-dideoxy-β-D-glucopyranoso)[1,2-*e*]-1,4-oxathiane-*S*-oxide 4-*S* in the presence of trifluoromethanesulfonic anhydride.

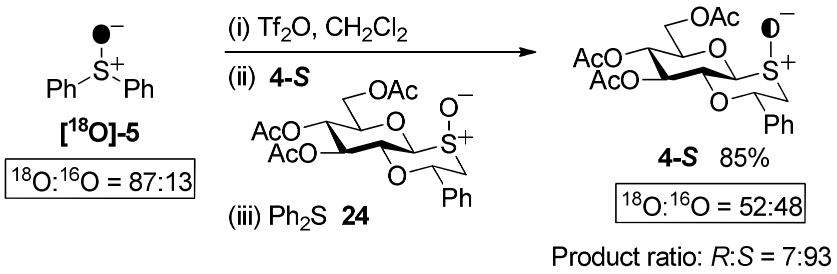

Tf_2O (14 µL, 83 µmol) was added to a solution of diphenyl sulfoxide (87% ^{18}O, 34 mg, 165 µmol) and DTBMP (36 mg, 177 µmol) in CH_2Cl_2 (200 µL) at −60 °C. After 10 min, 2-(*S*)-phenyl-(3,4,6-tri-*O*-acetyl-1,2-dideoxy-β-D-glucopyranoso)[1,2-*e*]-1,4-oxathiane-4-(*S*)-*S*-oxide **4-*S*** (26 mg, 59 µmol) was added and the reaction mixture was stirred at −60 °C for 40 min. Diphenyl sulfide (15 µL, 83 µmol) was then added and the reaction mixture was warmed to RT over 10 min. The mixture

was then quenched with aq. $NaHCO_3$ (1 mL), diluted with CH_2Cl_2 (5 mL), washed with aq. NaCl (2 x 5 mL), dried ($MgSO_4$) and concentrated to leave a colourless solid. The crude solid was purified by flash column chromatography (silica; 1:1 (v/v) hexane-EtOAc) to afford **4-*S*** (22 mg, 85%, 52% ^{18}O incorporation) as the major product as a colourless glassy solid (from 1:1 (v/v) hexane-EtOAc); R_f 0.13 (1:1 (v/v) EtOAc-hexane); analytical data for compound **4-*S*** were identical to those reported previously,[4] and recovered diphenyl sulfoxide **[^{18}O]-5** (14 mg, 1.17 equiv. recovered, 52% ^{18}O incorporation).

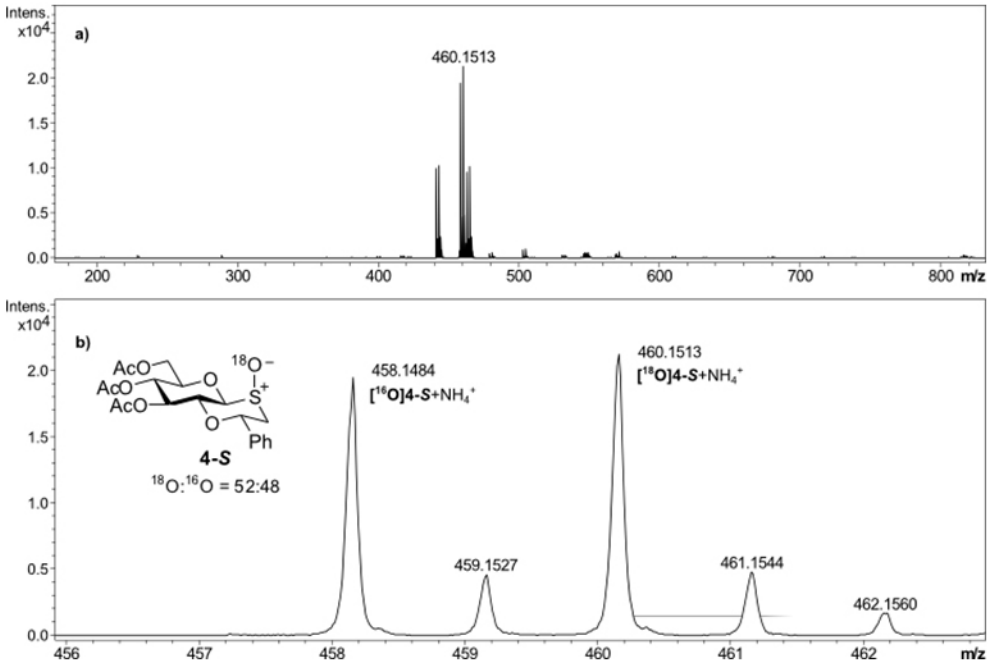

Fig. S11. a) Overall mass spectrum and expansion of b) **[^{18}O]-4-*S*** (52% ^{18}O incorporation).

2-[(2-Hydroxyethyl)sulfanyl]-1-phenylethan-1-one (31)

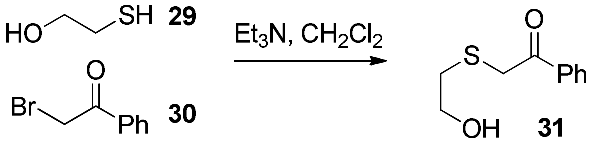

Bromoacetophenone **30** (500 mg, 2.51 mmol) was added to a solution of β-mercaptoethanol **29** (196 mg, 2.51 mmol) and triethylamine (280 mg, 2.76 mmol) in CH_2Cl_2 (8 mL) and the mixture was stirred at RT for 30 min. The mixture was diluted with CH_2Cl_2 (50 mL), washed with water, dried ($MgSO_4$) and concentrated to give ketone **31** as a colourless oil (477 mg, 97%) which was used in the next step without further purification. R_f=0.17 (hexane/EtOAc 2:1); ^1H NMR (500 MHz, $CDCl_3$, 298 K): δ=7.98 (d, ^3J(H,H)=7.5 Hz, 2H; Ar-H), 7.60 (t, ^3J(H,H)=7.5 Hz, 1H; Ar-H), 7.49 (d, ^3J(H,H)=7.5 Hz, 2H; Ar-H), 3.88 (s, 2H; SCH_2C(O)), 3.77 (q, ^3J(H,H)=5.8 Hz, 2H; CH_2OH), 2.79 (t, ^3J(H,H)=5.8 Hz, 2H; CH_2CH_2S), 2.48 ppm (t, ^3J(H,H)=5.8 Hz, 1H; OH); ^{13}C NMR (75 MHz,

$CDCl_3$, 300 K): δ=195.1 (C=O), 135.2 (Ar-C), 133.8 (Ar-C), 128.9 (Ar-C), 128.9 (Ar-C), 60.7 (CH_2OH), 37.1 (SCH_2C(O)), 35.7 ppm (CH_2CH_2S); IR (thin film): ν=3393 (O-H), 1672 cm^{-1} (C=O); HRMS (ESI): m/z calcd for $C_{10}H_{12}O_2S+Na^+$: 219.0456 [M+Na^+]; found: 219.0466.

2-Phenyl-1,4-oxathiane (rac-32)

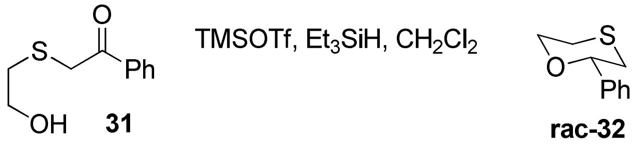

A solution of 2-[(2-hydroxyethyl)sulfanyl]-1-phenylethan-1-one **31** in CH_2Cl_2 (5 mL) was cooled to 0 °C before dropwise addition of trimethylsilyl trifluoromethanesulfonate (765 mg, 3.44 mmol). Triethylsilane (401 mg, 3.44 mmol) was added dropwise to this mixture. After stirring for 90 min at 0 °C, the reaction was quenched by addition of MeOH (5 mL). The mixture was neutralised with triethylamine and concentrated to a crude oil. The product was purified by flash column chromatography (silica; hexane, then 3:1 (v/v) hexane-EtOAc) to afford **rac-32** (288 mg, 70%) as a colourless oil. R_f=0.17 (hexane/EtOAc 2:1); ^{1}H NMR (500 MHz, $CDCl_3$, 298 K): δ=7.37-7.27 (m, 5H; Ar-H), 4.63 (dd, $^3J(H_{2ax},H_{3ax})$=10.6 Hz, $^3J(H_{2ax},H_{3eq})$=2.1 Hz, 1H; H-2ax), 4.39 (dt, $^3J(H_{6ax},H_{6eq})$=12.0 Hz, $^3J(H_{6eq},H_{5ax})$=$^3J(H_{6eq},H_{5eq})$=2.2 Hz, 1H; H-6eq), 3.95 (td, $^3J(H_{6ax},H_{6eq})$=$^3J(H_{5ax},H_{6ax})$=12.0 Hz, $^3J(H_{6ax},H_{5eq})$=2.0 Hz, 1H; H-6ax), 3.02 (m, 1H; H-5ax), 2.89 (dd, $^3J(H_{2ax},H_{3ax})$=10.6 Hz, $^3J(H_{3ax},H_{3eq})$=13.7 Hz, 1H; H-3ax), 2.51 (brd, $^3J(H_{3ax},H_{3eq})$=13.7 Hz, 1H; H-3eq), 2.24 ppm (dq, $^3J(H_{5ax},H_{5eq})$=13.7 Hz, $^3J(H_{5eq},H_{6ax})$=$^3J(H_{5eq},H_{6eq})$=$^4J(H_{5eq},H_{3eq})$=2.2 Hz; ^{13}C NMR (75 MHz, $CDCl_3$, 300 K): δ=142.1 (Ar-C), 128.6 (Ar-C), 128.0 (Ar-C), 125.8 (Ar-C), 80.5 (C-2), 69.9 (C-6), 33.4 (C-3), 26.4 ppm (C-5) [Lit.][6] 80.4, 69.8, 33.2, 26.3 ppm; IR (thin film): ν=2909 cm^{-1} (C=H); HRMS (EI): m/z calcd for $C_{10}H_{12}O_2S^+$: 180.0609 [M^+]; found: 180.0614.

2-Phenyl-1,4-oxathiane-*S*-oxide (rac-33a)

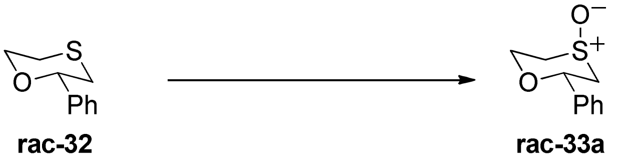

Tf_2O (196 µL, 1.16 mmol) was added to a solution of 2-phenyl-1,4-oxathiane **rac-32** (150 mg, 0.83 mmol), diphenyl sulfoxide (471 mg, 2.33 mmol) and DTBMP (513 mg, 0.25 mmol) in CH_2Cl_2 (5 mL) at −60 °C. After 90 min at that temperature the reaction was quenched by addition of sat. $NaHCO_3$ solution. The mixture was extracted into CH_2Cl_2, dried ($MgSO_4$) and concentrated to give a yellow oil. ^{1}H NMR spectroscopy of the crude product mixture showed a 88:12 ratio of

axial:equatorial sulfoxide isomers. The axial sulfoxide was separated by column chromatography (silica; gradient elution 2:1 to 1:8 (v/v) hexane-EtOAc) to afford **rac-33a** (104 mg, 64%) as a colourless oil. R_f=0.16 (hexane/EtOAc 1:4); ^{1}H NMR (500 MHz, CD_3OD, 298 K): δ=7.40-7.27 (m, 5H; Ar-H), 5.24 (dd, $^3J(H_{2ax},H_{3ax})$=11.0 Hz, $^3J(H_{2ax},H_{3eq})$=2.3 Hz, 1H; H-2ax), 4.57 (td, $^3J(H_{6ax},H_{6eq})$= $^3J(H_{6ax},H_{5ax})$=12.7 Hz, $^3J(H_{6ax},H_{5eq})$=1.7 Hz, 1H; H-6ax), 4.14-4.18 (m, 1H; H-6eq), 3.05-2.97 (m, 2H; H-3eq, H-5ax), 2.93 (dd, $^3J(H_{2ax},H_{3ax})$=11.0 Hz, $^3J(H_{3ax},H_{3eq})$=14.4 Hz, 1H; H-3ax), 2.84 ppm (m, 1H; H-5eq); ^{13}C NMR (75 MHz, CD_3OD, 300 K): δ=141.9 (Ar-C), 129.6 (Ar-C), 129.2 (Ar-C), 127.0 (Ar-C), 70.4 (C-2), 59.5 (C-6), 50.6 (C-3), 44.0 ppm (C-5) [Lit.][6] 80.4, 69.8, 33.2, 26.3 ppm; IR (thin film): ν=2920 (C-H), 1033 cm^{-1} (S=O); HRMS (ESI): *m/z* calcd for $C_{10}H_{12}O_2S+H^+$: 197.0636 [M+H^+]; found: 197.0641.

Reaction of ^{18}O-enriched diphenyl sulfoxide [^{18}O]-5 with 2-phenyl-1,4-oxathiane-*S*-oxide rac-33 in the presence of trifluoromethanesulfonic anhydride.

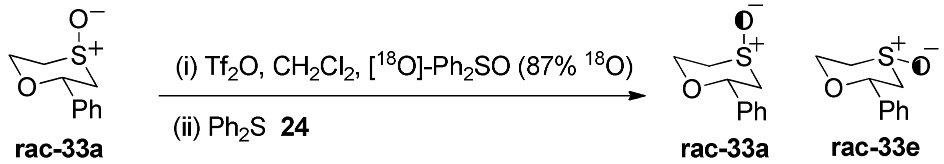

Tf_2O (36 µL, 214 µmol) was added to a solution of diphenyl sulfoxide (87% ^{18}O, 87 mg, 429 µmol) and DTBMP (94 mg, 459 µmol) in CH_2Cl_2 (1 mL) at −60 °C. After 10 min, 2-phenyl-1,4-oxathiane-*S*-oxide **rac-33a** (30 mg, 153 µmol) was added and the reaction mixture was stirred at −60 °C for 90 min. Diphenyl sulfide (36 µL, 214 µmol) was then added and the reaction mixture was warmed to RT over 10 min. The mixture was then quenched with aq. $NaHCO_3$ (1 mL), diluted with CH_2Cl_2 (5 mL), washed with aq. NaCl (2 x 5 mL), dried ($MgSO_4$) and concentrated to leave a colourless oil. ^{1}H NMR spectroscopy and ESIMS showed that the crude 2-phenyl-1,4-oxathiane-*S*-oxide **rac-3a** was an 85:15 ratio of axial:equatorial isomers that was 37% enriched in ^{18}O.

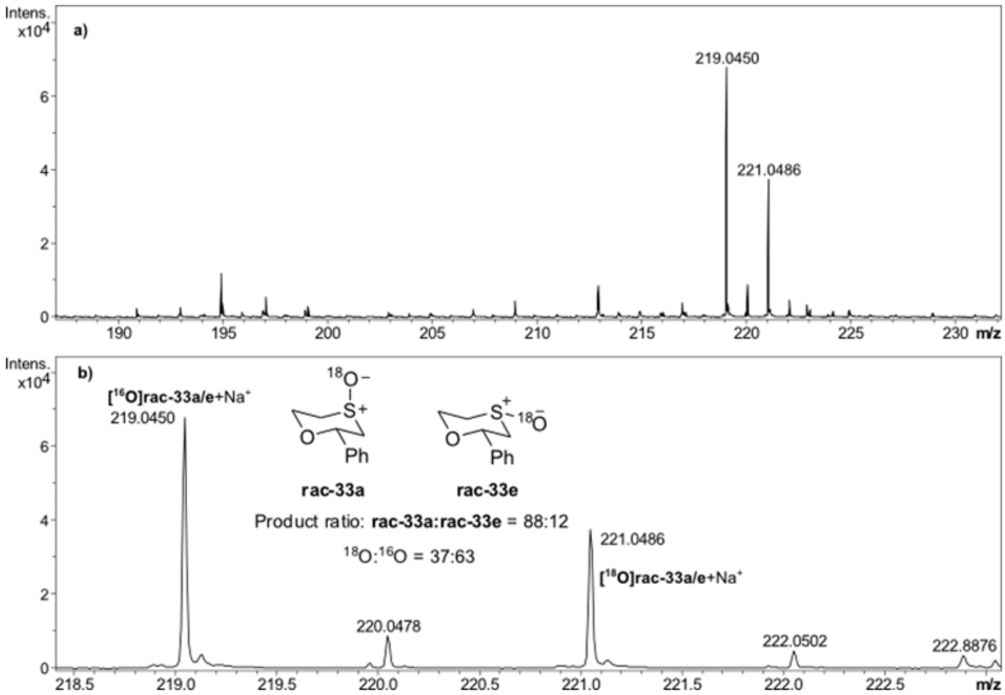

Fig. S12. a) Mass spectrum and expansion of b) **[^{18}O]-rac33a/e** (37% ^{18}O incorporation).

Density functional theory calculations

For each of the species studied, a range of conformations were generated using the Monte-Carlo conformational searching approach implemented in MacroModel from Schrodinger Inc.[7] This used the OPLS2005 forcefield.[8] In order to generate conformations, the molecules were transformed where needed such that S^+ atoms were modeled as N and bridging O atoms in S-O-S structures as CH_2. The resulting structures were subsequently transformed to the structure of interest by text editing. Conformations thus generated were subject to optimization with B3LYP/6-31G* in Gaussian09[9] and the resulting structures were all verified as minima by calculation of second derivatives.[10] The lowest energy conformations of each species were then subject to a reoptimisation with M06/6-31G*.[11] A series of single points using B3LYP/6-311+G**, M06/6-311+G** and MPWB95/AUG-CC-PVTZ were performed on the B3LYP/6-31G* geometries, also in Gaussian09.[9, 12] Finally, solvation free energies were computed using the IEFPCM model and either UAKS or Bondi radii in Gaussian03,[13] in all cases using the B3LYP/6-31+G* electronic structure method and default settings for other parameters for the dichloromethane solvent.[14] Vibrational corrections to free energies for the gas phase structures utilized the values computed for B3LYP/6-31G* at 213K.

The calculations support the hypothesis that all of the intermediates proposed in Scheme 3 are feasible structures with the exception of bis-sulfurane **10** which exhibited no barrier to dissociation in the gas phase. Monocation **6** and dication **8** displayed very similar S-O-S bond angles (116-118°) and S-O bond lengths (~1.7 Å) which place the sulfur atoms in dication **8** only 2.94 Å apart (Figure S13). Dications with the structure $[R_2S\text{-}SR_2]^{2+}$ are well documented in the literature[15] and it would appear that there may be a similar bonding interaction between the two positively charged sulfur atoms in dication **8**. In contrast, the corresponding sulfur atoms in sulfurane **7** are over 3.5 Å apart, and its S-O bond lengths are similar to those in diphenyl sulfoxide **5** (~1.5 Å) and triflyloxy sulfonium ion **6** (~1.7 Å); as such, sulfurane **7** more closely resembles an adduct in which sulfoxide **5** and monocation **6** are connected through a long S-O bond (2.36 Å). Sulfurane **9** is similar in structure to sulfurane **7** except in the lengths of the apical S-O bonds. It is possible that sulfuranes **7** and **9** are intermediates in addition-elimination process that would allow the interconversion of monocation **6** and dication **8**, or triflate exchange in **6**.[16]

Structures optimised using B3LYP/6-31G* or M06/6-31-G* in Gaussian09 were virtually identical; the only notable difference was a small adjustment of bond angles in the M06/6-31G* structure of sulfurane **7** to allow a π-π interaction between two of the phenyl groups. In contrast, single point calculations on the optimised structures employing a broad range of methods failed to give a consistent estimate of the relative energies of the various species. For example, while gas phase B3LYP/6-31G* and M06/6-31G* values were in generally good agreement (Table S1), the

other gas phase values and those with solvation corrections (Tables S2-S3) were quite divergent. Therefore, although the calculations indicate that most of the intermediates depicted in Scheme 3a are feasible structures, it was not possible to predict reliably the relative abundance of each species, nor the energy barriers separating them. Considering also the challenges of comparing the energies of species with different charges,[17] no further analysis of these values was deemed appropriate.

Comparisons of oxathiane rings bearing axial or equatorial substitutents are given in tables S4-S6. In general, ΔG° values for the oxathiane-*S*-oxides were consistent across the range of methods used (within 1 kcal mol^{-1}). However, calculations on the putative charged intermediates (dithia dications, oxodisulfonium and triflyloxysulfonium ions) were again more divergent (range greater than 2 kcal mol^{-1} in the gas phase).

Coordinates for all optimised structures are given on pages S29-S40.

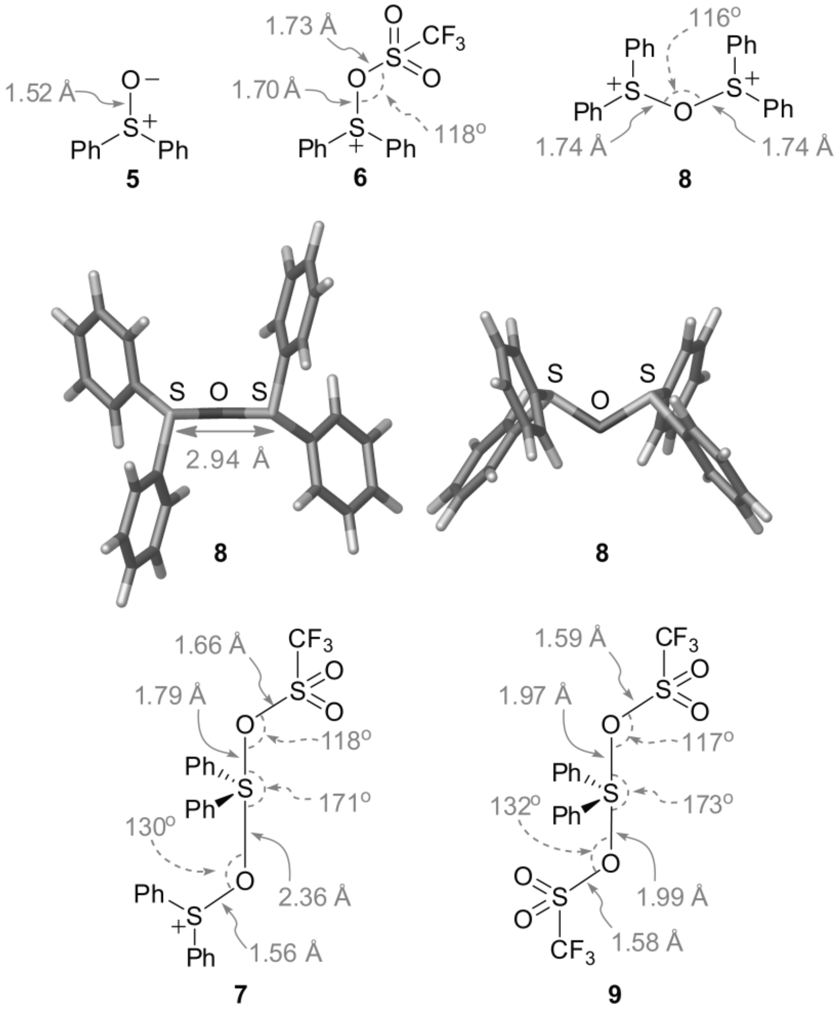

Fig. S13. Structures of putative intermediates **5-9**.

Table S1. Gas phase energy calculations for equilibria depicted in Scheme 3[a]

**Optimisation method**	**B3LYP/6-31G***				**M06/6-31G***
Single point energy calculation	B3LYP/6-31G*	B3LYP/6-311+G**	M06/6-311+G**	MPWB95/AUG-CC-PVTZ	M06/6-31G*
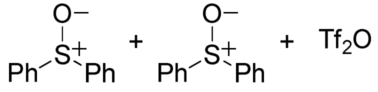 5 5	0.0	0.0	0.0	0.0	0.0
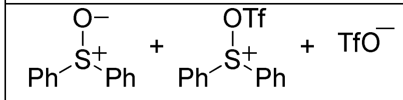 5 6	81.8	73.8	76.0	78.8	82.0
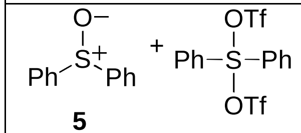 5 7	1.8	-0.5	-6.8	2.3	-0.2
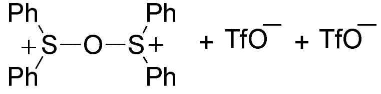 8	212.5	194.7	200.1	205.4	213.5
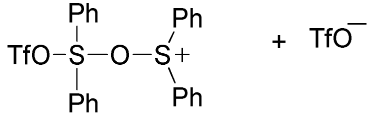 9	71.2	63.2	59.1	–	68.8

[a] free energies at 213 K in kcal mol^{-1} relative to two moles of diphenyl sulfoxide and 1 mole of triflic anhydride.

Table S2. Dichloromethane solution energy calculations for equilibria depicted in Scheme 3 (IEFPCM(UAKS)+B3LYP method) [a]

**Optimisation method**	**B3LYP/6-31G***				**M06/6-31G***
Single point energy calculation	B3LYP/6-31G*	B3LYP/6-311+G**	M06/6-311+G**	MPWB95/AUG-CC-PVTZ	M06/6-31G*
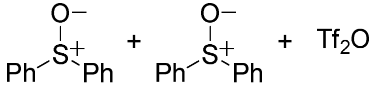 **5** **5**	0.0	0.0	0.0	0.0	0.0
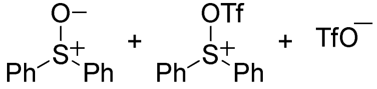 **5** **6**	9.4	1.5	3.6	6.4	8.4
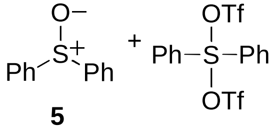 **5** **7**	3.9	1.6	-4.7	4.3	-2.9
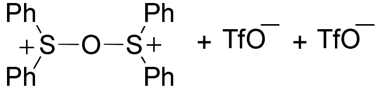 **8**	28.5	10.8	16.2	21.5	27.9
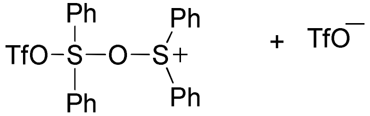 **9**	11.7	3.6	-0.4	–	3.6

[a] free energies at 213K (gas phase) with default settings for solvation free energy using UAKS radii in dichloromethane in kcal mol^{-1} relative to two moles of diphenyl sulfoxide and 1 mole of triflic anhydride.

Table S3. Dichloromethane solution energy calculations for equilibria depicted in Scheme 3 (IEFPCM(BONDI)+B3LYP method) [a]

**Optimisation method**	**B3LYP/6-31G***				**M06/6-31G***
Single point energy calculation	B3LYP/6-31G*	B3LYP/6-311+G**	M06/6-311+G**	MPWB95/AUG-CC-PVTZ	M06/6-31G*
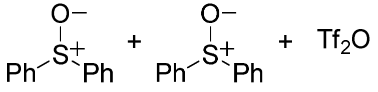 **5** **5**	0.0	0.0	0.0	0.0	0.0
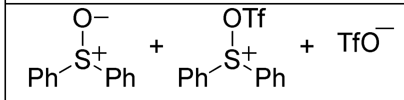 **5** **6**	1.8	-6.1	-3.9	-1.2	0.8
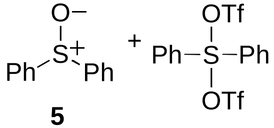 **5** **7**	7.0	4.7	-1.6	7.4	0.2
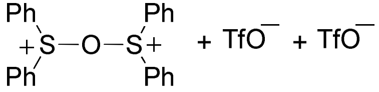 **8**	11.6	-6.1	-0.7	4.6	11.1
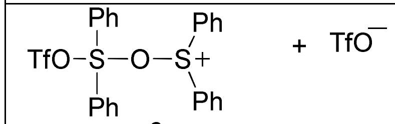 **9**	13.4	5.4	1.3	–	5.3

[a] free energies at 213K (gas phase) with default settings for solvation free energy using Bondi radii in dichloromethane in kcal mol^{-1} relative to two moles of diphenyl sulfoxide and 1 mole of triflic anhydride.

Table S4. Gas phase energy calculations for selected axial-equatorial equilibria[a]

Optimisation method	B3LYP/6-31G*				M06/6-31G*
Single point energy calculation	B3LYP/6-31G*	B3LYP/6-311+G**	M06/6-311+G**	MPWB95/AUG-CC-PVTZ	M06/6-31G*
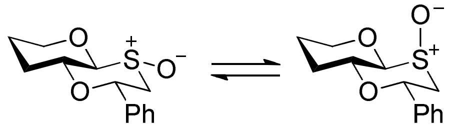 ΔG(AX – EQ) sulfoxide 1	-1.4	-0.8	-1.6	-1.4	-1.8
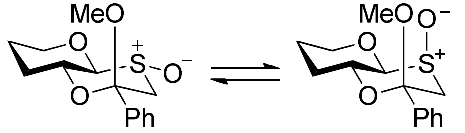 ΔG(AX – EQ) sulfoxide 2	1.9	2.9	2.4	2.1	1.9
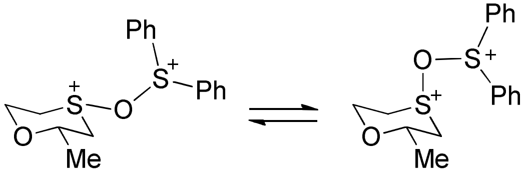 CYCLICS_OSPh2 ΔG(AX – EQ)	1.8	1.8	0.3	2.5	1.1
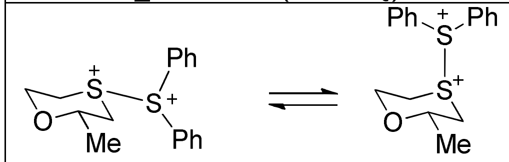 CYCLICS_SPh2 ΔG(AX – EQ)	3.4	2.9	-0.1	4.2	-0.1
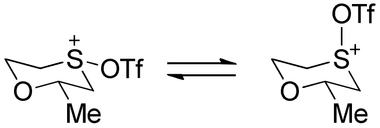 CYCLICS_Otf ΔG(AX – EQ)	0.4	0.3	-1.2	0.9	0.8
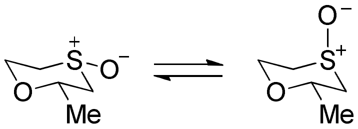 CYCLICS_O ΔG(AX – EQ)	-1.2	-0.8	-1.4	-1.1	-2.0

[a] free energies at 213 K in kcal mol^{-1}.

Table S5. Dichloromethane solution energy calculations for selected axial-equatorial equilibria (IEFPCM(UAKS)+B3LYP method) [a]

Optimisation method	B3LYP/6-31G*				M06/6-31G*
Single point energy calculation	B3LYP/6-31G*	B3LYP/6-311+G**	M06/6-311+G**	MPWB95/AUG-CC-PVTZ	M06/6-31G*
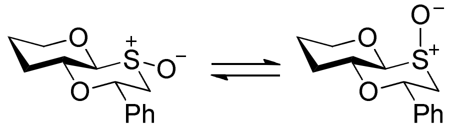<br>ΔG(AX – EQ) sulfoxide 1	-0.2	0.4	-0.4	-0.2	-0.9
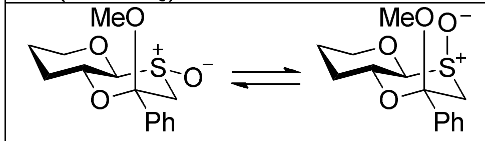<br>ΔG(AX – EQ) sulfoxide 2	2.2	3.2	2.7	2.4	1.8
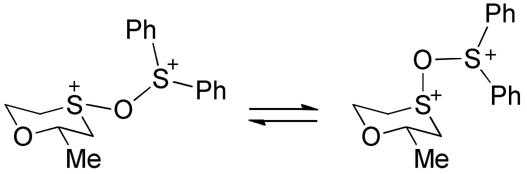<br>CYCLICS_OSPh2 ΔG(AX – EQ)	1.8	1.8	0.3	2.5	0.5
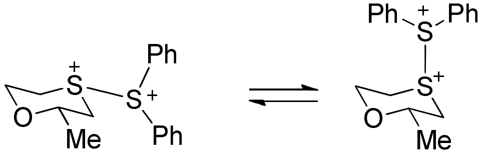<br>CYCLICS_SPh2 ΔG(AX – EQ)	2.8	2.4	-0.7	3.6	0.7
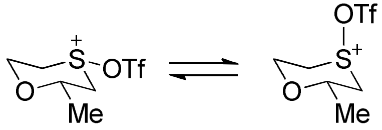<br>CYCLICS_Otf ΔG(AX – EQ)	1.1	1.0	-0.5	1.7	-0.2
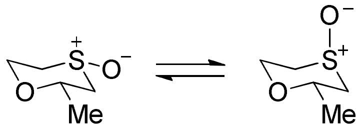<br>CYCLICS_O ΔG(AX – EQ)	-0.4	0.0	-0.6	-0.3	-0.9

[a] free energies at 213K (gas phase) with default settings for solvation free energy using UAKS radii in dichloromethane in kcal mol^{-1}.

Table S6. Dichloromethane solution energy calculations for selected axial-equatorial equilibria (IEFPCM(BONDI)+B3LYP method) [a]

Optimisation method	B3LYP/6-31G*				M06/6-31G*
Single point energy calculation	B3LYP/6-31G*	B3LYP/6-311+G**	M06/6-311+G**	MPWB95/AUG-CC-PVTZ	M06/6-31G*
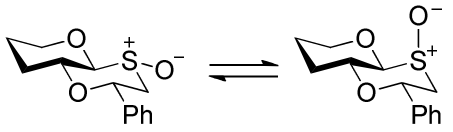 ΔG(AX – EQ) sulfoxide 1	-0.3	0.2	-0.6	-0.3	-1.1
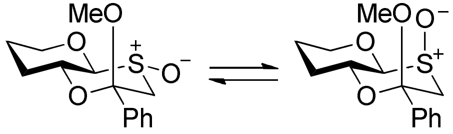 ΔG(AX – EQ) sulfoxide 2	1.6	2.6	2.1	1.7	1.2
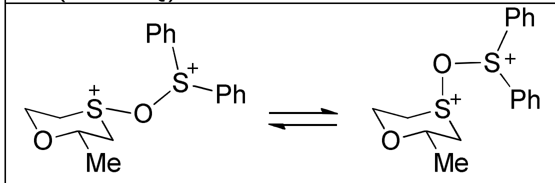 CYCLICS_OSPh2 ΔG(AX – EQ)	0.4	0.4	-1.1	1.2	-0.8
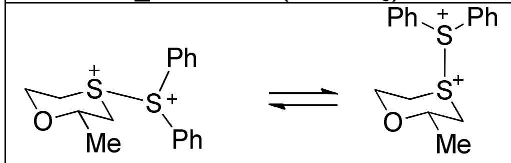 CYCLICS_SPh2 ΔG(AX – EQ)	1.1	0.6	-2.5	1.9	-1.1
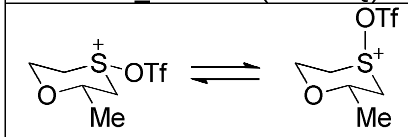 CYCLICS_Otf ΔG(AX – EQ)	-1.1	-1.2	-2.7	-0.5	-2.4
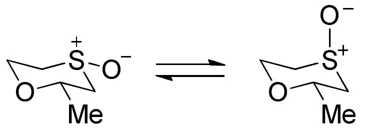 CYCLICS_O ΔG(AX – EQ)	-0.3	0.1	-0.5	-0.2	-0.8

[a] free energies at 213K (gas phase) with default settings for solvation free energy using Bondi radii in dichloromethane in kcal mol^{-1}.

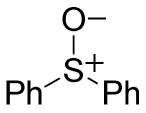

5

Ph2SO

B3LYP geometry

C	-1.986586	0.725761	0.843538
C	-3.070526	0.033143	1.384604
C	-3.543441	-1.125879	0.764180
C	-2.939768	-1.591857	-0.406366
C	-1.857446	-0.903311	-0.957081
C	-1.382127	0.239285	-0.313117
S	0.002158	1.199037	-1.029326
O	0.018442	2.532781	-0.310138
C	1.378593	0.218923	-0.321705
C	2.067878	0.757645	0.761546
C	3.152021	0.056252	1.292377
C	3.540220	-1.163456	0.733827
C	2.849647	-1.683880	-0.363939
C	1.766702	-0.988376	-0.903359
H	-1.607536	1.640667	1.289670
H	-3.549430	0.400234	2.288447
H	-4.390177	-1.660575	1.185837
H	-3.317416	-2.484793	-0.897099
H	-1.398207	-1.251839	-1.879054
H	1.750418	1.716394	1.161141
H	3.696191	0.464414	2.139937
H	4.387005	-1.705013	1.146528
H	3.159095	-2.627089	-0.805971
H	1.236993	-1.384744	-1.766155

M06 Geometry

C	-1.923007	0.656522	0.876668
C	-2.953688	-0.120494	1.394287
C	-3.391735	-1.248186	0.705477
C	-2.808383	-1.601499	-0.508537
C	-1.779738	-0.828534	-1.036169
C	-1.343015	0.283273	-0.326759
S	-0.000025	1.321264	-0.977407
O	0.000103	2.573194	-0.148406
C	1.342990	0.283160	-0.326835
C	1.923168	0.656592	0.876448
C	2.953874	-0.120382	1.394073
C	3.391751	-1.248237	0.705411
C	2.808200	-1.601728	-0.508450
C	1.779514	-0.828800	-1.036091
H	-1.560844	1.554748	1.376246
H	-3.420240	0.155798	2.338366
H	-4.200453	-1.852323	1.112787
H	-3.161017	-2.477600	-1.049870
H	-1.324832	-1.088374	-1.993068
H	1.561195	1.554948	1.375935
H	3.420535	0.156067	2.338053
H	4.200491	-1.852357	1.112700
H	3.160705	-2.477945	-1.049677
H	1.324459	-1.088857	-1.992859

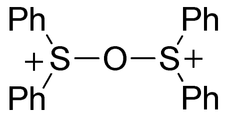

8

Ph2SOSPh2_CONF_7

B3LYP/6-31G*Geometry

C	-3.007121	1.192914	-2.032208
C	-3.819542	2.319167	-2.162777
C	-3.937311	3.223212	-1.104766
C	-3.243267	3.016000	0.095386
C	-2.440114	1.891181	0.258576
C	-2.344141	0.986892	-0.811779
S	-1.385255	-0.501491	-0.739558
C	-2.112347	-1.635602	0.394042
C	-2.971595	-1.252462	1.438847
C	-3.510925	-2.246850	2.246553
C	-3.203586	-3.593870	2.011904
C	-2.355581	-3.963392	0.962063
C	-1.803920	-2.987343	0.139261
O	0.000066	-0.003818	0.187054
S	1.384300	0.499037	-0.736714
C	2.347357	-0.986989	-0.810045
C	2.442078	-1.894231	0.257832
C	3.249482	-3.015978	0.094253
C	3.948772	-3.217287	-1.103827
C	3.832150	-2.310329	-2.159450
C	3.015681	-1.187031	-2.028481
C	2.108089	1.634027	0.398404
C	1.807887	2.986130	0.136281
C	2.357157	3.962621	0.960290
C	3.193947	3.592915	2.018945
C	3.492673	2.245263	2.261294
C	2.956028	1.250550	1.452254
H	-2.901295	0.493027	-2.856223
H	-4.349453	2.491795	-3.093889
H	-4.565597	4.101589	-1.215770
H	-3.331236	3.733429	0.905229
H	-1.885084	1.734121	1.177560
H	-3.232509	-0.213888	1.602187
H	-4.182182	-1.974621	3.054618
H	-3.640612	-4.360771	2.644182
H	-2.138135	-5.010217	0.776625
H	-1.160413	-3.266252	-0.690119
H	1.882883	-1.742014	1.175098
H	3.336612	-3.735634	0.902212
H	4.580261	-4.093321	-1.215137
H	4.366087	-2.478398	-3.089094
H	2.910734	-0.484991	-2.850777
H	1.172771	3.264960	-0.699556
H	2.146329	5.009775	0.769148
H	3.628926	4.360021	2.652385
H	4.155147	1.972823	3.076508
H	3.210423	0.211322	1.621635

M06/6-31G*Geometry

C	2.963446	-0.980504	-2.131827
C	3.785225	-2.084540	-2.325086
C	3.906372	-3.044185	-1.324287
C	3.209874	-2.918194	-0.119623
C	2.396383	-1.818341	0.105878
C	2.295750	-0.859610	-0.909610
S	1.311426	0.590559	-0.737779

C	2.026949	1.639755	0.469243
C	2.966392	1.201631	1.409556
C	3.469248	2.128358	2.308417
C	3.043970	3.457216	2.261003
C	2.115914	3.881920	1.310304
C	1.602256	2.975523	0.397170
O	0.000094	0.000074	0.184596
S	-1.311069	-0.590668	-0.737718
C	-2.295534	0.859395	-0.909828
C	-2.396385	1.818202	0.105558
C	-3.210104	2.917861	-0.120070
C	-3.906610	3.043586	-1.324752
C	-3.785242	2.083861	-2.325449
C	-2.963247	0.980009	-2.132059
C	-2.026796	-1.639599	0.469420
C	-1.602629	-2.975535	0.397398
C	-2.116591	-3.881658	1.310642
C	-3.044385	-3.456513	2.261396
C	-3.469107	-2.127475	2.308774
C	-2.965953	-1.201020	1.409800
H	2.849133	-0.229676	-2.912724
H	4.321382	-2.198591	-3.263753
H	4.545860	-3.908960	-1.485349
H	3.309301	-3.681983	0.648001
H	1.839228	-1.712600	1.035828
H	3.315883	0.172112	1.423255
H	4.206117	1.817538	3.044634
H	3.454592	4.175563	2.967210
H	1.808826	4.923864	1.272176
H	0.898045	3.297458	-0.369436
H	-1.839221	1.712682	1.035526
H	-3.309705	3.681700	0.647482
H	-4.546294	3.908199	-1.485904
H	-4.321416	2.197693	-3.264132
H	-2.848768	0.229129	-2.912882
H	-0.898641	-3.297823	-0.369265
H	-1.809949	-4.923735	1.272544
H	-3.455253	-4.174646	2.967677
H	-4.205768	-1.816302	3.045049
H	-3.314994	-0.171341	1.423455

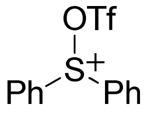

6

Ph2SOTf_CONF_20
B3LYP/6-31G*Geometry

C	0.188001	2.743333	-0.848002
C	0.465559	4.075712	-0.555934
C	1.469623	4.393886	0.362891
C	2.205088	3.385640	0.996625
C	1.942164	2.046752	0.723865
C	0.927580	1.742369	-0.194889
S	0.482255	0.088888	-0.649107
C	1.846568	-0.989292	-0.284353
C	2.184418	-1.352719	1.026607
C	3.261080	-2.216868	1.209331
C	3.978856	-2.694855	0.106760
C	3.617737	-2.330042	-1.191924
C	2.532595	-1.479879	-1.402822
O	-0.594230	-0.268916	0.614499
S	-1.877715	-1.377460	0.285530
O	-1.556839	-2.062874	-0.952204
O	-2.126293	-2.014131	1.556222
C	-3.221104	-0.092429	-0.059932
F	-3.456807	0.593302	1.041539
F	-2.768751	0.714193	-1.023637
F	-4.297336	-0.737389	-0.464962
H	-0.587231	2.485939	-1.563591
H	-0.094877	4.862523	-1.050219
H	1.687748	5.434764	0.581462
H	2.988712	3.643853	1.701623
H	2.522545	1.264843	1.199524
H	1.603143	-1.001069	1.872386
H	3.535190	-2.523617	2.213713
H	4.815338	-3.369027	0.262759
H	4.165594	-2.719815	-2.043632
H	2.227136	-1.214504	-2.410720

M06/6-31G*Geometry

C	0.131554	2.676496	-0.040936
C	0.592890	3.961953	0.199569
C	1.961461	4.225739	0.211826
C	2.880850	3.206679	-0.012962
C	2.444918	1.906842	-0.242536
C	1.072208	1.661515	-0.234349
S	0.450103	0.057514	-0.685101
C	1.700427	-1.095381	-0.266889
C	2.326681	-1.080078	0.986296
C	3.249537	-2.074032	1.262872
C	3.533303	-3.053295	0.307191
C	2.890851	-3.062059	-0.927708
C	1.949788	-2.084624	-1.222929
O	-0.637331	-0.087979	0.597088
S	-1.925748	-1.157061	0.389389
O	-1.603691	-2.004863	-0.730993
O	-2.250217	-1.601377	1.710871
C	-3.173810	0.107288	-0.163163
F	-3.371409	0.958990	0.812835
F	-2.667151	0.732836	-1.214935
F	-4.272778	-0.521077	-0.483225
H	-0.936068	2.466676	-0.051200
H	-0.121959	4.762107	0.374104
H	2.313151	5.238525	0.393742
H	3.946961	3.419264	-0.009446
H	3.163716	1.111701	-0.425194
H	2.081078	-0.318453	1.724480
H	3.747488	-2.094611	2.229044
H	4.260753	-3.828634	0.537055
H	3.109642	-3.838447	-1.656013
H	1.413572	-2.090625	-2.170670

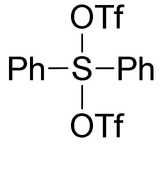

7

Ph2SOTf2_CONF15
B3LYP/6-31G*Geometry

F	-4.818562	0.831425	0.628637
C	-4.478531	-0.015131	-0.342120
F	-4.192170	-1.206957	0.189554

F	-5.493563	-0.146531	-1.195813
S	-2.997514	0.635955	-1.279844
O	-2.600259	-0.410907	-2.224637
O	-3.350101	1.966579	-1.753940
O	-1.985622	0.702928	-0.058473
S	-0.117295	0.163168	-0.400209
O	1.727773	-0.321699	-0.949801
S	2.982028	-0.829395	-0.129706
O	3.096664	-0.151654	1.163445
O	3.130865	-2.277238	-0.211978
C	4.304524	-0.094658	-1.231150
F	4.141778	1.232009	-1.328533
F	5.495538	-0.345493	-0.685983
F	4.249249	-0.632780	-2.446725
C	0.382326	1.536576	0.666493
C	1.347475	2.407530	0.157802
C	1.690387	3.534295	0.903668
C	1.076131	3.780074	2.131832
C	0.105065	2.902934	2.619475
C	-0.257314	1.774933	1.886041
C	-0.343883	-1.296325	0.635397
C	-1.117410	-2.312504	0.063555
C	-1.297661	-3.493581	0.779864
C	-0.709943	-3.651980	2.036747
C	0.067645	-2.630238	2.583289
C	0.263371	-1.438467	1.884992
H	1.840722	2.199978	-0.783356
H	2.444402	4.215145	0.521181
H	1.350448	4.658411	2.708748
H	-0.381261	3.097578	3.570570
H	-1.034464	1.111955	2.244941
H	-1.576426	-2.177582	-0.910210
H	-1.896931	-4.290185	0.349857
H	-0.850907	-4.577517	2.587314
H	0.539005	-2.758877	3.552785
H	0.901824	-0.664421	2.291842

M06/6-31G*Geometry

F	-4.690337	0.661991	0.686270
C	-4.273960	-0.290598	-0.127155
F	-3.724331	-1.264461	0.579414
F	-5.294089	-0.767262	-0.816572
S	-3.046295	0.400085	-1.314176
O	-2.535300	-0.715557	-2.091808
O	-3.691905	1.523608	-1.950932
O	-1.972187	0.882707	-0.274251
S	-0.152967	0.310154	-0.547928
O	1.687467	-0.229196	-0.955065
S	2.775208	-0.789735	0.019910
O	2.945427	0.061860	1.185352
O	2.675994	-2.226272	0.175493
C	4.230417	-0.468284	-1.067538
F	4.298282	0.824305	-1.355101
F	5.321476	-0.828032	-0.414992
F	4.132870	-1.162287	-2.184743
C	0.351736	1.791194	0.351924
C	1.416868	2.513668	-0.177450
C	1.782191	3.706376	0.433247
C	1.093211	4.162477	1.550926
C	0.023158	3.433396	2.059877
C	-0.364971	2.243680	1.458059
C	-0.330980	-1.027188	0.631638
C	-0.768770	-2.225380	0.069719
C	-0.842073	-3.346754	0.882856
C	-0.470249	-3.265079	2.221067
C	-0.022315	-2.062540	2.757204
C	0.059398	-0.925960	1.962633
H	1.967600	2.141693	-1.036457
H	2.615862	4.276685	0.030615
H	1.387858	5.095632	2.026511
H	-0.524491	3.794005	2.927669
H	-1.226338	1.695480	1.827845
H	-1.057754	-2.270992	-0.978745
H	-1.182964	-4.290675	0.464187
H	-0.517392	-4.151478	2.850227
H	0.286452	-2.007556	3.798445
H	0.463297	-0.000401	2.363969

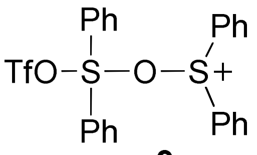

9

Ph2SOTfOSPh2_CONF9
B3LYP/6-31G*Geometry

F	5.109153	0.865630	-1.418700
C	4.285895	-0.068910	-1.866445
F	3.177188	0.496733	-2.355094
F	4.871457	-0.802613	-2.798960
S	3.791410	-1.200749	-0.450240
O	2.851316	-2.180566	-0.978263
O	4.973430	-1.544940	0.308405
O	2.961177	-0.109565	0.483528
S	1.204687	0.060712	0.198263
C	0.691426	-0.752667	1.713422
C	0.197165	-0.053826	2.818556
C	-0.193528	-0.778624	3.945123
C	-0.096286	-2.171262	3.957360
C	0.384155	-2.854931	2.836970
C	0.775833	-2.151832	1.699893
C	1.226469	1.829832	0.516559
C	2.020662	2.358217	1.543960
C	2.089613	3.742414	1.684941
C	1.387762	4.576645	0.809832
C	0.613401	4.031857	-0.216078
C	0.527538	2.649008	-0.376731
O	-1.038805	0.152518	-0.521276
S	-2.354828	-0.097015	0.278055
C	-3.527445	1.140618	-0.305917
C	-4.215061	1.876777	0.661039
C	-5.124120	2.853630	0.247205
C	-5.321219	3.089622	-1.113644
C	-4.614448	2.353007	-2.071619
C	-3.712100	1.370101	-1.673316
C	-3.044008	-1.612785	-0.438998
C	-2.415927	-2.203575	-1.533712
C	-2.948238	-3.386744	-2.051489
C	-4.082823	-3.959462	-1.473972
C	-4.692888	-3.357066	-0.369842
C	-4.171812	-2.177765	0.161171
H	0.115420	1.026475	2.807830
H	-0.572156	-0.249109	4.813932
H	-0.397735	-2.726078	4.840629
H	0.456875	-3.937839	2.845333
H	1.155571	-2.678556	0.829799

H	2.589354	1.710129	2.200551
H	2.700822	4.167451	2.474948
H	1.452230	5.654457	0.924621
H	0.075635	4.681442	-0.899510
H	-0.082780	2.213605	-1.158021
H	-4.047174	1.691819	1.718513
H	-5.671519	3.429282	0.987339
H	-6.027705	3.850140	-1.432699
H	-4.773461	2.541919	-3.129095
H	-3.162737	0.788220	-2.406804
H	-1.529605	-1.746570	-1.960831
H	-2.473124	-3.859720	-2.905850
H	-4.490855	-4.880067	-1.880303
H	-5.571903	-3.807145	0.081532
H	-4.642691	-1.708039	1.020849

M06/6-31G*Geometry

F	5.284758	1.080438	-0.513667
C	4.522295	0.526743	-1.425721
F	3.524908	1.341195	-1.737386
F	5.214947	0.223733	-2.496623
S	3.793471	-1.033024	-0.741532
O	2.921813	-1.592245	-1.749985
O	4.823305	-1.766642	-0.063282
O	2.871515	-0.353417	0.431248
S	1.214960	0.033066	0.018527
C	0.478840	-1.336753	0.894678
C	0.032844	-1.259221	2.210918
C	-0.563298	-2.384121	2.771189
C	-0.723892	-3.542981	2.017226
C	-0.285289	-3.594842	0.696266
C	0.321207	-2.487687	0.120432
C	1.186599	1.462540	1.096196
C	1.884578	1.453154	2.306203
C	1.911911	2.617393	3.062044
C	1.267615	3.765759	2.608100
C	0.593058	3.762014	1.391521
C	0.548315	2.607776	0.619080
O	-0.932640	0.454411	-0.694523
S	-2.168142	0.375214	0.231527
C	-3.324335	1.551411	-0.482387
C	-4.434387	1.905990	0.277793
C	-5.346849	2.807051	-0.257067
C	-5.133874	3.343608	-1.524390
C	-4.011585	2.983060	-2.264937
C	-3.092397	2.076512	-1.747445
C	-2.957608	-1.181320	-0.185827
C	-2.811629	-1.706956	-1.465285
C	-3.401552	-2.931826	-1.748789
C	-4.122485	-3.606512	-0.764030
C	-4.257075	-3.066103	0.510646
C	-3.664002	-1.842992	0.811414
H	0.130887	-0.342928	2.788227
H	-0.908867	-2.348716	3.801830
H	-1.199136	-4.414401	2.462587
H	-0.425036	-4.497675	0.106385
H	0.668368	-2.512246	-0.912315
H	2.417673	0.565970	2.639699
H	2.450391	2.628020	4.006696
H	1.299689	4.673991	3.205809
H	0.102340	4.663943	1.033769
H	0.015257	2.587714	-0.328492
H	-4.585615	1.485537	1.272640
H	-6.222429	3.096149	0.319838
H	-5.848125	4.053241	-1.936302
H	-3.851317	3.408746	-3.253207
H	-2.205838	1.783669	-2.306495
H	-2.229031	-1.169023	-2.211928
H	-3.302117	-3.363281	-2.742628
H	-4.583501	-4.564698	-0.994557
H	-4.818029	-3.598789	1.275447
H	-3.745650	-1.414652	1.811109

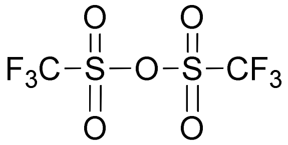

Tf2O_CONF_8
B3LYP/6-31G*Geometry

F	3.572202	-0.074402	-0.690226
C	2.542949	0.413899	-0.007663
F	2.903528	0.689385	1.236703
F	2.069290	1.501175	-0.602555
S	1.208532	-0.915011	0.014838
O	0.839295	-1.228047	-1.349549
O	1.567723	-1.910127	1.001873
O	0.000004	0.000015	0.773661
S	-1.208545	0.915024	0.014840
O	-1.567757	1.910125	1.001881
O	-0.839319	1.228072	-1.349546
C	-2.542936	-0.413912	-0.007664
F	-3.572188	0.074368	-0.690246
F	-2.069249	-1.501184	-0.602540
F	-2.903521	-0.689388	1.236702

M06/6-31G*Geometry

F	3.524466	-0.097496	-0.660324
C	2.494068	0.412209	-0.020104
F	2.826511	0.733786	1.208150
F	2.028542	1.461690	-0.660227
S	1.184261	-0.904609	0.028806
O	0.797281	-1.218170	-1.318795
O	1.562840	-1.881013	1.011180
O	0.000000	0.000002	0.783085
S	-1.184262	0.904610	0.028804
O	-1.562844	1.881014	1.011178
O	-0.797280	1.218171	-1.318796
C	-2.494067	-0.412210	-0.020107
F	-3.524465	0.097494	-0.660329
F	-2.028540	-1.461690	-0.660229
F	-2.826512	-0.733787	1.208147

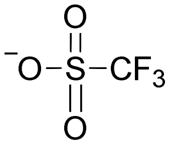

TRIFLATE_1
B3LYP/6-31G*Geometry

O	-1.244086	-0.505285	1.354999
S	-0.926202	0.000005	0.000077
O	-1.244628	-0.920708	-1.114927
O	-1.244065	1.426087	-0.239770
C	0.941476	-0.000063	-0.000046
F	1.445825	-1.237167	0.207074

F	1.445358	0.439092	-1.175143
F	1.445774	0.798024	0.967694

M06/6-31G*Geometry

O	1.223084	1.323287	-0.558612
S	0.912641	0.000310	-0.000088
O	1.224401	-1.144754	-0.866283
O	1.224187	-0.177522	1.424692
C	-0.929135	-0.000112	0.000117
F	-1.422614	0.150523	-1.234524
F	-1.421124	-1.145299	0.486474
F	-1.423020	0.993402	0.748308

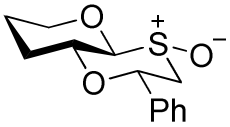

EQ_SULFOXIDE_1_c1
B3LYP/6-31G*Geometry

O	-0.064473	1.402136	-0.015531
C	1.251791	1.951270	0.099027
C	2.096623	1.606116	-1.127990
C	2.112220	0.086475	-1.357893
C	0.687465	-0.472589	-1.363745
H	0.141372	-0.074491	-2.236365
C	-0.043934	-0.003855	-0.092778
H	0.409480	-0.454146	0.806036
S	-1.827036	-0.525594	-0.116169
O	-2.389161	-0.437269	1.282118
C	-1.400922	-2.306834	-0.413574
C	-0.494239	-2.525468	-1.636533
H	-0.986306	-2.096937	-2.525588
C	-0.258869	-4.004984	-1.870710
C	0.793818	-4.673967	-1.234963
C	0.971928	-6.044563	-1.423705
C	0.098337	-6.762556	-2.242937
C	-0.952748	-6.100683	-2.879526
C	-1.126922	-4.728453	-2.696041
O	0.766683	-1.886966	-1.449106
H	1.110843	3.029981	0.205869
H	1.729239	1.572141	1.017422
H	1.671857	2.113666	-2.003324
H	3.117966	1.984147	-0.996914
H	2.607548	-0.171402	-2.300424
H	2.668547	-0.413215	-0.553388
H	-0.923448	-2.678962	0.499396
H	-2.360322	-2.819008	-0.534683
H	1.479042	-4.112270	-0.608643
H	1.796374	-6.552579	-0.930294
H	0.238931	-7.830213	-2.388344
H	-1.632747	-6.649381	-3.525624
H	-1.941832	-4.215547	-3.203073

M06/6-31G*Geometry

O	3.356514	0.280037	-0.068856
C	4.029072	-0.900822	0.336685
C	3.452058	-2.116473	-0.365992
C	1.950786	-2.198188	-0.114489
C	1.282979	-0.881389	-0.465395
H	1.383431	-0.692817	-1.554996
C	2.002301	0.249023	0.268107
H	1.850870	0.158279	1.363624
S	1.340954	1.898203	-0.218339
O	1.765686	2.894843	0.813315
C	-0.382594	1.409786	0.197400
C	-0.838779	0.146724	-0.524929
H	-0.709150	0.292921	-1.616864
C	-2.290421	-0.121107	-0.234962
C	-2.668500	-0.845536	0.894445
C	-4.014934	-1.043890	1.177032
C	-4.992841	-0.513979	0.340784
C	-4.619507	0.215415	-0.783197
C	-3.272797	0.408535	-1.069028
O	-0.075555	-0.968142	-0.112680
H	5.085088	-0.744258	0.091935
H	3.943935	-1.013373	1.434019
H	3.647524	-2.025429	-1.444961
H	3.955554	-3.028906	-0.019800
H	1.480974	-3.009551	-0.685155
H	1.752837	-2.397945	0.951355
H	-0.424899	1.272664	1.288286
H	-1.012039	2.269552	-0.064924
H	-1.898337	-1.265319	1.538882
H	-4.302532	-1.618086	2.056266
H	-6.046508	-0.673025	0.563402
H	-5.378412	0.628143	-1.445579
H	-2.977694	0.973394	-1.955056

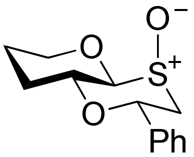

AX_SULFOXIDE_1_c1
B3LYP/6-31G*Geometry

O	-0.064177	1.389467	0.051837
C	1.259164	1.926730	0.155502
C	2.089130	1.609154	-1.088247
C	2.114619	0.093593	-1.341494
C	0.689914	-0.457951	-1.361263
H	0.141838	-0.063954	-2.230093
C	-0.060116	-0.006616	-0.103099
H	0.346332	-0.517710	0.787122
S	-1.857351	-0.502667	-0.225713
O	-2.365846	-0.048045	-1.578378
C	-1.426147	-2.300517	-0.419116
C	-0.500402	-2.522720	-1.627535
H	-0.997504	-2.087910	-2.506726
C	-0.260610	-4.001626	-1.856369
C	0.847224	-4.650808	-1.299164
C	1.027676	-6.021802	-1.486770
C	0.101957	-6.760090	-2.226230
C	-1.004864	-6.117848	-2.784672
C	-1.182045	-4.746153	-2.602873
O	0.758099	-1.879555	-1.424285
H	1.125088	3.003086	0.291214
H	1.749042	1.522819	1.057664
H	1.648133	2.127050	-1.949023
H	3.107716	1.996054	-0.960966
H	2.615097	-0.147536	-2.285886
H	2.670841	-0.414957	-0.541907
H	-0.963511	-2.656208	0.508019
H	-2.382310	-2.813411	-0.559059
H	1.572657	-4.072692	-0.736723
H	1.895851	-6.513701	-1.055623
H	0.244816	-7.827522	-2.371669

H	-1.725977	-6.682045	-3.370151
H	-2.039734	-4.247908	-3.050325

M06/6-31G*Geometry

O	-3.365913	0.359154	-0.372147
C	-4.055488	-0.880267	-0.420094
C	-3.551139	-1.830504	0.649748
C	-2.047339	-2.028542	0.501154
C	-1.353026	-0.681189	0.484871
H	-1.481772	-0.172056	1.458766
C	-1.996134	0.204658	-0.572764
H	-1.762271	-0.188101	-1.584353
S	-1.299726	1.918475	-0.478835
O	-1.361572	2.332627	0.963198
C	0.418661	1.305156	-0.744113
C	0.804806	0.283914	0.321920
H	0.633592	0.753704	1.308042
C	2.256261	-0.082094	0.177305
C	2.650797	-1.196182	-0.560908
C	4.002307	-1.478621	-0.729761
C	4.968604	-0.648444	-0.171425
C	4.578499	0.466435	0.564342
C	3.228475	0.745228	0.738699
O	0.015325	-0.883939	0.206609
H	-5.115439	-0.637455	-0.289122
H	-3.927567	-1.332510	-1.422746
H	-3.777505	-1.401864	1.637272
H	-4.082076	-2.789332	0.579410
H	-1.631718	-2.648402	1.306014
H	-1.821855	-2.540152	-0.449429
H	0.500532	0.871364	-1.751025
H	1.061147	2.192769	-0.686894
H	1.890392	-1.847912	-0.986012
H	4.301956	-2.355346	-1.301975
H	6.025608	-0.871878	-0.305821
H	5.328081	1.117686	1.010730
H	2.920086	1.613861	1.323372

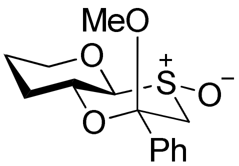

EQ_SULFOXIDE_2_c1

B3LYP/6-31G*Geometry

O	-0.023554	1.383836	0.065420
C	1.306432	1.881550	0.233524
C	2.161508	1.581525	-0.998424
C	2.130375	0.078278	-1.317396
C	0.686237	-0.424922	-1.381177
H	0.168646	0.042311	-2.229436
C	-0.049789	-0.015460	-0.095980
H	0.370045	-0.532489	0.783382
S	-1.847545	-0.476168	-0.179373
O	-2.415050	-0.466972	1.220223
C	-1.447872	-2.254416	-0.542728
C	-0.519042	-2.486250	-1.745993
O	-1.216470	-1.956265	-2.860572
C	-0.547910	-2.054447	-4.118733
C	-0.237035	-3.982887	-1.890838
C	0.973897	-4.549688	-1.480164
C	1.175764	-5.927412	-1.583593
C	0.172478	-6.750777	-2.095562
C	-1.038739	-6.189612	-2.506210
C	-1.242694	-4.814429	-2.402174
O	0.732866	-1.841312	-1.558757
H	1.198412	2.956327	0.401357
H	1.755879	1.434430	1.135477
H	1.768448	2.153192	-1.848647
H	3.192369	1.916252	-0.829436
H	2.636050	-0.138957	-2.265179
H	2.655446	-0.485867	-0.534705
H	-0.999484	-2.657002	0.370291
H	-2.411563	-2.743046	-0.703340
H	-1.181327	-1.525011	-4.833577
H	0.441137	-1.584320	-4.085660
H	-0.434311	-3.097950	-4.433460
H	1.754715	-3.909358	-1.086625
H	2.121585	-6.356516	-1.263351
H	0.332176	-7.822743	-2.174594
H	-1.825428	-6.822205	-2.908563
H	-2.181554	-4.376215	-2.729425

M06/6-31G*Geometry

O	-3.460487	0.298416	-0.165963
C	-4.105199	-0.874788	-0.632312
C	-3.541278	-2.107417	0.051876
C	-2.031748	-2.166594	-0.151052
C	-1.394311	-0.853184	0.262606
H	-1.531272	-0.700123	1.348500
C	-2.094391	0.293998	-0.455787
H	-1.905893	0.248714	-1.548519
S	-1.464262	1.928297	0.116272
O	-1.844531	2.947642	-0.912052
C	0.273035	1.462012	-0.285929
C	0.761785	0.168686	0.355768
O	0.663607	0.373558	1.743236
C	1.051705	-0.729866	2.537689
C	2.189406	-0.099283	-0.085984
C	2.489105	-1.019843	-1.086801
C	3.805849	-1.196742	-1.500716
C	4.829349	-0.456648	-0.919835
C	4.533031	0.466676	0.078817
C	3.219182	0.647004	0.490438
O	-0.018147	-0.929276	-0.057174
H	-5.171029	-0.736774	-0.421079
H	-3.979862	-0.952337	-1.728910
H	-3.774835	-2.052510	1.125655
H	-4.022984	-3.012883	-0.340696
H	-1.572686	-2.989660	0.413583
H	-1.797145	-2.335520	-1.214809
H	0.319874	1.388291	-1.381074
H	0.892612	2.308872	0.030845
H	0.866814	-0.442242	3.576198
H	0.463470	-1.626992	2.297034
H	2.117691	-0.966557	2.409370
H	1.687029	-1.600238	-1.535457
H	4.031356	-1.920869	-2.281843
H	5.858615	-0.598104	-1.244970
H	5.329512	1.047574	0.540642
H	2.980252	1.361811	1.278138

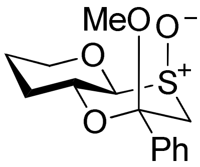

AX_SULFOXIDE_2_c1

B3LYP/6-31G*Geometry

O	0.075326	1.256610	0.321911
C	1.418755	1.747146	0.358217
C	2.098253	1.603107	-1.002601
C	2.057534	0.137275	-1.458975
C	0.624994	-0.391701	-1.402931
H	0.003362	0.092125	-2.166773
C	-0.000386	-0.098980	-0.038810
H	0.440817	-0.756469	0.730734
S	-1.828921	-0.472981	-0.095990
O	-2.422523	0.324744	-1.228339
C	-1.526491	-2.234591	-0.627712
C	-0.560355	-2.472365	-1.810017
O	-1.244321	-2.008199	-2.952878
C	-0.521595	-2.072692	-4.180548
C	-0.247458	-3.971322	-1.893221
C	0.940559	-4.508000	-1.385174
C	1.166433	-5.885078	-1.429510
C	0.209058	-6.739105	-1.978540
C	-0.978928	-6.208620	-2.485902
C	-1.206456	-4.833544	-2.441544
O	0.681244	-1.806787	-1.625417
H	1.336252	2.793532	0.663605
H	1.989231	1.205171	1.131625
H	1.576081	2.239815	-1.727415
H	3.134166	1.958462	-0.939613
H	2.455920	0.020669	-2.473340
H	2.679356	-0.479849	-0.795762
H	-1.154883	-2.748047	0.265329
H	-2.512216	-2.635343	-0.875975
H	-1.200072	-1.683757	-4.942402
H	0.382843	-1.455063	-4.148401
H	-0.238770	-3.102037	-4.431977
H	1.687291	-3.843303	-0.966269
H	2.095393	-6.289759	-1.036086
H	0.387354	-7.810585	-2.012596
H	-1.728916	-6.865144	-2.919099
H	-2.125334	-4.418334	-2.845606

M06/6-31G*Geometry

O	-3.406975	0.104712	-0.771072
C	-4.014808	-1.168855	-0.633161
C	-3.581807	-1.844223	0.654125
C	-2.064293	-1.971817	0.676148
C	-1.429005	-0.617035	0.437243
H	-1.618293	0.057553	1.290383
C	-2.016264	0.031431	-0.803487
H	-1.642380	-0.490625	-1.709370
S	-1.464686	1.798245	-0.912951
O	-1.878783	2.461862	0.359832
C	0.321695	1.314676	-0.843574
C	0.763436	0.328686	0.243281
O	0.692122	1.028019	1.453017
C	0.982402	0.260430	2.601761
C	2.183472	-0.119517	-0.071643
C	2.443285	-1.319877	-0.729614
C	3.749399	-1.669971	-1.057049
C	4.804188	-0.823932	-0.733266
C	4.548271	0.378435	-0.080602
C	3.244273	0.730457	0.244882
O	-0.036655	-0.828769	0.260334
H	-5.094612	-0.987620	-0.661824
H	-3.748956	-1.800249	-1.503252
H	-3.923049	-1.235118	1.504149
H	-4.060072	-2.828938	0.742510
H	-1.696272	-2.389608	1.623371
H	-1.729563	-2.650361	-0.126106
H	0.543562	0.891723	-1.833821
H	0.878510	2.252833	-0.734432
H	0.981085	0.956862	3.444421
H	0.222767	-0.515562	2.773881
H	1.967139	-0.225355	2.531039
H	1.616949	-1.983106	-0.973201
H	3.942959	-2.613874	-1.564344
H	5.825931	-1.100516	-0.987871
H	5.369170	1.044778	0.179059
H	3.036960	1.665343	0.765460

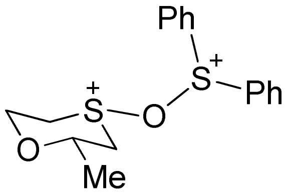

CYCLICS_OSPh2_EQ

B3LYP/6-31G*Geometry

C	-2.558188	-1.938194	-0.645817
S	-1.805846	-0.274571	-0.673696
C	-2.782702	0.508361	0.658839
O	-0.331758	-0.527912	0.070968
S	1.166182	-0.098510	-0.882743
C	1.440053	1.570987	-0.404674
C	1.720081	2.433484	-1.482814
C	1.998333	3.771124	-1.207139
C	1.960098	4.235922	0.109885
C	1.640050	3.371931	1.169330
C	1.380112	2.028687	0.926024
C	2.288098	-1.178626	-0.092982
C	2.374246	-2.455444	-0.697913
C	3.266749	-3.383051	-0.179195
C	4.070493	-3.040461	0.916196
C	3.992029	-1.765821	1.497115
C	3.103447	-0.821706	1.001282
H	-2.279249	-2.405049	0.302945
H	-2.143387	-2.492324	-1.492355
H	-2.511381	0.013071	1.595715
H	-2.493490	1.562206	0.674744
H	1.738424	2.067732	-2.505534
H	2.234966	4.447855	-2.021753
H	2.168741	5.281136	0.316928
H	1.594275	3.752000	2.185073
H	1.111801	1.360884	1.737924
H	1.764742	-2.705645	-1.561670
H	3.350246	-4.364797	-0.633679
H	4.776342	-3.765329	1.310553
H	4.636911	-1.508248	2.331004
H	3.069821	0.172118	1.429326
C	-4.301736	0.315949	0.359285
C	-4.096393	-1.771529	-0.753517
O	-4.610680	-1.066747	0.348133

H	-4.378137	-1.301961	-1.707263
H	-4.527308	-2.776334	-0.729537
H	-4.542885	0.763863	-0.617232
C	-5.124351	0.983895	1.455025
H	-4.933989	2.060637	1.488368
H	-6.184842	0.826729	1.241365
H	-4.899975	0.544969	2.431718

M06/6-31G*Geometry

C	-2.516393	-1.895949	-0.926747
S	-1.718224	-0.283552	-0.701044
C	-2.575291	0.229231	0.811025
O	-0.239441	-0.722476	-0.093898
S	1.171094	-0.124679	-0.987420
C	1.247292	1.542207	-0.469162
C	1.391845	2.473421	-1.507964
C	1.477937	3.819742	-1.178510
C	1.388340	4.214362	0.153808
C	1.210758	3.275138	1.176474
C	1.142643	1.924853	0.878593
C	2.365656	-1.061512	-0.126599
C	2.419174	-2.418778	-0.495045
C	3.370747	-3.228304	0.097515
C	4.273333	-2.683071	1.013498
C	4.231330	-1.327940	1.345110
C	3.276255	-0.498656	0.780257
H	-2.203640	-2.526170	-0.084294
H	-2.159302	-2.307190	-1.877524
H	-2.269640	-0.466402	1.604857
H	-2.239057	1.248899	1.034322
H	1.450612	2.151115	-2.546818
H	1.608203	4.560809	-1.962991
H	1.448775	5.270999	0.405121
H	1.132560	3.605235	2.209598
H	1.004062	1.181881	1.663430
H	1.723967	-2.828965	-1.226727
H	3.424193	-4.281862	-0.164077
H	5.031846	-3.320980	1.461712
H	4.956577	-0.914599	2.041427
H	3.264957	0.563097	1.012116
C	-4.102236	0.144492	0.580216
C	-4.036890	-1.671182	-0.913739
O	-4.462618	-1.189628	0.323064
H	-4.348418	-1.008623	-1.739847
H	-4.510995	-2.645829	-1.075535
H	-4.383341	0.782786	-0.278704
C	-4.819330	0.601186	1.829168
H	-4.587008	1.645655	2.063608
H	-5.899029	0.514035	1.672801
H	-4.545779	-0.030808	2.682234

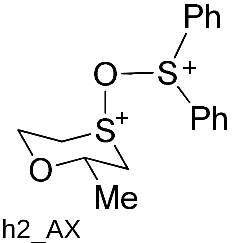

CYCLICS_OSPh2_AX

B3LYP/6-31G*Geometry

C	3.180595	0.108043	-1.072307
S	1.703293	-0.987924	-0.995220
C	2.229640	-2.159672	0.315929
O	0.620854	-0.022702	-0.164999
S	-1.009013	0.202401	-0.965165
C	-1.933165	-1.140122	-0.308748
C	-2.647600	-1.867040	-1.282177
C	-3.447863	-2.928628	-0.863387
C	-3.499216	-3.273810	0.489399
C	-2.750467	-2.564588	1.442715
C	-1.962215	-1.487428	1.056697
C	-1.455702	1.715130	-0.218679
C	-1.027164	2.854554	-0.941951
C	-1.368334	4.114439	-0.470698
C	-2.136806	4.238462	0.694455
C	-2.574940	3.103387	1.393309
C	-2.242115	1.831964	0.946894
H	2.868799	0.974363	-1.663084
H	1.328468	-2.708468	0.605256
H	-2.594823	-1.600336	-2.333928
H	-4.020673	-3.488818	-1.595262
H	-4.116550	-4.108289	0.807940
H	-2.786126	-2.856823	2.487549
H	-1.368677	-0.947689	1.787220
H	-0.453828	2.749940	-1.858815
H	-1.053443	4.998399	-1.015527
H	-2.413116	5.226002	1.051716
H	-3.189330	3.215493	2.280764
H	-2.613311	0.958410	1.467349
H	2.912098	-2.840978	-0.207819
H	3.902114	-0.479235	-1.655654
C	3.747824	0.467593	0.315477
C	2.933002	-1.447252	1.479920
O	4.054830	-0.720515	1.034164
H	2.999937	1.055062	0.868491
H	3.300512	-2.216100	2.165894
H	2.225889	-0.806449	2.025238
C	5.035267	1.267293	0.157409
H	5.425926	1.509115	1.149359
H	5.791737	0.684456	-0.376803
H	4.855992	2.202106	-0.382597

M06/6-31G*Geometry

C	3.184869	0.114301	-1.071913
S	1.687866	-0.922848	-0.982937
C	2.114425	-1.953199	0.455147
O	0.629425	0.153053	-0.293833
S	-0.973390	0.201993	-1.057013
C	-1.743204	-1.204217	-0.361657
C	-2.369520	-2.048881	-1.290237
C	-3.022648	-3.179866	-0.817006
C	-3.014991	-3.466230	0.545368
C	-2.356977	-2.630599	1.456595
C	-1.717781	-1.485106	1.015161
C	-1.553123	1.643855	-0.266825
C	-1.054892	2.843009	-0.811286
C	-1.505901	4.043176	-0.293820
C	-2.462492	4.044480	0.724265
C	-2.974111	2.849501	1.233091
C	-2.526384	1.633304	0.744832
H	2.956314	0.908894	-1.792918
H	1.195434	-2.488316	0.725534
H	-2.359968	-1.818166	-2.354845
H	-3.529829	-3.841306	-1.514663
H	-3.520644	-4.358189	0.908805
H	-2.352920	-2.878618	2.515384
H	-1.200080	-0.826429	1.712415

H	-0.329891	2.830290	-1.624332
H	-1.130160	4.981559	-0.693508
H	-2.829981	4.991873	1.112788
H	-3.737561	2.869213	2.006537
H	-2.952235	0.704408	1.114492
H	2.841067	-2.676692	0.055986
H	3.939535	-0.555962	-1.513677
C	3.646392	0.625021	0.295789
C	2.717054	-1.124588	1.582543
O	3.873505	-0.463754	1.162712
H	2.869397	1.286623	0.719093
H	3.004472	-1.809830	2.388368
H	1.969799	-0.420502	1.986794
C	4.950005	1.371123	0.131978
H	5.284404	1.734308	1.108856
H	5.724636	0.705157	-0.266479
H	4.838808	2.230475	-0.538607

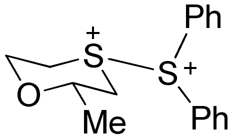

CYCLICS_SPh2_EQ
B3LYP/6-31G*Geometry

C	0.810735	2.774831	0.840926
C	1.299053	4.029126	0.510399
C	2.392150	4.145665	-0.362380
C	3.008083	3.003154	-0.900237
C	2.531853	1.737391	-0.593441
C	1.426202	1.623878	0.280710
S	0.755404	0.119077	0.860568
C	1.708433	-1.260738	0.327628
C	2.075738	-1.503059	-1.012868
C	2.825744	-2.636785	-1.298852
C	3.204632	-3.515082	-0.269439
C	2.816505	-3.277880	1.052936
C	2.045988	-2.160576	1.363507
S	-1.309921	-0.266114	-0.593454
C	-2.603989	0.853859	0.048104
C	-1.950472	-1.848455	0.056508
H	-0.011905	2.677717	1.544174
H	0.847130	4.916559	0.941429
H	2.778353	5.129730	-0.610800
H	3.870834	3.107541	-1.550303
H	3.032372	0.860225	-0.982715
H	1.765587	-0.836642	-1.811298
H	3.112939	-2.846131	-2.324492
H	3.794172	-4.395327	-0.507414
H	3.108675	-3.963421	1.841900
H	1.741454	-1.971478	2.388788
H	-2.568345	0.874442	1.140985
H	-2.401646	1.843221	-0.369505
H	-1.330938	-2.645067	-0.362874
H	-1.894988	-1.847046	1.148707
C	-3.439175	-1.985793	-0.386622
C	-4.011730	0.318884	-0.402755
H	-4.040169	0.256198	-1.499586
H	-3.806574	-2.931205	0.023848
H	-3.519842	-2.019011	-1.480922
O	-4.216361	-0.957837	0.168534
C	-5.097362	1.260250	0.107125
H	-4.975644	2.262090	-0.314539
H	-6.070828	0.871101	-0.204348
H	-5.081806	1.319780	1.199372

M06/6-31G*Geometry

C	0.384213	2.750948	0.946400
C	0.691096	4.061580	0.626277
C	1.662955	4.329402	-0.340140
C	2.340900	3.291561	-0.989369
C	2.046644	1.972470	-0.695026
C	1.063316	1.713044	0.273635
S	0.614841	0.116349	0.822818
C	1.790200	-1.089823	0.307162
C	2.117071	-1.331851	-1.035615
C	3.061735	-2.306983	-1.310764
C	3.668933	-3.015072	-0.267920
C	3.322707	-2.772779	1.058407
C	2.361149	-1.817001	1.362450
S	-1.125498	-0.486418	-0.598155
C	-2.485762	0.574988	-0.004392
C	-1.660271	-2.060931	0.158677
H	-0.343566	2.530055	1.727283
H	0.190361	4.877432	1.141406
H	1.909460	5.361458	-0.580584
H	3.111331	3.519099	-1.721777
H	2.595531	1.166814	-1.175208
H	1.637389	-0.785229	-1.847238
H	3.327918	-2.522545	-2.342827
H	4.413474	-3.773739	-0.498284
H	3.795341	-3.332875	1.861355
H	2.078478	-1.624991	2.396557
H	-2.443179	0.634247	1.092502
H	-2.341995	1.561468	-0.461036
H	-0.973170	-2.836072	-0.199606
H	-1.608051	-1.964026	1.251260
C	-3.113728	-2.309478	-0.283059
C	-3.829459	-0.070361	-0.426677
H	-3.847805	-0.196642	-1.524383
H	-3.425920	-3.263789	0.157963
H	-3.182620	-2.403828	-1.379560
O	-3.959448	-1.317430	0.207933
C	-4.965913	0.817654	0.025321
H	-4.912664	1.804539	-0.446702
H	-5.915191	0.350935	-0.256792
H	-4.953747	0.934025	1.115392

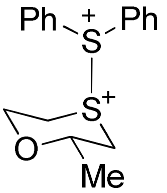

CYCLICS_SPh2_AX
B3LYP/6-31G*Geometry

C	1.762821	-2.224886	-0.984889
C	2.841159	-3.090938	-0.865706
C	3.942752	-2.729236	-0.078130
C	3.977305	-1.492055	0.586920
C	2.910594	-0.610033	0.488361
C	1.801786	-0.981806	-0.301886
S	0.406150	0.030012	-0.619434
C	0.718993	1.730328	-0.251445
C	1.149173	2.213683	0.999621

C	1.377044	3.578382	1.135447
C	1.183651	4.442614	0.045696
C	0.739609	3.952433	-1.185297
C	0.482668	2.591873	-1.343643
S	-1.067981	-0.454058	1.240620
C	-1.533404	-2.214272	0.968223
C	-2.629272	0.409895	0.756332
H	0.922320	-2.484035	-1.622976
H	2.834274	-4.037745	-1.395979
H	4.786892	-3.406932	0.008078
H	4.846919	-1.216482	1.174901
H	2.957006	0.353840	0.978694
H	1.281277	1.552988	1.850177
H	1.701315	3.974479	2.092603
H	1.369162	5.505577	0.166449
H	0.584884	4.626391	-2.021760
H	0.133473	2.207044	-2.297466
H	-0.596500	-2.778869	0.917683
H	-2.045937	-2.486989	1.899727
H	-2.351394	1.454517	0.585110
H	-3.233135	0.358866	1.672067
C	-3.380434	-0.233027	-0.425824
C	-2.451092	-2.421631	-0.244449
H	-2.790489	-3.461698	-0.231082
H	-1.901748	-2.253679	-1.184169
H	-2.781537	-0.116677	-1.343933
O	-3.601086	-1.614115	-0.177513
C	-4.739399	0.434096	-0.601179
H	-5.255167	-0.026806	-1.447673
H	-5.355386	0.296587	0.292512
H	-4.631969	1.504764	-0.800754

M06/6-31G*Geometry

C	2.260297	-1.815226	-0.937579
C	3.524962	-2.361000	-0.801068
C	4.488107	-1.696258	-0.039735
C	4.199826	-0.477597	0.584621
C	2.940937	0.085062	0.472456
C	1.977437	-0.594608	-0.289252
S	0.363092	0.003745	-0.601481
C	0.234526	1.729841	-0.263732
C	0.507742	2.307802	0.984969
C	0.374739	3.681651	1.109089
C	-0.015172	4.454961	0.010659
C	-0.299787	3.863255	-1.216707
C	-0.195671	2.485213	-1.364461
S	-0.806115	-0.753904	1.235393
C	-1.001914	-2.529790	0.826333
C	-2.497120	-0.137317	0.868103
H	1.513133	-2.302961	-1.564279
H	3.769744	-3.293661	-1.302993
H	5.483617	-2.124289	0.057377
H	4.970619	0.037726	1.152186
H	2.728615	1.047556	0.929911
H	0.800093	1.704880	1.844464
H	0.572402	4.156625	2.067141
H	-0.108974	5.532798	0.122463
H	-0.610450	4.471875	-2.062248
H	-0.421917	2.010879	-2.318764
H	0.012876	-2.940697	0.737508
H	-1.455933	-2.948515	1.737024
H	-2.412700	0.956500	0.814553
H	-3.061128	-0.395106	1.778894
C	-3.147045	-0.769950	-0.362222
C	-1.884571	-2.766874	-0.391909
H	-2.043308	-3.848017	-0.484650
H	-1.381548	-2.426371	-1.315802
H	-2.591319	-0.465772	-1.271973
O	-3.138767	-2.173443	-0.250807
C	-4.585492	-0.316808	-0.456344
H	-5.049853	-0.768371	-1.338699
H	-5.147732	-0.642078	0.426943
H	-4.658543	0.772946	-0.544517

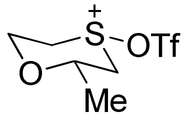

CYCLICS_OTf_EQ

B3LYP/6-31G*Geometry

C	-1.961389	1.012328	-0.169907
S	-1.015564	-0.517740	-0.475694
C	-1.852688	-1.621252	0.708894
O	0.408100	-0.222470	0.311471
S	1.865602	-0.318487	-0.712849
O	2.179856	-1.722462	-0.860210
O	1.634192	0.588236	-1.819758
C	2.969991	0.490958	0.619906
F	2.902468	-0.245792	1.710239
F	4.187614	0.504545	0.117572
F	2.520605	1.712096	0.833225
H	-1.609568	1.739018	-0.907190
H	-1.720865	1.344661	0.844332
H	-1.445956	-2.623189	0.546513
H	-1.609179	-1.263566	1.713045
O	-3.873526	-0.247571	0.661570
C	-3.478627	0.711154	-0.309508
H	-3.684587	0.323660	-1.319921
C	-3.372732	-1.545475	0.439574
H	-3.862754	-2.208396	1.157578
H	-3.612999	-1.903884	-0.572619
C	-4.282476	1.983778	-0.072114
H	-4.029784	2.749553	-0.811415
H	-5.347060	1.752385	-0.160308
H	-4.095499	2.376942	0.931693

M06/6-31G*Geometry

C	-2.006377	1.059038	0.355175
S	-0.816241	-0.055385	-0.436473
C	-1.636399	-1.620367	-0.035251
O	0.370276	0.051083	0.712835
S	1.862742	-0.723480	0.366478
O	2.371887	-1.091598	1.650695
O	1.582523	-1.619933	-0.728230
C	2.775034	0.773251	-0.283405
F	2.908588	1.630839	0.691068
F	3.928636	0.355379	-0.726147
F	2.046411	1.277708	-1.259631
H	-1.669843	2.080936	0.147199
H	-1.966442	0.853292	1.433652
H	-1.055905	-2.416386	-0.515600
H	-1.609870	-1.723308	1.057472
O	-3.798339	-0.542923	0.115777
C	-3.412012	0.772229	-0.205942
H	-3.404204	0.900104	-1.305364
C	-3.075126	-1.532305	-0.553711
H	-3.560081	-2.493768	-0.353043

H	-3.093213	-1.375101	-1.646949
C	-4.405086	1.720570	0.423677
H	-4.163987	2.763211	0.189997
H	-5.406317	1.500053	0.040855
H	-4.417844	1.589938	1.512217

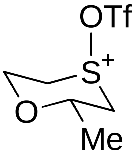

CYCLICS_OTf_AX

B3LYP/6-31G*Geometry

C	1.370646	-0.216155	1.827909
S	0.991434	-1.322235	0.417631
C	2.453583	-1.013949	-0.648471
O	-0.109142	-0.429911	-0.455609
S	-1.811779	-0.811423	-0.170227
O	-2.303223	-1.474407	-1.352808
O	-1.861355	-1.357436	1.174876
C	-2.346712	1.016018	-0.161181
F	-2.061143	1.538925	-1.335621
F	-3.640550	1.028786	0.077691
F	-1.675813	1.625710	0.808385
H	0.444147	-0.150469	2.406466
H	2.195992	-1.453128	-1.616755
H	3.245043	-1.612496	-0.180084
H	2.114795	-0.775391	2.408484
O	3.080064	0.988523	0.579368
C	1.916399	1.139209	1.364553
H	2.203234	1.707981	2.253624
H	1.138326	1.702316	0.831609
C	2.850259	0.473097	-0.727444
H	2.039438	1.034863	-1.212415
C	4.140250	0.621968	-1.523935
H	4.411236	1.680045	-1.568318
H	4.958636	0.076371	-1.043947
H	4.014900	0.252359	-2.546412

M06/6-31G*Geometry

C	1.263236	0.045628	1.803328
S	0.954001	-1.265265	0.585132
C	2.433140	-1.089326	-0.458857
O	-0.108452	-0.521688	-0.439526
S	-1.780010	-0.822076	-0.143760
O	-2.292714	-1.515165	-1.285465
O	-1.845556	-1.283914	1.220790
C	-2.260335	0.984015	-0.226649
F	-1.885365	1.458126	-1.385486
F	-3.554734	1.034990	-0.076001
F	-1.642333	1.605833	0.756643
H	0.317161	0.170424	2.345334
H	2.223024	-1.682242	-1.357948
H	3.223803	-1.590228	0.121210
H	2.001909	-0.393994	2.489605
O	2.980757	1.072334	0.455318
C	1.791334	1.314762	1.151513
H	2.014999	2.036994	1.944816
H	1.024888	1.760701	0.497116
C	2.808654	0.363705	-0.753216
H	2.007435	0.831160	-1.352109
C	4.117692	0.399136	-1.505827
H	4.385810	1.439126	-1.716792
H	4.919103	-0.044920	-0.903227
H	4.045285	-0.140177	-2.457053

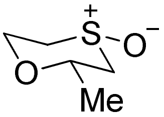

CYCLICS_O_EQ

B3LYP/6-31G*Geometry

C	-0.990371	1.313361	0.324241
S	-1.395032	-0.329163	-0.415380
C	0.178918	-1.107518	0.161353
H	-1.793637	1.994857	0.025495
H	0.176091	-2.125380	-0.242041
H	0.129201	-1.160091	1.254911
H	-1.013156	1.191391	1.412560
O	1.440696	0.982971	0.265524
C	0.372923	1.812001	-0.159244
H	0.571848	2.802304	0.261939
H	0.375530	1.908202	-1.258116
C	1.415887	-0.325134	-0.302303
H	1.390276	-0.235498	-1.402632
C	2.710002	-1.014110	0.115111
H	3.570052	-0.425124	-0.217698
H	2.757352	-1.108036	1.205044
H	2.776875	-2.013812	-0.326342
O	-2.533705	-0.937197	0.371728

M06/6-31G*Geometry

C	-0.977651	1.294853	0.330726
S	-1.384257	-0.325941	-0.421038
C	0.174991	-1.090523	0.164660
H	-1.789004	1.980784	0.058810
H	0.176752	-2.119889	-0.215814
H	0.119351	-1.120913	1.262986
H	-0.987666	1.147482	1.419469
O	1.433131	0.978930	0.261375
C	0.369584	1.795189	-0.157937
H	0.565720	2.795238	0.246896
H	0.364016	1.880133	-1.261929
C	1.402056	-0.318409	-0.301528
H	1.367820	-0.226521	-1.407029
C	2.679326	-1.012459	0.112667
H	3.549276	-0.428381	-0.207226
H	2.714425	-1.110575	1.205043
H	2.743393	-2.013234	-0.330352
O	-2.503857	-0.926554	0.372904

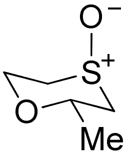

CYCLICS_O_AX

B3LYP/6-31G*Geometry

C	1.197008	1.183118	-0.560646
S	1.424454	-0.640879	-0.321313
C	-0.359232	-1.038754	-0.641711
O	1.660843	-0.857925	1.161545
H	2.162747	1.634465	-0.311800
H	-0.451037	-2.114219	-0.459559
H	-0.562307	-0.833917	-1.699590

H	0.977435	1.356921	-1.619975
O	-1.181217	1.162586	0.008052
C	0.085952	1.710552	0.341979
H	-0.010536	2.794565	0.223295
H	0.339819	1.494985	1.390151
C	-1.291063	-0.237990	0.272180
H	-0.992641	-0.435075	1.313130
C	-2.753729	-0.612736	0.069531
H	-3.386168	-0.015701	0.733856
H	-3.061964	-0.417540	-0.963530
H	-2.917233	-1.672833	0.290257

M06/6-31G*Geometry

C	1.184943	1.162294	-0.573586
S	1.413566	-0.645781	-0.314917
C	-0.358852	-1.028441	-0.634005
O	1.626770	-0.833785	1.161423
H	2.156233	1.619164	-0.348927
H	-0.464659	-2.106547	-0.459183
H	-0.567527	-0.812871	-1.692295
H	0.944393	1.323818	-1.633032
O	-1.165828	1.157879	0.014866
C	0.096614	1.691964	0.335761
H	0.010608	2.780317	0.229747
H	0.362005	1.468342	1.383563
C	-1.277191	-0.230418	0.276942
H	-0.978130	-0.432345	1.322135
C	-2.726205	-0.600994	0.062918
H	-3.369113	-0.003663	0.719106
H	-3.018367	-0.399580	-0.976149
H	-2.895892	-1.663319	0.275212

References

[1] W. L. F. Armarego and D. D. Perrin, *Purification of Laboratory Chemicals*, Butterworth-Heinemann, **1996**, p. 529 pages.

[2] J. Drabowicz, A. Kotynski and Z. H. Kudzin, *J. Chromatogr.* **1988**, *447*, 225-229.

[3] M. A. Fascione, S. J. Adshead, S. A. Stalford, C. A. Kilner, A. G. Leach and W. B. Turnbull, *Chem. Commun.* **2009**, 5841-5843.

[4] M. A. Fascione, C. A. Kilner, A. G. Leach and W. B. Turnbull, *Chem. Eur. J.* **2011**, *17*, accepted.

[5] N. Fukuda and T. Ikemoto, *J. Org. Chem.* **2010**, *75*, 4629-4631.

[6] W. T. Murray, J. W. Kelly and S. A. Evans, *J. Org. Chem.* **1987**, *52*, 525-529.

[7] in *Macromodel*, *Vol.* Schrödinger, LLC, New York, NY, **2007**.

[8] a) W. L. Jorgensen and J. Tirado-Rives, *Journal of the American Chemical Society* **1988**, *110*, 1657-1666; b) W. L. Jorgensen and J. Tirado-Rives, *Proc. Natl. Acad. Sci. U S A* **2005**, *102*, 6665-6670.

[9] M. J. Frisch, G. W. Trucks, H. B. Schlegel, G. E. Scuseria, M. A. Robb, J. R. Cheeseman, G. Scalmani, V. Barone, B. Mennucci, G. A. Petersson, H. Nakatsuji, M. Caricato, X. Li, H. P. Hratchian, A. F. Izmaylov, J. Bloino, G. Zheng, J. L. Sonnenberg, M. Hada, M. Ehara, K. Toyota, R. Fukuda, J. Hasegawa, M. Ishida, T. Nakajima, Y. Honda, O. Kitao, H. Nakai, T. Vreven, J. Montgomery, J. A., J. E. Peralta, F. Ogliaro, M. Bearpark, J. J. Heyd, E. Brothers, K. N. Kudin, V. N. Staroverov, R. Kobayashi, J. Normand, K. Raghavachari, A. Rendell, J. C. Burant, S. S. Iyengar, J. Tomasi, M. Cossi, N. Rega, N. J. Millam, M. Klene, J. E. Knox, J. B. Cross, V. Bakken, C. Adamo, J. Jaramillo, R. Gomperts, R. E. Stratmann, O. Yazyev, A. J. Austin, R. Cammi, C. Pomelli, J. W. Ochterski, R. L. Martin, K. Morokuma, V. G. Zakrzewski, G. A. Voth, P. Salvador, J. J. Dannenberg, S. Dapprich, A. D. Daniels, Ö. Farkas, J. B. Foresman, J. V. Ortiz, J. Cioslowski and D. J. Fox in *Gaussian 09, Revision A.1*, *Vol.* Gaussian, Inc., Wallingford CT, **2009**.

[10] a) A. D. Becke, *J. Chem. Phys.* **1993**, *98*, 1372-1377; b) C. Lee, W. Yang and R. G. Parr, *Phys. Rev. B: Condens. Matter* **1988**, *37*, 785-789; c) P. C. Hariharan and J. A. Pople, *Theor. Chim. Acta* **1973**, *28*, 213-222.

[11] Y. Zhao and D. Truhlar, *Theor. Chem. Account.* **2008**, *120*, 215-241.

[12] a) C. Adamo and V. Barone, *J. Chem. Phys.* **1998**, *108*, 664-675; b) A. D. Becke, *J. Chem. Phys.* **1996**, *104*, 1040-1046.

[13] M. J. Frisch, G. W. Trucks, H. B. Schlegel, G. E. Scuseria, M. A. Robb, J. R. Cheeseman, J. J. A. Montgomery, T. Vreven, K. N. Kudin, J. C. Burant, J. M. Millam, S. S. Iyengar, J. Tomasi, V. Barone, B. Mennucci, M. Cossi, G. Scalmani, N. Rega, G. A. Petersson, H. Nakatsuji, M. Hada, M. Ehara, K. Toyota, R. Fukuda, J. Hasegawa, M. Ishida, T. Nakajima, Y. Honda, O. Kitao, H. Nakai, M. Klene, X. Li, J. E. Knox, H. P. Hratchian, J. B. Cross, V. Bakken, C. Adamo, J. Jaramillo, R. Gomperts, R. E. Stratmann, O. Yazyev, A. J. Austin, R. Cammi, C. Pomelli, J. W. Ochterski, P. Y. Ayala, K. Morokuma, G. A. Voth, P. Salvador, J. J. Dannenberg, V. G. Zakrzewski, S. Dapprich, A. D. Daniels, M. C. Strain, O. Farkas, D. K. Malick, A. D. Rabuck, K. Raghavachari, J. B. Foresman, J. V. Ortiz, Q. Cui, A. G. Baboul, S. Clifford, J. Cioslowski, B. B. Stefanov, G. Liu, A. Liashenko, P. Piskorz, I. Komaromi, R. L. Martin, D. J. Fox, T. Keith, M. A. Al-Laham, C. Y. Peng, A. Nanayakkara, M. Challacombe, P. M. W. Gill, B. Johnson, W. Chen, M. W. Wong, C. Gonzalez and J. A. Pople in *Gaussian03: Revision D.02*, *Vol.* Gaussian, Inc., Wallingford CT, **2004**.

[14] J. Tomasi, B. Mennucci and R. Cammi, *Chem. Rev.* **2005**, *105*, 2999-3094.

[15] a) W. K. Musker, *Acc. Chem. Res.* **1980**, *13*, 200-206; b) N. Furukawa, K. Kobayashi and S. Sato, *J. Organomet. Chem.* **2000**, *611*, 116-126; c) V. G. Nenajdenko, N. E. Shevchenko, E. S. Balenkova and I. V. Alabugin, *Chem. Rev.* **2003**, *103*, 229-282.

[16] N. Furukawa and S. Sato, *Top. Curr. Chem.* **1999**, *205*, 89-129.

[17] M. Jacobsson, J. Oxgaard, C. O. Abrahamsson, P. O. Norrby, W. Goddard and U. Ellervik, *Chem. Eur. J.* **2008**, *14*, 3954-3960.

Parameter	Value
1 Solvent	CDCl3
2 NAME	Sam64644
3 INSTRUM	avance500
4 Temperature	300.0
5 Pulse Sequence	zg30
6 Number of Scans	32
7 Nucleus	1H
8 Spectrometer Frequency	500.23001099
9 Spectral Width	7002.801

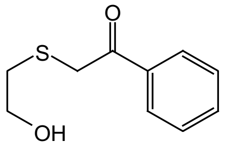

31

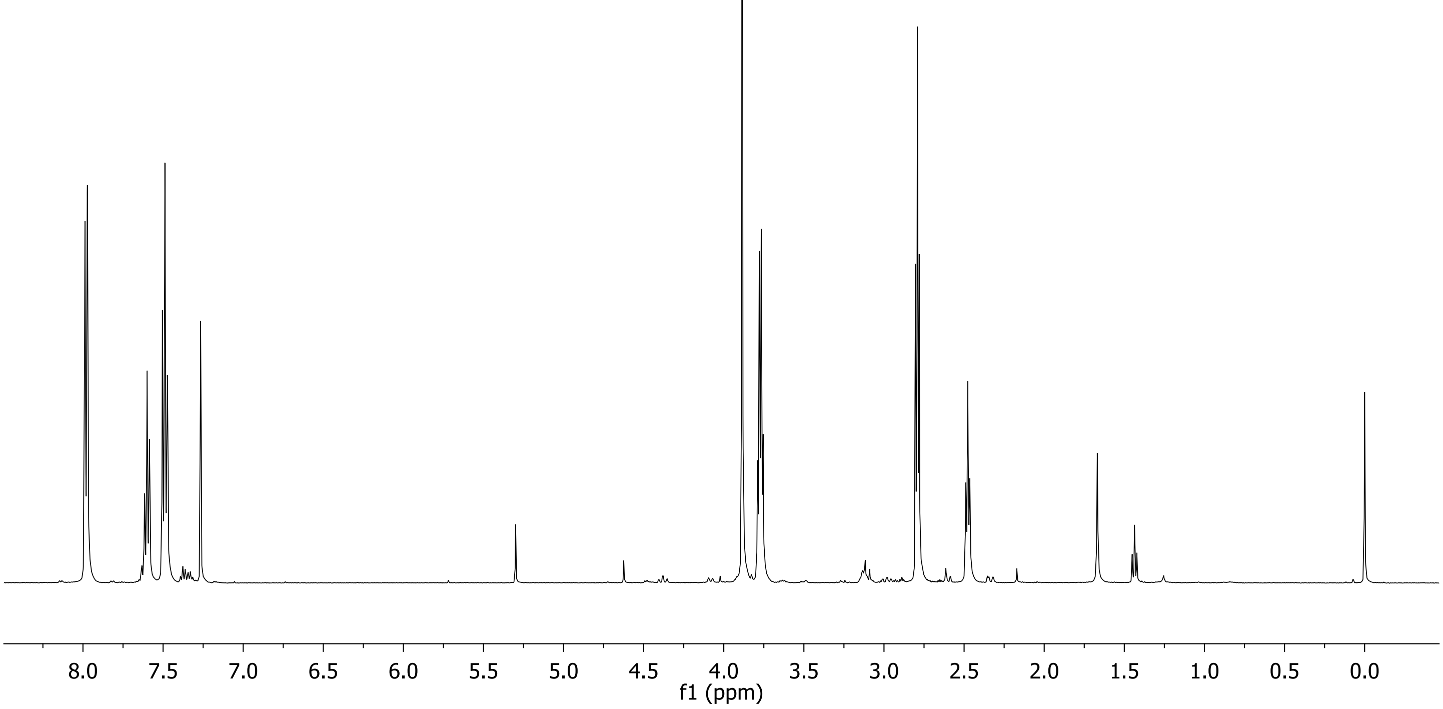

Parameter	Value
1 Solvent	CDCl3
2 NAME	samJ4498
3 INSTRUM	dpx300
4 Temperature	300.0
5 Pulse Sequence	zgpg30
6 Number of Scans	2400
7 Nucleus	13C
8 Spectrometer Frequency	75.47564697
9 Spectral Width	18832.393

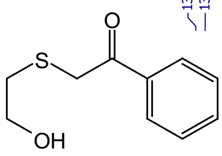

31

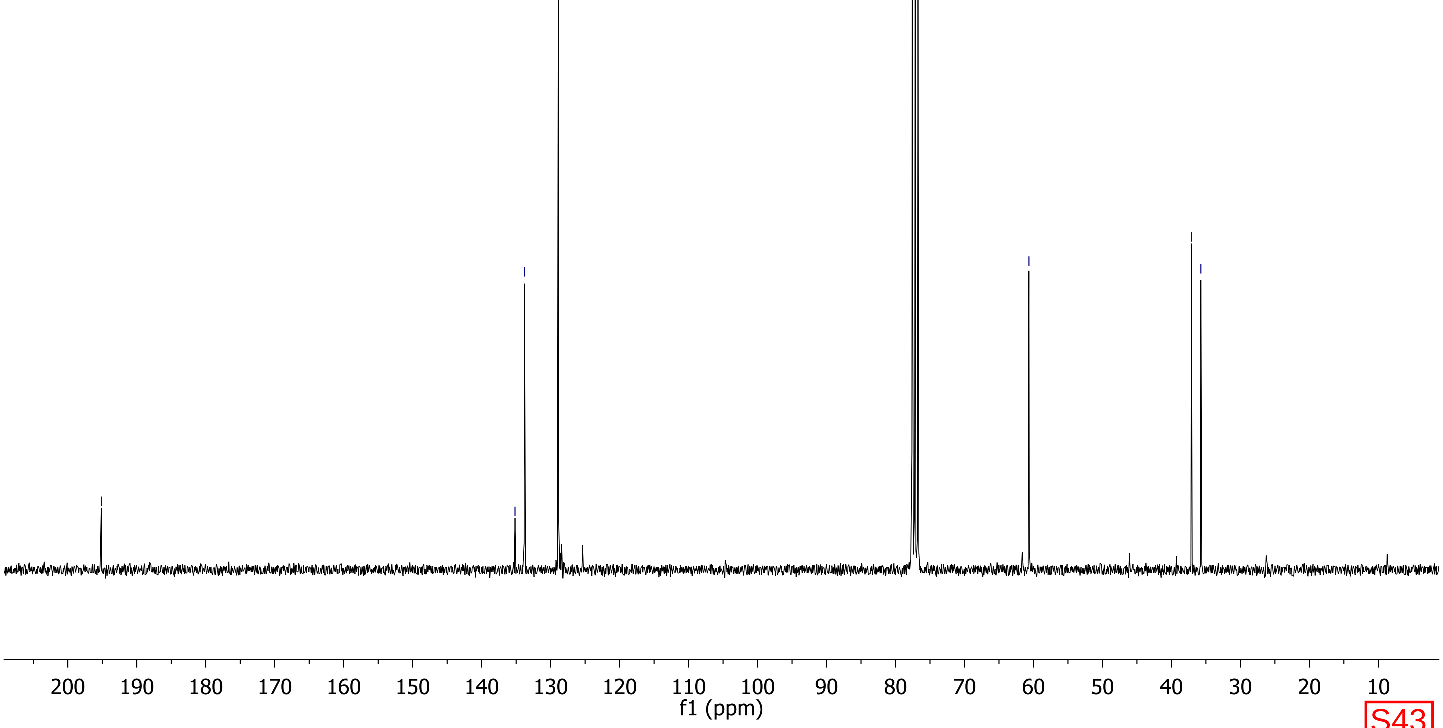

Parameter	Value
1 Solvent	CDCl3
2 NAME	Sam65144
3 INSTRUM	avance500
4 Temperature	300.0
5 Pulse Sequence	zg30
6 Number of Scans	32
7 Nucleus	1H
8 Spectrometer Frequency	500.23004150
9 Spectral Width	7002.801

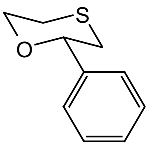

rac-32

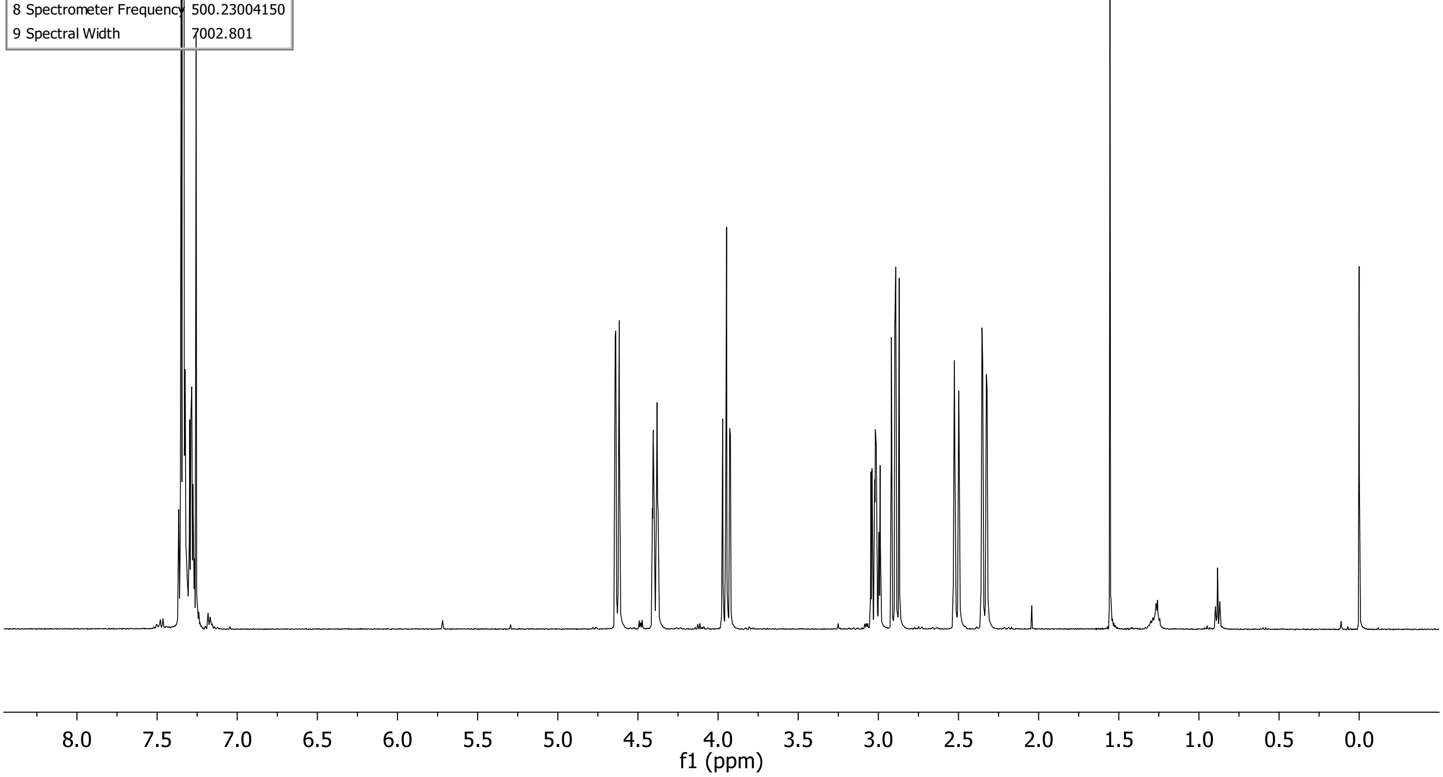

Parameter	Value
1 Solvent	CDCl3
2 NAME	samJ4881
3 INSTRUM	dpx300
4 Temperature	300.0
5 Pulse Sequence	zgpg30
6 Number of Scans	160
7 Nucleus	13C
8 Spectrometer Frequency	75.47564697
9 Spectral Width	18832.393

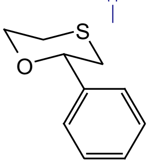

rac-32

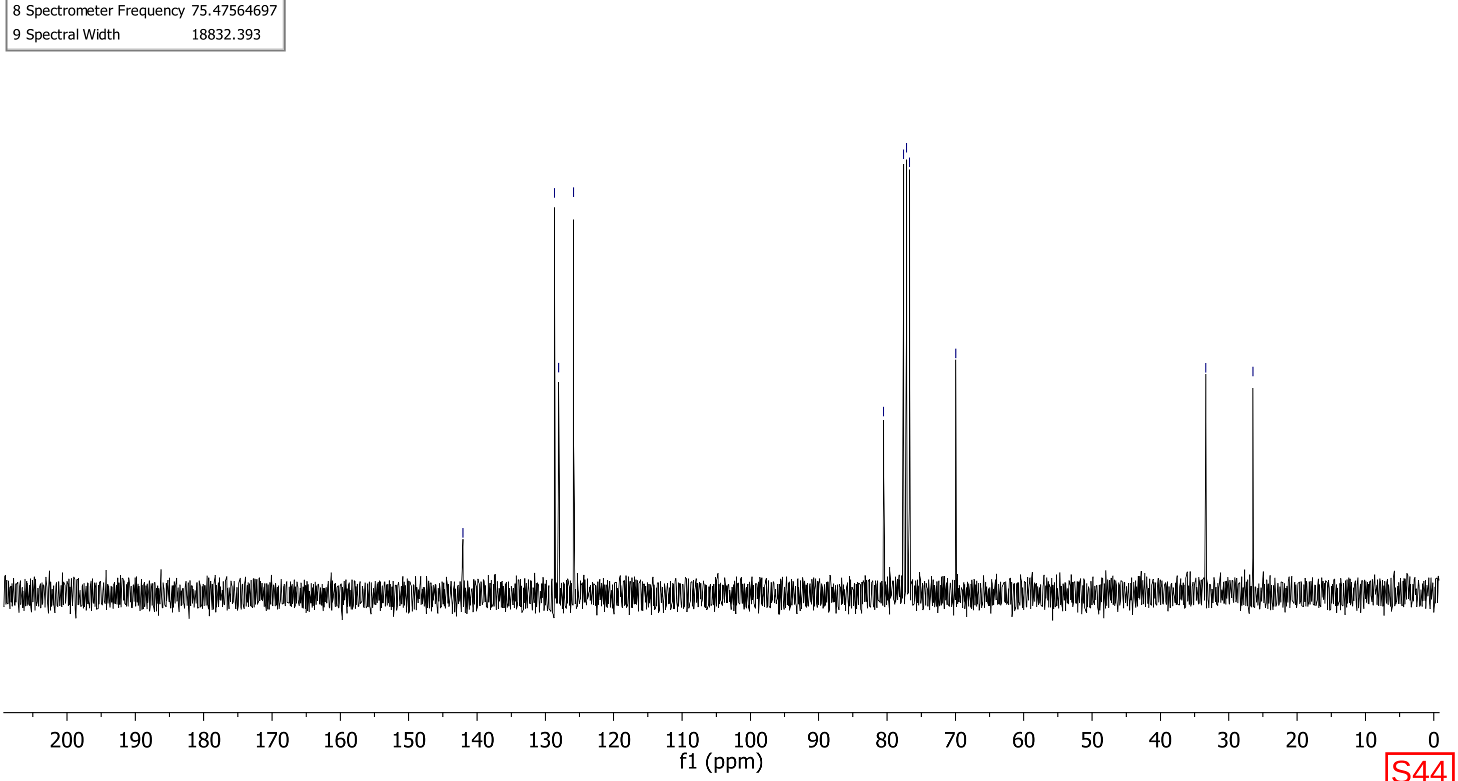

Parameter	Value
1 Solvent	CD3OD
2 NAME	Sam65579
3 INSTRUM	avance500
4 Temperature	300.0
5 Pulse Sequence	zg30
6 Number of Scans	32
7 Nucleus	1H
8 Spectrometer Frequency	500.23275757
9 Spectral Width	7002.801

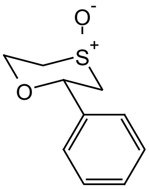

rac-33a

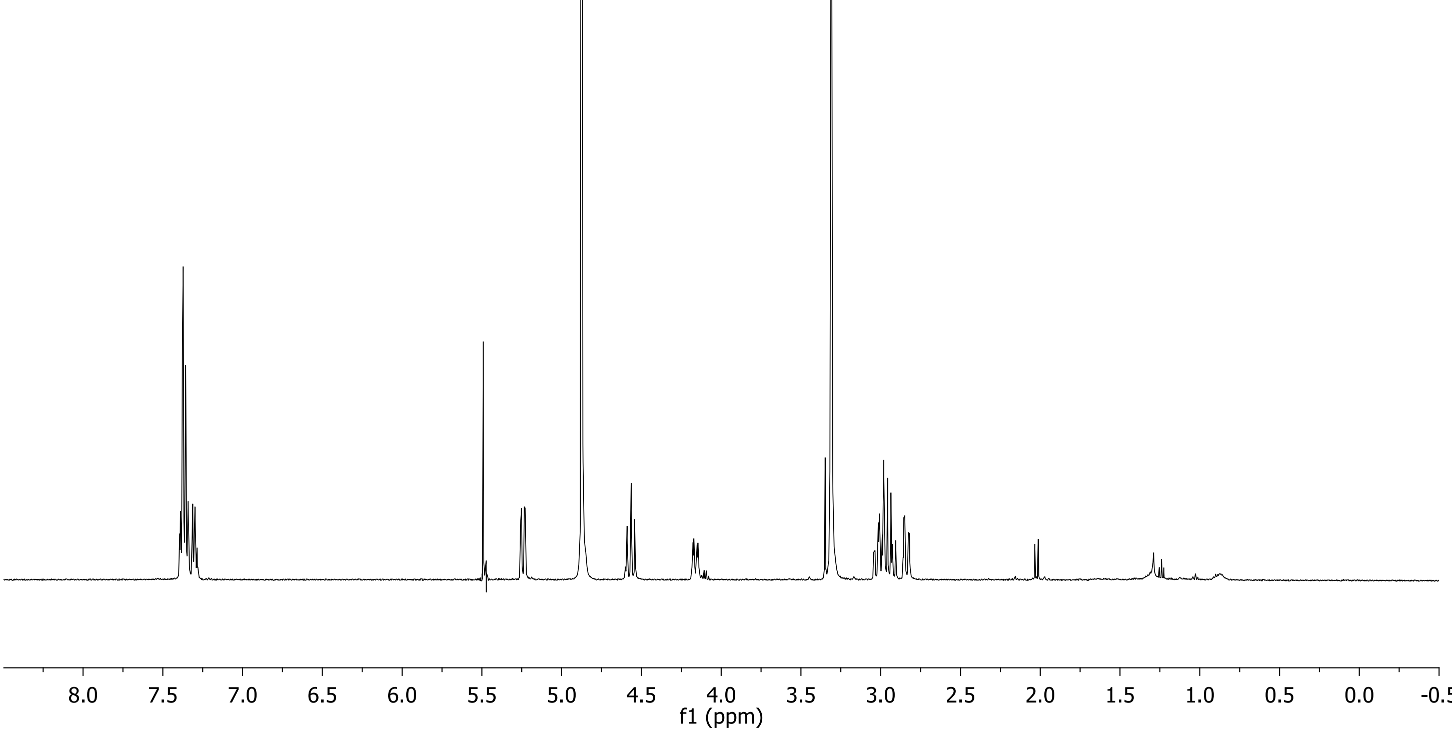

Parameter	Value
1 Solvent	CD3OD
2 NAME	samJ5314
3 INSTRUM	dpx300
4 Temperature	300.0
5 Pulse Sequence	zgpg30
6 Number of Scans	2400
7 Nucleus	13C
8 Spectrometer Frequency	75.47564697
9 Spectral Width	18832.393

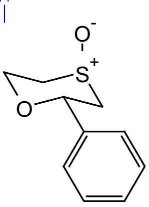

rac-33a

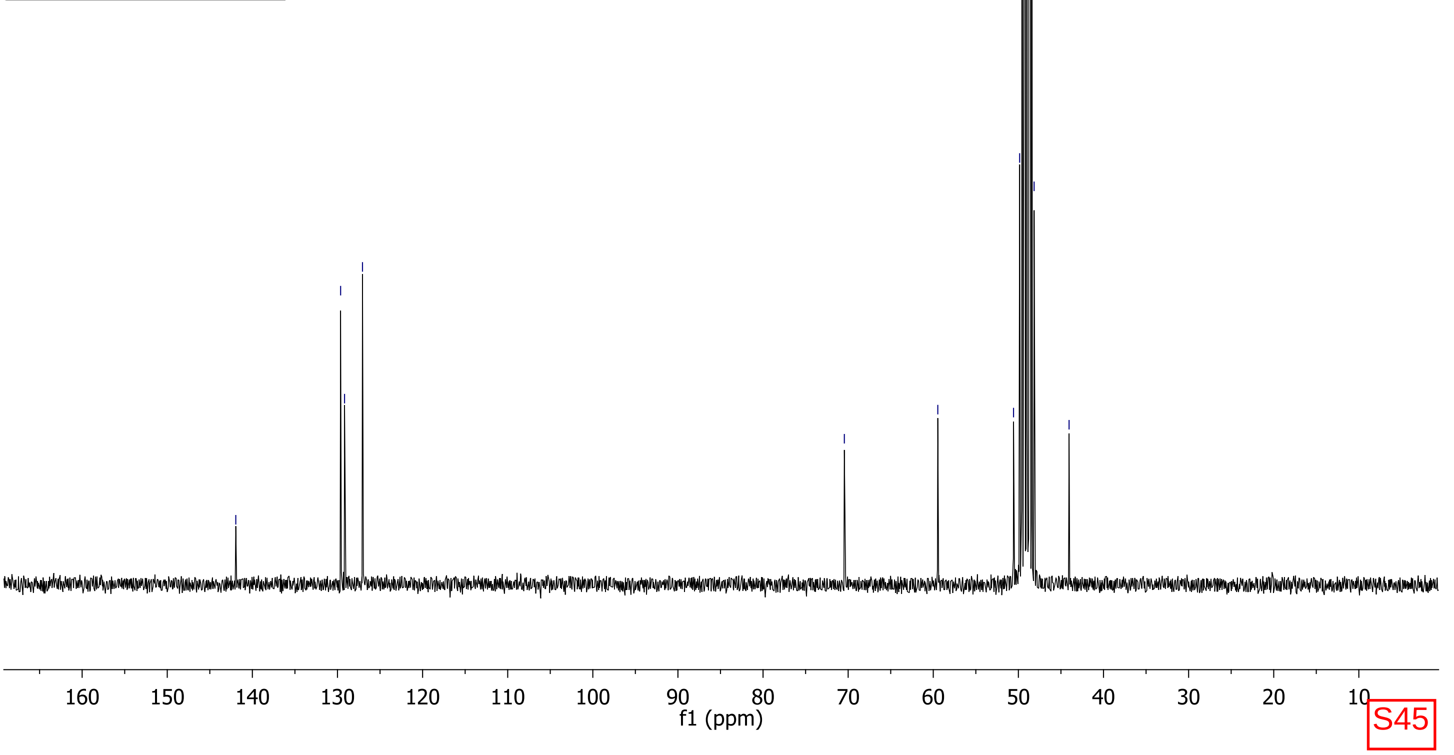

Supplement: Supplementary file 1 [file chem0018-2987-SD1.pdf]
